# Transradial vs Transfemoral Access for Cerebral Angiography: A Randomized Noninferiority Clinical Trial

**DOI:** 10.1001/jamanetworkopen.2026.1929

**Published:** 2026-03-19

**Authors:** Wei Ni, Heng Yang, Jiabin Su, Ya Peng, Dengliang Wang, Zhiqing Lin, Jun Sun, Xuelin Chen, Jiyue Wang, Yi Li, Jiaxiong Wang, Shu Wan, Xin Ye, Qingrong Zhang, Donghai Wang, Chao Gao, Hanqiang Jiang, Xinjie Gao, Yingying Zhang, Bing Han, Jie Cao, Xucheng Zhu, Shengjun Zhou, Yiyong Zeng, Fuxiang Chen, Yuanxiang Lin, Huajun Ba, Xiaoxiang Chen, Xianglu Liu, Jiheng Hao, Zhaoliang Sun, Mei Li, Ming Wang, Dajiang Xie, Zong Zhuang, Lin Shi, Liang Zhou, Hongyu Tang, Dan Chen, Craig S. Anderson, Dezhi Kang, Yuxiang Gu

**Affiliations:** 1 Department of Neurosurgery, Huashan Hospital, Fudan University, Shanghai, China; 2 Department of Neurosurgery, The First People’s Hospital of Changzhou, Changzhou, China; 3 Department of Neurosurgery, The First Affiliated Hospital of Fujian Medical University, Fuzhou, China; 4 Department of Neurosurgery, The First Affiliated Hospital of Ningbo University, Ningbo, China; 5 Department of Neurosurgery, Panvascular Disease Management Center, Wenzhou Central Hospital, Wenzhou, China; 6 Department of Neurosurgery, Affiliated Zhongshan Hospital of Fudan University, Qingpu Branch, Shanghai, China; 7 Department of Neurosurgery, Liaocheng People’s Hospital, Liaocheng, China; 8 Department of Neurosurgery, Shanghai Ninth People’s Hospital, Shanghai Jiao Tong University School of Medicine, Shanghai, China; 9 Department of Neurosurgery, Southern Central Hospital of Yunnan Province, Honghe, China; 10 Department of Neurosurgery, Zhejiang Hospital, Hangzhou, China; 11 Department of Neurosurgery, Sir Run Run Shaw Hospital, Hangzhou, China; 12 Department of Neurosurgery, Nanjing Drum Tower Hospital, Affiliated Hospital of Medical School, Nanjing University, Nanjing, China; 13 Department of Neurosurgery, Qilu Hospital of Shandong University Dezhou Hospital, Dezhou, China; 14 Department of Neurology, Zhongshan Hospital, Fudan University, Shanghai, China; 15 Department of Neurosurgery, The Second People’s Hospital of Dongying, Dongying, China; 16 Caidya (formerly dMed Biopharmaceutical Co Ltd), Shanghai, China; 17 Institute for Science and Technology for Brain-inspired Intelligence, Fudan University, Shanghai, China; 18 The George Institute for Global Health, University of New South Wales, Sydney, Australia; 19 Neurology Department, Royal Prince Alfred Hospital, Sydney, Australia; 20 Department of Neurosurgery, National Regional Medical Center, Binhai Campus of the First Affiliated Hospital, Fujian Medical University, Fuzhou, China

## Abstract

**Importance:**

Transradial access (TRA) has emerged as a promising alternative to standard transfemoral access (TFA) for interventional cardiac procedures, but its application for examination of the cerebral circulation has not been tested in a clinical trial.

**Objective:**

To compare the efficacy and safety of TRA with TFA for diagnostic cerebral angiography.

**Design, Setting, and Participants:**

This investigator-initiated, multicenter, open-label, noninferiority randomized clinical trial with a blinded outcome assessment was conducted at 13 sites in China. Patients eligible for cerebral angiography were randomized between September 15, 2023, and November 4, 2024, with final follow-up performed on November 27, 2024. The primary analysis was performed in the intention-to-treat population; secondary analyses were performed in the per-protocol population.

**Interventions:**

Patients were randomly allocated to TRA (n = 431) or TFA (n = 430) for diagnostic cerebral angiography.

**Main Outcomes and Measures:**

The primary outcome was the success of diagnostic cerebral angiography. Secondary outcomes were success in achieving an accurate diagnosis, duration of angiography and fluoroscopy, time in bed, and patient-reported satisfaction on an 11-point visual analog scale for pain (ranging from 0 [none] to 10 [worst possible]) within 24 hours after the procedure. The noninferiority margin was an absolute difference of 5% in success of angiographic diagnosis and success of accurate diagnosis.

**Results:**

A total of 858 patients (median age, 58.4 [IQR, 52.0-67.0] years; 479 [55.8%] male) completed the trial. Success of diagnostic cerebral angiography in the TRA group was lower than that in the TFA group (392 of 431 [91.0%] vs 409 of 427 [95.8%]; difference, −4.8 percentage points [pp] [95% CI, −8.1 to −1.5 pp]; relative risk [RR], 0.95 [95% CI, 0.92-0.98]; *P* = .46 for noninferiority test). The success rate of accurate diagnosis was 78.9% in the TRA group vs 91.1% in the TFA group (difference, −12.2 pp [95% CI, −16.9 to −7.5 pp]; RR, 0.87 [95% CI, 0.82-0.92]; *P* = .99 for noninferiority test). Compared with the TFA group, the TRA group had longer median times for angiography (33.7 [IQR, 23.0-40.0] vs 38.7 [IQR, 26.0-47.0] minutes; *P* < .001) and fluoroscopy (10.6 [IQR, 5.6-12.9] vs 11.8 [IQR, 6.2-15.0] minutes; *P* = .02); the TRA group had significantly shorter median time in bed (188.4 [IQR, 3.0-180.0] vs 1079.0 [IQR, 842.0-1366.0] minutes; *P* < .001) and lower median pain scores (0.5 [IQR, 0.0-1.0] vs 0.7 [IQR, 0.0-1.0]; *P* < .001). Overall angiography complications were comparable between the groups (19 of 445 [4.3%] vs 25 of 413 [6.1%]; *P* = .28), but TRA had more radial artery puncture failures than TFA.

**Conclusions and Relevance:**

In this randomized clinical trial of patients undergoing diagnostic cerebral angiography, TRA was not shown to be noninferior to TFA with regard to the success rate of diagnostic cerebral angiography. Additional research, including superiority trials, is needed to clearly define the comparative benefits of TRA and TFA.

**Trial Registration:**

ClinicalTrials.gov Identifier: NCT05401669

## Key Points

**Question:**

Is transradial access (TRA) noninferior to transfemoral access (TFA) in efficacy and safety for diagnostic cerebral angiography?

**Findings:**

In this randomized clinical trial including 858 patients, the success rate for accuracy in diagnostic cerebral angiography was lower in the TRA group (91%) compared with the TFA group (96%) and did not meet the prespecified noninferiority margin.

**Meaning:**

TRA was not noninferior to TFA for diagnostic cerebral angiography.

## Teaser

This randomized clinical trial compares the safety and efficacy of transradial access with that of transfemoral access for patients undergoing diagnostic cerebral angiography.

## Introduction

Cerebral angiography is regarded as the gold standard for diagnosing various cerebral conditions. Although transfemoral access (TFA) remains the conventional standard owing to its procedural accessibility and well-established techniques, it carries a significant risk of complications, including retroperitoneal hematoma, pseudoaneurysm, arteriovenous fistula, femoral nerve injury, peripheral arterial occlusion, and access-site infection.^1,2,3^ Moreover, prolonged immobilization to access the femoral artery causes discomfort and increases the risk of venous thromboembolism in patients. Transradial access (TRA) has therefore gained in popularity as a viable alternative to TFA for interventional procedures, offering the potential advantages of lower risks of bleeding complications and access-site infection, faster recovery, and enhanced patient comfort.^3,4,5^ Observational studies have suggested that TRA is more effective and safer than TFA for cerebral angiography in relation to procedural success, complication rates, and patient satisfaction.^6,7,8,9^ However, in the absence of randomized evidence, TRA continues to be considered a secondary option, primarily used when TFA is unsuitable or presents technical difficulties, which limits its broader implementation. Accordingly, we undertook the TRA vs TFA for Cerebral Angiography (TRACE) study, a multicenter, randomized, open-label clinical trial to compare the safety and efficacy of TRA vs TFA for diagnostic cerebral angiography.

## Methods

### Study Design

TRACE was an investigator-initiated, multicenter, open-label randomized noninferiority trial with a blinded outcome assessment conducted at 13 hospital sites in China. The trial protocol and statistical analysis plan are provided in Supplement 1 and are described elsewhere.^10^ An overview of the study is given in eFigure 1 in Supplement 2. The study was approved by the ethics committee of Huashan Hospital, Fudan University, Shanghai, and the ethics committee at each participating hospital. The trial was overseen by an independent data and safety monitoring board. All participants or their legal representatives provided written informed consent. The trial followed the Consolidated Standards of Reporting Trials (CONSORT) reporting guideline.

### Participants

Patients were eligible for inclusion if they were aged 18 to 80 years, were independent in their daily activities (defined by a score of 0-2 on the modified Rankin Scale), and required diagnostic cerebral angiography that was suitable via either TRA or TFA access, where the diameter of the radial artery was 2 mm or greater on ultrasonography. Further details of the selection criteria are provided in eMethods in Supplement 2.

### Randomization, Blinding, and Masking

After screening procedures, patients were randomized in a 1:1 ratio via a computerized central system to receive TRA (intervention) or TFA (control). The permuted block randomization was used for the randomization procedure with the only stratification factor of study site. As this is an open-label trial, both patients and investigators were aware of the treatment allocation. However, blinding was applied to the outcome assessors, where the primary and secondary outcomes were determined by experts in a central imaging laboratory and clinical event committee without details of the treatment-group assignment.

### Study Intervention

Patients allocated to TFA underwent standard cerebrovascular angiography with imaging of the aortic arch and superselective angiography of its branch arteries, including bilateral internal carotid arteries, common carotid arteries, and vertebral arteries, to ensure an accurate diagnosis. A minimum of 50 prior TRA procedures was mandatory for all participating interventionalists to ensure operator competency and procedural standardization. Patients allocated to TRA underwent identical procedural requirements. All patients were followed up for 30 days.

### Outcomes

The primary outcome was success of diagnostic cerebral angiography, defined as the selection of the aortic arch vessel without changing the arterial access route and for the angiography to meet diagnostic criteria. Failure of the diagnostic procedure was any change in the arterial approach before or during the procedure. Secondary outcomes were success in providing an accurate diagnosis, duration of angiography, duration of fluoroscopy, time in bed, and pain on an 11-point visual analog scale (range, 0 [none] to 10 [worst possible]) within 24 hours after the procedure. A successful accurate diagnosis was defined as successful selective catheterization of the target vessel with an image quality score of good or very good, as defined in previous studies,^11^ and adjudicated by the blinded core laboratory. Safety outcomes were angiographic complications during and within 24 hours after the procedure, including access-related complications (ie, catheter kink or fracture, artery dissection, artery perforation, artery occlusion, compartment syndrome, arteriovenous fistula, retroperitoneal hematoma, hemorrhage, severe limb ischemia, embolism in any new territory, pseudoaneurysm, subcutaneous hematoma, and arterial spasm) and neurologic complications (eg, cerebral infarction, intracranial hemorrhage, cortical blindness, nerve injury, central nervous system infection, contrast encephalopathy, and vasovagal reactions such as hypotension, bradycardia, cold sweats, pallor, and clammy limbs). Complications that resulted in permanent sequelae, required hospitalization or extended hospitalization, necessitated surgery or other medical intervention, or led to death were classified as major complications; all other complications were classified as minor.

### Sample Size Calculation

Based on published data, success of diagnostic cerebral angiography via TRA and TFA ranges from 93.0% to 98.6% and 97.3% to 99.2%, respectively.^8,9,12,13,14^ Thus, we assumed success for diagnostic cerebral angiography via TRA and TFA would be 97% and 98%, respectively, with a noninferiority margin of −5%, a power of 90%, and a 1-sided significance level of *P* = .025. According to Power Analysis & Sample Size software, version 15 (NCSS Statistical Software), a sample size of 858 patients (429 per group) was required on the assumption of a 10% dropout rate.

### Statistical Analysis

The primary analysis was performed in the intention-to-treat population, with secondary analyses performed in the per-protocol population. The study protocol established a noninferiority margin of −0.05 (indicating an absolute difference of <5%) for both the primary outcome and secondary outcomes (success rate of accurate diagnosis). Noninferiority would be established if the lower bound of the 2-sided 95% CI around the difference in proportions of patients who achieved the primary outcome was greater than the predefined noninferiority margin. The Wald method was used to calculate 95% CIs for the rate of successful diagnosis and differences in rates between the 2 groups.^15^ Generalized linear regression models with a log link function, with group as covariate, were used to calculate the relative risk (RR) for the rates of successful diagnosis between the 2 groups. Descriptive statistics were provided for the secondary outcomes, with linear regression models used with group assignment used as a covariate. Generalized linear regression was also used for an exploratory analysis of factors that influenced the success of cerebral angiography via the TRA approach. These factors included age, sex, height, and body mass index; history of diabetes, hypertension, hyperlipidemia, and smoking; type III aortic arch configuration; radial artery diameter and development; aortic arch and arch vessel development and tortuosity; and history of radial artery surgery.

One interim analysis was planned and undertaken for safety and efficacy after 430 patients had completed their 30-day assessment. As the conservative Haybittle-Peto method was used to control the overall type I error rate, the boundary was set at *P* = .001 for the 1-sided test and 1-sided *P* = .024 for the final analysis. There was no imputation of missing data. All analyses were undertaken using SAS, version 9.4 (SAS Institute Inc).

## Results

**Figure 1.**
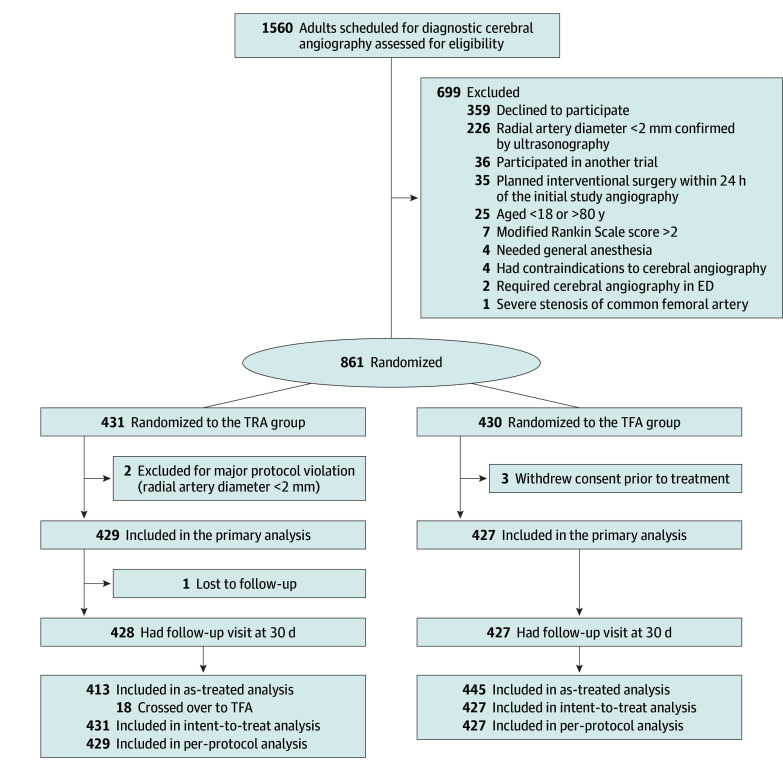
Participant Flow Diagram ED indicates emergency department; TFA, transfemoral access; and TRA, transradial access.

A total of 1560 patients scheduled for diagnostic cerebral angiography were screened at 13 sites in China between September 15, 2023, and November 4, 2024, of whom 861 were randomized: 431 to TRA and 430 to TFA. However, 3 patients, all in the TFA group, immediately withdrew their consent to participate in the study and were excluded from analysis. The remaining 858 patients (431 in the TRA group and 427 in the TFA group) were included in the final analysis. Two patients with protocol deviations in the TRA group were excluded from the per-protocol analysis, and 1 patient in the TRA group was lost to follow-up at 30 days after the procedure (Figure 1). Follow-up was completed November 27, 2024. The numbers of patients recruited by each site are outlined in eFigure 2 in Supplement 2. The median age of the participants was 58.4 (IQR, 52.0-67.0) years; 379 (44.2%) were female and 479 (55.8%) were male. Table 1 and eTable 1 in Supplement 2 show that the baseline characteristics were generally balanced between the groups.

**Table 1.** Baseline Demographic and Clinical Characteristics

Characteristic	Treatment group, No. (%) of participants	
	TRA (n = 431)	TFA (n = 427)
Age, median (IQR), y	59.1 (53.0-68.0)	57.8 (52.0-66.0)
Sex		
Male	244 (56.6)	235 (55.0)
Female	187 (43.4)	192 (45.0)
BMI, median (IQR)	24.4 (22.4-26.3)	24.8 (22.5-26.4)
Height, median (IQR), cm	165.2 (160.0-170.0)	164.9 (158.0-171.0)
Weight, median (IQR), kg	66.9 (60.0-75.0)	67.5 (60.0-75.0)
Medical history^a^		
Hypertension	255 (59.2)	236 (55.3)
Diabetes	66 (15.3)	56 (13.1)
Hyperlipidemia	63 (14.6)	54 (12.6)
Coronary heart disease	15 (3.5)	16 (3.7)
Smoking history	51 (11.8)	51(11.9)
Alcohol consumption history	38 (8.8)	43 (10.1)
Premorbid function on mRS score^b^		
0	337 (78.2)	341 (79.9)
1	76 (17.6)	70 (16.4)
2	17 (3.9)	15 (3.5)
3	1 (0.2)	1 (0.2)
Puncture history^c^	145 (33.6)	139 (32.6)
Left radial artery	1 (0.7)	2 (1.4)
Right radial artery	17 (11.7)	22 (15.8)
Left femoral artery	10 (6.9)	9 (6.5)
Right femoral artery	117 (80.7)	106 (76.3)
Mean artery diameter, median (IQR), mm		
Left radial	2.4 (2.1-2.6)	2.3 (2.1-2.5)
Right radial	2.4 (2.1-2.6)	2.4 (2.1-2.6)
Left femoral	7.8 (6.8-8.7)	7.8 (6.9-8.7)
Right femoral	7.9 (6.9-8.8)	7.9 (6.9-8.8)
Radial artery developmental abnormality^d^		
Left	0	NA
Right	10 (2.3)	NA
Aortic arch type^e^		
I plus II	306 (71.0)	305 (71.4)
III	87 (20.2)	84 (19.7)
Missing	38 (8.8)	38 (8.9)
Aortic arch variations^f^	44 (10.2)	37 (8.7)
Supra-aortic artery tortuosity^g^		
Normal	83 (19.3)	80 (18.7)
Tortuous	304 (70.5)	305 (71.4)
Kinking and looping	7 (1.6)	5 (1.2)
Missing	37 (8.6)	37 (8.7)

Abbreviations: BMI, body mass index (calculated as weight in kilograms divided by height in meters squared); mRS, modified Rankin Scale; NA not applicable.

^a^ Patient self-report or family report.

^b^ Scores range from 0 (no functional limitations) to 5 (severe disability). A score of 2 or less indicates functional independence.

^c^ Refers to any history of radial or femoral artery puncture.

^d^ Includes tortuosity, hypoplasia, radial-ulnar artery loop, high-origin radial artery, stenosis, and other variations.

^e^ Assessed by the imaging core laboratory. According to the Myla classification,^14^ the aortic arch is categorized into types I, II, and III based on the vertical distance from the apex of the aortic arch to the origin of the brachiocephalic trunk, using the diameter of the left common carotid artery as a reference. If the vertical distance is smaller than the diameter of the left common carotid artery, it is classified as type I; a vertical distance 1 to 2 times the diameter is classified as type II; and a distance greater than 2 times the diameter is classified as type III.

^f^ Data were missing for 38 patients in the TRA group and 38 patients in the TFA group.

^g^ Normal indicates that the artery has a straight course with no or minimal tortuosity; tortuous indicates that the artery exhibits an S- or C-shaped elongation or undulation along its course; and kinking and looping indicates that the artery shows an acute angulation (kinking) and demonstrates elongation or redundancy, forming an exaggerated S-shaped curve or a circular structure (looping).

The success rate of diagnostic cerebral angiography in the TRA group was lower than that in the TFA group (392 of 431 [91.0%] vs 409 of 427 [95.8%], respectively; difference, −4.8 percentage points [pp] [95% CI −8.1 to −1.5 pp]; RR, 0.95 [95% CI, 0.92-0.98]; *P* = .46 for noninferiority test) (Table 2). A total of 39 patients in the TRA group did not complete cerebral angiography, with 12 due to radial artery puncture failure, 7 due to a change in the arterial access route, and 20 due to failure to meet the established angiographic standards. In the TFA group, 18 patients did not complete cerebral angiography, as they failed to meet standard angiographic criteria. The per-protocol analysis produced similar results for the success rate of diagnostic cerebral angiography between the TRA and TFA groups (392 of 429 [91.4%] vs 409 of 427 [95.8%]; difference, −4.4 pp [95% CI, −7.7 to −1.1 pp]; RR, 0.95 [95% CI, 0.92-0.99]; *P* = .36 for noninferiority test). However, the as-treatment analysis showed no significant between-group differences in success rates (393 of 413 [95.2%] vs 423 of 445 [95.1%]; difference, 0.1 pp [95% CI, −2.8 to 3.0 pp]; RR, 1.00 [95% CI, 0.97-1.03]; *P* < .001 for noninferiority test) (eTables 2 and 3 in Supplement 2).

**Table 2.** Primary and Secondary Outcomes

Outcome	TRA group (n = 431)	TFA group (n = 427)	Difference (95% CI), pp^a^	Relative risk (95% CI)^b^	*P* value
Primary: diagnostic success, No. (%)	392 (91.0)	409 (95.8)	−4.8 (−8.1 to −1.5)	0.95 (0.92 to 0.98)	.46^c^
Failure stage of primary outcome, No. (%)^d^					
1	12 (2.8)	0	NA	NA	NA
2	4 (0.9)	0	NA	NA	NA
3	3 (0.7)	0	NA	NA	NA
4	20 (4.6)	18 (4.2)	NA	NA	NA
Secondary outcomes					
Success of accurate diagnosis, No. (%)	340 (78.9)	389 (91.1)	−12.2 (−16.9 to −7.5)	0.87 (0.82 to 0.92)	>.99^c^
Duration of angiography, median (IQR), min	38.7 (26.0 to 47.0)	33.7 (23.0 to 40.0)	4.9 (2.7 to 7.2)	NA	<.001^e^
Duration of fluoroscopy, median (IQR), min	11.8 (6.2 to 15.0)	10.6 (5.6 to 12.9)	1.3 (0.2 to 2.3)	NA	.02^e^
Time in bed, median (IQR), min	188.4 (3.0 to 180.0)	1079.0 (842.0 to 1366.0)	−890.6 (−998.4 to −792.9)	NA	<.001 ^e^
VAS score, median (IQR)^f^	0.5 (0.0 to 1.0)	0.7 (0.0 to 1.0)	−0.25 (−0.37 to −0.12)	NA	<.001 ^e^

Abbreviations: pp, percentage points; TFA, transfemoral access; TRA, transarterial access; VAS, visual analog scale for pain.

^a^ Indicates absolute incidence difference without adjustment.

^b^ Calculated as generalized linear regression models with log link function.

^c^ Calculated with the Wald test, noninferiority test with a noninferiority margin of −5%.

^d^ Stage 1 indicates an inability to insert the arterial sheath (eg, small artery diameter, no blood return after arterial puncture, inability to advance the guidewire due to vasospasm, atherosclerotic plaque). Stage 2 indicates that the puncture and sheath placement was successful but the catheter or guidewire could not be advanced (eg, arterial tortuosity, vasospasm, looping). Stage 3 indicates that the guidewire and catheter reached the aortic arch but the primary branch of the aortic arch could not be selected. Stage 4 indicates that the guidewire and catheter reached the primary branch of the aortic arch but the procedure still could not be completed successfully.

^e^ Calculated from the linear regression model, with group as covariate.

^f^ Scores range from 0 (no pain) to 10 (worst possible pain).

Results for the secondary outcomes are shown in Table 2 and eFigures 4 to 8 in Supplement 2. Success of accurate diagnosis occurred in 340 of 431 patients (78.9%) in the TRA group and 389 of 427 (91.1%) in the TFA group (difference, −12.2 pp [95% CI, −16.9 to −7.5 pp]; RR, 0.87 [95% CI, 0.82-0.92]; *P* > .99 for noninferiority test). The median duration of angiography was longer in the TRA group compared with the TFA group at 38.7 (IQR, 26.0-47.0) vs 33.7 (IQR, 23.0-40.0) minutes, respectively (difference, 4.92 [95% CI, 2.65-7.18] minutes; *P* < .001); similar results were observed for fluoroscopy at 11.8 (IQR, 6.2-15.0) vs 10.6 (IQR, 5.6-12.9) minutes, respectively (difference, 1.26 [95% CI, 0.19-2.32]; *P* = .02). However, the TRA group had a shorter median time in bed (188.4 [IQR, 3.0-180.0] vs 1079.0 [IQR, 842.0-1366.0] minutes; difference, −890.64 [95% CI, −998.42 to −792.86] minutes; *P* < .001) and a lower median VAS score for pain (0.5 [IQR, 0.0-1.0] vs 0.7 [IQR, 0.0-1.0]; difference, −0.25 [95% CI, −0.37 to −0.12]; *P* < .001).

**Figure 2.**
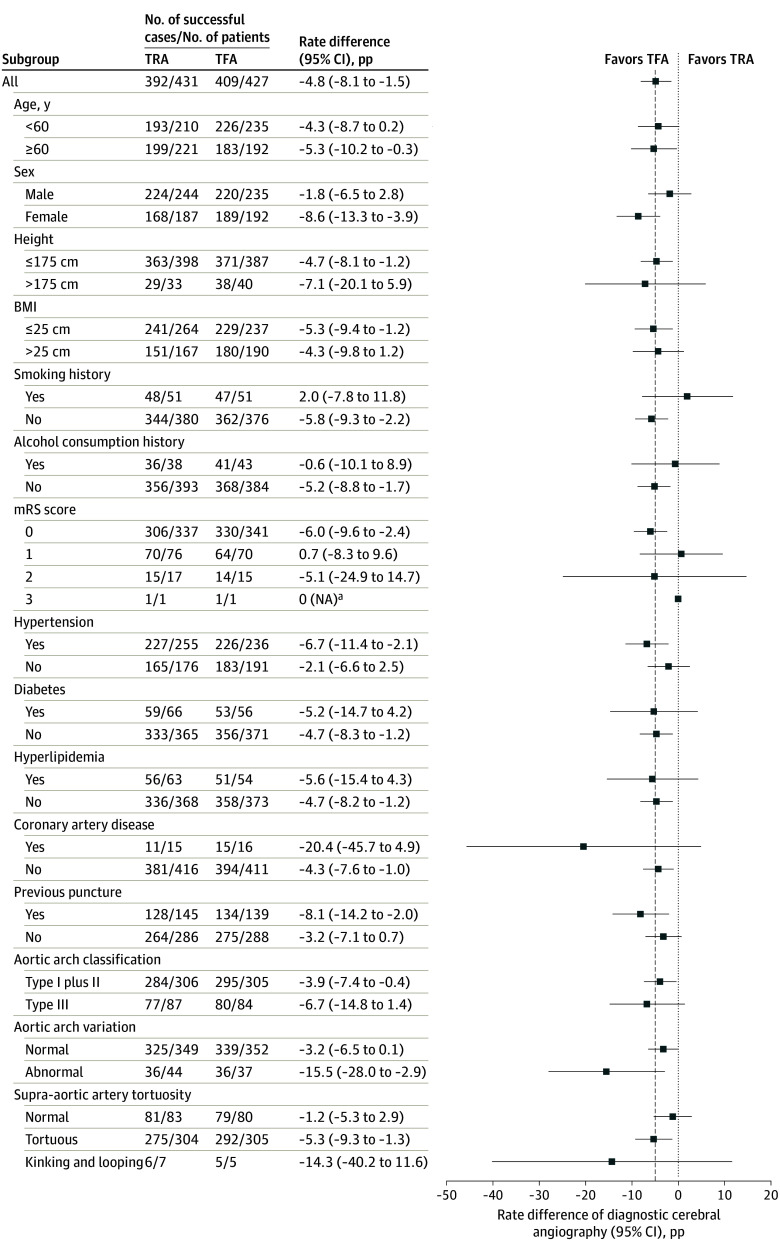
Heterogeneity of the Effect for the Primary Outcome Across the Prespecified Subgroup Modified Rankin Scale (mRS) scores range from 0 (no functional limitations) to 5 (severe disability); 2 or less indicates functional independence. The dashed vertical line indicates the noninferiority margin. BMI indicates body mass index (calculated as weight in kilograms divided by height in meters squared); NA, not applicable; pp, percentage points; TFA, transfemoral access; and TRA, transradial access. ^a^Due to the small number of events (1 per group), the sample size was insufficient to reliably estimate the standard error; therefore, a valid 95% CI could not be calculated.

The safety outcomes are shown in eTable 4 in Supplement 2. There were no significant differences in angiographic complications within 24 hours of the procedures (25 of 413 [6.1%] for TRA vs 19 of 445 [4.3%] for TFA; *P* = .28). In particular, access-related complications occurred in 23 of 413 patients (5.6%) in the TRA group and 17 of 445 (3.8%) in the TFA group; and neurologic complications, 2 of 413 (0.5%) in the TRA group and 3 of 445 (0.7%) in the TFA group. However, the rate of artery occlusion was higher in TRA group compared with the TFA group (20 of 413 [4.8%] vs 1 of 445 [0.2%]), while the rates of pseudoaneurysm (1 of 413 [0.2%] vs 4 of 445 [0.9%]) and subcutaneous hematoma (2 of 413 [0.5%] vs 8 of 445 [1.8%]) were lower in the TRA group compared with the TFA group. Two major angiographic complications (1 ischemic stroke and 1 femoral artery pseudoaneurysm) occurred within 24 hours of TFA. There were no deaths. There was no clear heterogeneity of the effect across the prespecified subgroups, but the overall trend was in favor of TFA in both the main (Figure 2 and eTable 5 in Supplement 2) and per protocol analyses (eTable 6 and eFigures 3 to 8 in Supplement 2).

## Discussion

This multicenter randomized clinical trial conducted in China indicates that compared with standard TFA, TRA is associated with a lower rate of successful diagnostic cerebral angiography and longer procedural times. However, TRA produced a shorter time in bed and was more desirable from a pain perspective for patients than TFA. These differences could be largely explained by greater access issues and greater failures in access of TRA compared with TFA.

TRA has emerged as the preferred approach for interventional cardiologists, with several clinical trials^3,4,5,16^ showing that it reduces the risks of vascular bleeding, kidney complications, and death, and it is preferred by patients. The TRA approach was introduced into cerebral angiography and neurointervention in 2020,^17^ and subsequent observational studies^12,13,18,19,20^ have shown it to have efficacy and safety comparable to that of TFA. However, our randomized comparison has shown that the success rate for diagnostic angiography is lower than that of TFA, primarily because of greater failures with radial artery puncture. This may have been the result of poor procedural skills of the interventionalists for TRA in our study, as there is a steep learning curve to achieve competency. In the 39 cases of angiography failure in the TRA group, this was due to puncture failure in 12 compared with no similar cases in the TFA group. However, if radial artery puncture is successfully achieved, the success of TRA appears comparable to that of TFA.

Our definition of successful diagnostic cerebral angiography not only required selective catheterization of the aortic arch vessels but also mandated that the image quality be sufficient for a definitive diagnosis. Thus, the greater difficulty in achieving successful cannulation via TRA, particularly for the left vertebral artery,^9,20,21^ may have resulted in an inferior image quality being achieved, which in turn led to lower success of diagnosis compared with TFA. It is important to note, however, that an accurate diagnosis requires successful selective catheterization of the target vessel, for which TRA was found to be inferior to TFA, as noted in other studies.^9,14,20,21,22^ Among the subgroup of patients with normal angulation of the supra-aortic artery, there appeared to be similar diagnostic success between the groups, which is again consistent with another study.^23^ Thus, for patients with tortuous supra-aortic vessels, TFA may be a better option at present until technological advances improve the success of TRA for neurointerventional procedures.

The prolonged procedural and fluoroscopy times in the TRA group are consistent with those of other studies.^9,14^ However, the requirement for a longer compression time for TFA allowed patients to be mobilized earlier after TRA, and patients in this group reported greater comfort from expressing less pain. Other studies^3,9,14,19,24^ report a lower complication rate for TRA compared with TFA, particularly of puncture site–related complications. Our study found no significant difference in the overall angiography complication rates between TRA and TFA, although with the former had lower rates of puncture site bleeding and pseudoaneurysms. The higher complication rate in the TRA group was primarily attributed to radial artery occlusion in 20 patients (4.8%), which is comparable with previous studies.^25,26,27^ However, radial artery occlusion often goes unnoticed, as it is usually asymptomatic. The requirement for an ultrasonographic examination within 24 hours of TRA in our study probably increased the identification of occlusions, which in turn led to comparable between-group complication rates. Again, the success of TRA may be increased by an ability to reduce radial artery occlusion rates through better techniques to improve patent hemostasis and distal radial puncture.^28,29,30^

### Limitations

Despite being a well-powered multicenter randomized clinical trial with objective and blinded assessment of the imaging measures, we acknowledge that our study has several limitations. First, the open-label design could have led to observer and responder bias, particularly in the measurement of pain, which is subjective. Additionally, there may be concerns over generalizability, as the study population was limited to patients in China. Although the study required participating investigators to have had experience in performing at least 50 TRA for competency and standardization to be ensured, TRA remains a challenging procedure to achieve peak efficiency.^21,31,32^ This issue may also mean that the difference in the success of TRA would be reduced in the hands of less experienced operators and centers. We have not undertaken an economic analysis or an assessment of broader measures of patient outcomes, including health-related quality of life or functional recovery, which are critical factors in determining implementation and scalability of TRA in practice. In addition, the scope of the noninferiority test and noninferiority margin were only designed for the interpretation of the primary outcome and the secondary outcome of success in providing an accurate diagnosis. Therefore, they are not applicable and should not be extended to infer noninferiority for any other end points. The power and sample size calculations were only for the primary end point. Thus, findings of secondary outcomes may be underpowered in the present study. Further confirmatory studies will be needed for drawing formal conclusions. Finally, as our study focused on diagnostic cerebral angiography, the findings may not be applicable to other angiographic procedures with differing complexity and risks.

## Conclusions

Our randomized clinical trial showed that TRA was not noninferior to TFA with regard to the success rate of diagnostic cerebral angiography. Further research, including superiority trials, is needed to more clearly define the comparative benefits of TRA and TFA for cerebrovascular angiography.

## Notes

Supplement 1.
Trial Protocol and Statistical Analysis Plan

Supplement 2. **eMethods.** Follow-up Schedule and Inclusion and Exclusion Criteria **eFigure 1.** Overview of the TRACE Trial **eFigure 2.** Number of Patients Recruited by Each Center **eFigure 3.** Subgroup Analysis of Primary Outcome in Per-Protocol Analysis **eFigure 4.** Subgroup Analysis of Secondary Outcome (Success Rate of Accurate Diagnosis) **eFigure 5.** Subgroup Analysis of Secondary Outcome (Duration of Angiography) **eFigure 6.** Subgroup Analysis of Secondary Outcome (Duration of Fluoroscopy) **eFigure 7.** Subgroup Analysis of Secondary Outcome (Bedridden Time) **eFigure 8.** Subgroup Analysis of Secondary Outcome (VAS Score) **eTable 1.** Additional Baseline and Procedural Characteristics **eTable 2.** Primary and Secondary Outcomes in Per-Protocol Analysis **eTable 3.** Primary and Secondary Outcomes in As-Treated Analysis **eTable 4.** Safety Outcomes Assessed by the CEC-ITT **eTable 5.** Subgroup Analysis of Primary Outcome in Intention-to-Treat Analysis **eTable 6.** Subgroup Analysis of Primary Outcome in Per-Protocol Analysis
eReferences

Supplement 3.
Nonauthor Collaborators

Supplement 4.
Data Sharing Statement
