# Supplementary material for: Transradial vs Transfemoral Access for Cerebral Angiography: A Randomized Noninferiority Clinical Trial
Source: JAMA Netw Open. 2026 Mar 19;9(3):e261929. doi: 10.1001/jamanetworkopen.2026.1929 (PMC13003373; doi:10.1001/jamanetworkopen.2026.1929)
Supplement: Supplement 1. — Trial Protocol and Statistical Analysis Plan [file jamanetwopen-e261929-s001.pdf]

TransRadial versus transfemoral Arterial access for CErebral angiography (TRACE)

(A prospective, multicenter, randomized, controlled clinical study)

Study Number:	TRACE
Protocol Version:	Version 1.0
Version Date:	Feb 27, 2023
Coordinating Investigator:	Yuxiang Gu
Sponsor:	Huashan Hospital of Fudan University

Protocol Signature Page

Sponsor:	Signature	Date
Huashan Hospital of Fudan University		

I have thoroughly read this protocol and agree that it covers all the necessary information to conduct the clinical trial. I will carry out this study according to the study protocol and complete the study within the planned deadline.

I will provide copies of this protocol and all relevant materials to all personnel assisting me in conducting this study. I will discuss the materials with them to ensure that they fully understand the trial protocol and how to conduct this trial.

Name	Signature	Date
[Principal Investigator]:		

Protocol Synopsis

**Study Title**	TransRadial versus transfemoral Arterial access for CErebral angiography (TRACE)<br>(A prospective, multicenter, randomized, controlled clinical study)
**Primary Study Objectives**	To demonstrate that the success rate of diagnostic cerebral angiography of transradial arterial access is not inferior to that of transfemoral arterial access
**Study Design**	This is a prospective, multicenter, randomized, controlled, non-inferiority study
**Number of Planned Sites**	About 13 study sites in China
**Statistical Considerations and Sample Size**	This study will test the hypothesis that the success rate of diagnostic cerebral angiography by transradial arterial access is not inferior to that by transfemoral arterial access. We assume the success rates of diagnostic cerebral angiography by transradial arterial access and transfemoral arterial access are 97% and 98% respectively by considering the data in the references, with a margin for non-inferiority of -5%, a power of 90% at one-side significance level of 0.025, and a drop-out rate of 10%, 429 cases are needed for each group according to PASS 15 software, and thus a total of 858 subjects need to be enrolled in this study.
**Inclusion Criteria**	1. Age between 18 and 80 years old;<br>2. Patients who are scheduled to receive diagnostic cerebral angiography;<br>3. Patients who are suitable for cerebral angiography through transradial arterial access and transfemoral arterial access as indicated by objective evidence, with radial artery diameter ≥2mm based on ultrasonography;<br>4. Modified Rankin Scale (mRS) score ≤2;

	5. Patients who have provided written informed consent form.
**Exclusion Criteria**	1. Severe stenosis of radial artery, brachial artery, subclavian artery, brachiocephalic artery, iliac artery or common femoral artery as found in imaging examination, or any serious vascular disease (such as aortic aneurysm, etc.) along the path of the angiographic catheter, which may hinder the passage of a guidewire;<br>2. Arteriovenous fistula for hemodialysis present in the right upper limb;<br>3. Any planned interventional surgery within 24 hours of the first study angiography;<br>4. Require emergency cerebral angiography;<br>5. General anesthesia is used;<br>6. Any contraindication to cerebral angiography, such as allergy or intolerance to the contrast media, uncorrected severe coagulation disorders, arterial dissection in the target vessel, puncture site infection, and renal insufficiency (Creatinine [Gr] > 3 times of upper limit of normal [ULN]), etc.;<br>7. Women who are pregnant or plan to become pregnant within 1 year;<br>8. Patients who are participating in any other clinical trial;<br>9. Any other condition which is considered by the investigator as unsuitable for participating in this study.
**Study Intervention**	After being screened according to the inclusion and exclusion criteria, patients will be randomized to the test group (cerebral angiography via transradial arterial access) and the control group (cerebral angiography via transfemoral arterial access) at the ratio of 1:1 through a computerized central randomization system.<br>Please see the SOP for the specific requirements of cerebral angiography by transradial and transfemoral arterial access.

**Primary Effectiveness Endpoints**	The success rate of diagnostic cerebral angiography;<br>Successful diagnostic angiography: the aortic arch vessel is successfully selected without changing the puncture site, with angiography results meeting the diagnostic requirements.
**Secondary Effectiveness Endpoints**	1. Success rate of accurate diagnosis<br>2. Duration of angiography<br>3. Duration of fluoroscopy<br>4. Bedridden time<br>5. Visual Analogue Scale (VAS) score (within 24h)
**Safety Endpoints**	1. Incidences of angiographic complications during and within 24 hours after the procedure<br>2. Incidences of major angiographic complications during and within 24 hours after the procedure<br>Angiographic complications during and within 24 hours after the procedure may include:<br>☐ Access route associated complications (catheter kink or fracture, artery dissection, artery perforation, artery occlusion, compartment syndrome, arteriovenous fistula, retroperitoneal hematoma, hemorrhage, severe limb ischemia, embolism in any new territory, pseudoaneurysm, subcutaneous hematoma, and arterial spasm, etc.). Any complication which results in permanent sequelae, requires hospitalization, prolongation of existing hospitalization, any surgery or other medical intervention, or leads to death will be considered as a **major** complication, while all other complications will be considered as **minor** complications.<br>☐ Neurological complications (cerebral infarction, intracranial hemorrhage, cortical blindness, nerve injury, nervous system infection, contrast encephalopathy, and vasovagal reactions

	including decreased blood pressure, decreased heart rate, cold sweat, pale, clammy limbs, etc.). Any complication which results in permanent sequelae, requires hospitalization, prolongation of existing hospitalization, any surgery or other medical intervention, or leads to death will be considered as a **major** complication, while all other complications will be considered as **minor** complications.

Table of Contents

Protocol Synopsis......1
List of Abbreviations......7
1. Introduction......9
2. Study Objectives and Endpoints......13
2.1 Study Objectives:......13
2.2 Study Endpoints:......13
3. Study Design......15
3.1 Overall Study Design:......15
3.2 Study Contents:......15
3.3 Study Flow Diagram:......16
4. Study Population:......16
4.1 Inclusion Criteria (only subjects who meet all of the following criteria can be considered to enter into this study)......16
4.2 Exclusion Criteria (Subjects who meet any of the following criteria are not allowed to participate in this study)......17
4.3 Criteria and Procedures for Subject Withdrawal......17
5. Study Interventions......18
5.1 Standard Operating Procedures for TRA and TRF Cerebral Angiography......18
5.2 Restrictions......22
6. Study Procedures......22
6.1 Study Procedures......22
6.2 Informed Consent and Subject Enrollment......22
6.3 Randomization......23
6.4 Clinical Investigations and Assessments......23
7. Statistical Analysis......24
7.1 General Principles......24
7.2 Hypothesis Testing......24
7.3 Sample Size Estimation......24
7.4 Datasets for Analysis......24
7.5 Handling of Missing Data......25
7.6 Safety Analysis......25
7.7 Effectiveness Analysis......26
8. Data Management......26
8.1 Completion of Case Report Form......27
8.2 Data Verification and Query......27
8.3 Lock of the Database......27
9. Ethical Protection and Informed Consent......27
9.1 Ethical Considerations, Whether Involving Human Genetic Resources and Use of Highly Pathogenic Microorganisms......27
9.2 Study Protocol Approval......28
9.3 Informed Consent Process and Informed Consent Form......28
10. Stipulation on Adverse Events......28
10.1 Adverse Events......28

10.2 Serious Adverse Events........29
10.3 Possible Adverse Events........29
10.4 Determination of Relationship Between Adverse Events and the Angiography 33
10.5 Reporting and Treatment of Serious Adverse Events........33
11. Administration Considerations........34
11.1 Confidentiality of the Subjects........34
11.2 Study Monitoring........34
11.3 Case Report Form and Study Records........34
11.4 Financial Disclosure........35
11.5 Data and Safety Monitoring Board........35
11.6 Clinical Event Committee........36
11.7 Independent Imaging Assessment Committee........36
12. Confidentiality Principles........36
13. Agreement on Publication of Study Results........36
14. References........38
Appendix 1 Study Flow Chart........40
Appendix 2 Modified Rankin Scale (mRS)........42
Appendix 3 Visual Analogue Scale (VAS)........43

List of Abbreviations

ALT	Alanine aminotransferase
APTT	Activated Partial Thromboplastin Time
AST	Aspartate aminotransferase
BP	Blood Pressure
BUN	Blood Urea Nitrogen
CEC	Clinical Event Committee
Cr	Creatinine
CRC	Clinical Research Coordinator
CRF	Case Report Form
CRO	Clinical Research Organization
CT	Computed Tomography
CTA	Computerized Tomographic Angiography
DBP	Diastolic Blood Pressure
DSA	Digital Subtraction Angiography
DSMB	Data and Safety Monitoring Board
EC	Ethics Committee
eCRF	Electronic Case Report Form
EDC	Electronic Data Capture System
g/L	Grams per Litre
GCP	Good Clinical Practice
IA	Intra-arterial
IC	Informed Consent
ICA	Internal Carotid Artery
ICH	Intracranial Hemorrhage
INR	International Normalized Ratio
IRB	Institutional Review Board
IV	Intravenous
MMA	Middle Meningeal Artery
MRI	Magnetic Resonance Imaging
mRS	Modified Rankin Scale
OAC	Outcome Assessment Committee
PI	Principal Investigator
RCT	Randomized Controlled Trial
REB	Research Ethics Board

SAE	Serious Adverse Event
SAP	Statistical Analysis Plan
SOP	Standard Operating Procedure
TEAE	Treatment Emergent Adverse Event
TFA	Transfemoral Access
TFCA	Transfemoral Cerebral Angiography
TRA	Transradial Access

Main Body Part of the Protocol

1. Introduction

With the development of economics and the improvement of living standards, the prevalence of cardiovascular and cerebrovascular diseases is increasing annually. Cardiovascular and cerebrovascular disease is the leading cause of death in China[1]. The mortality rate of cerebrovascular diseases in rural areas was 157.48/100,000, and that in urban areas was 126.58/100,000 in 2017. In China, there are about 2.7 million new cases of stroke every year, and the rate is increasing by 8.7% continuously. Therefore, the prevention and treatment of cerebrovascular diseases has significant impact on improving human health and reducing social burdens. As the gold standard in the diagnosis of cerebrovascular diseases, cerebral angiography greatly improves the detection rate of cerebrovascular diseases and plays an important role in the prevention and treatment of cerebrovascular diseases.

The conventional surgical approach for cerebrovascular angiography is via transfemoral access (TFA), which is widely used in angiographic diagnosis and interventional treatment of cerebrovascular diseases. Although having been used for decades, transfemoral cerebral angiography (TFCA) is found to have many disadvantages in clinical practice. First of all, expensive percutaneous vascular closure devices are needed after the procedure, to suture the vascular puncture wound or for hemostasis by compression. Besides, 12-24 hours of lower limb immobilization and on-bed rest are needed, which not only brings a lot of inconveniences and pain to the patients, but may also induce lower limb thrombosis, which may lead to pulmonary embolism and endanger the life of the patients. Secondly, as the femoral artery is a deep artery, accompanied by important nerves and blood vessels around it, puncture for TFCA has a high rate of complications such as retroperitoneal hematoma, pseudoaneurysm formation, arteriovenous fistula formation, femoral nerve injury, and lower limb ischemia, etc., which cannot be completely avoided. In addition, elder patients have higher rate of atherosclerotic cerebrovascular and coronary artery disease, and therefore anticoagulant or antiplatelet therapies are more common used. These

therapies can increase the risk of complications from hemorrhage due to puncture for femoral access. Finally, TFCA is not feasible for patients with extensive atherosclerotic disease in the aortic arch, atypical anatomical structure of the aorta or brachial vessels, thoracic artery dissection, iliofemoral occlusive disease, or groin infection.

With the continuous development of endovascular intervention therapy, transradial access (TRA) has gradually become a new arterial approach. Transradial coronary angiography was first reported by Campau in 1989 [2]. It has subsequently been widely used in interventional treatment of cardiovascular diseases, and has gradually replaced the femoral approach as the preferred artery access for coronary angiography and treatment. Numerous clinical studies indicate that the cost of hospitalization via radial artery approach is significantly reduced, with shorter length of hospitalization, higher patients' acceptance and satisfaction, and less complications, comparing with coronary angiography and interventional treatment via femoral artery access[3]. Subsequently, TRA has been gradually adopted to angiography and interventional treatment of cerebrovascular diseases, and has been preferred by neuro-interventional surgeons and patients. Compared with TFA, TRA has the advantages of less damage, easier hemostasis, quicker recovery, lower rate of puncture related complications, and shorter flat time. However, due to the anatomical differences, angiography and interventional treatment via the radial approach are more challenging than the femoral approach, resulting in slower development. In recent years, continuous progress in angiography techniques and interventional devices have led to an increasing number of neuro-interventional surgeons choosing radial artery approach for cerebrovascular angiography and interventional treatment. Studies have shown that there is no statistical difference in the success rate of angiographic diagnosis between the femoral and radial approaches [4, 5]. Besides, TRA offers a range of benefits over the traditional femoral approach, including significantly lower complication rates, shorter hospital stays, and greater patient comfort and satisfaction[5]. However, TRA cerebral angiography and interventional treatment also has its difficulties and short comings. First of all, the success rate of radial artery puncture is significantly lower than that of femoral artery puncture due to the short development time of transradial artery puncture and the

thinner radial artery. Therefore successful radial artery puncture which requires continuous experience accumulation. Secondly, transradial cerebral angiography currently utilizes the Simon2 catheter, which requires the formation of a loop after entering the aortic arch to superselect the target vessel. This looping technique represents a critical step in transradial angiography. While in patients with Type III aortic arch configuration, a loop has to be formed into in the aortic valve as the catheter cannot be advanced into the descending aorta through the brachiocephalic trunk. This technique has a high risk as the friction of the guidewire and the catheter on the aortic valve may cause attachment on the valve orifice to fall off, resulting in embolism. Besides, the guidewire and catheter entering the heart cavity through the valve opening may result in arrhythmia, detachment of mural thrombus in the cardiac cavity, and even cardiac perforation[6]. Finally, due to anatomical differences, it is more difficult to push the angiographic catheter to the target position during selective cerebral angiography via TFA compared with TRA, and the success rate of superselection of secondary aortic arch vessels is relatively low. Therefore, study has found that the puncture time, procedure time and fluoroscopy time of cerebral angiography via TRA are significantly higher than those via TFA[7]; However, some studies have shown that with the accumulation of experience and improvement of techniques, the procedure time and fluoroscopy time of cerebral angiography via TRA approach can be significantly reduced, and less procedure time is needed for TRA by experienced neuro-interventionists compared with TFA[8]; Especially for patients with vascular variations such as type II-III aortic arch configuration and bovine arch, cerebral angiography via TRA can greatly reduce the difficulty in manipulation as well as the procedure time and fluoroscopy time[9]. Besides, the TRA approach can offer more direct navigation into the posterior circulation than the TRA approach thanks to the anatomical features that the vertebral artery originates from the subclavian artery, and thus the TRA has advantages over TFA in diagnosis and treatment of posterior circulatory lesions[10]. In conclusion, the TRA approach has a very broad application potential and great significance in angiography and interventional treatment of cerebrovascular diseases, which may be the trend in future neuro-interventional development. However, due to

lack of high-quality clinical study results which can provide evidence-based medical data, the TFA approach is still the preferred arterial access for cerebrovascular angiography and interventional treatment at present, while TRA approach is often used as an alternative arterial route which has not been widely used so far.

Relevant techniques and materials are still in rapid development stage due to the late start of TRA in cerebral angiography. At present, many patents on the manufacture of TRA cerebral angiography catheters have been applied in China. However, due to lack of standard clinical trial verification, the industrialization of these patents is difficult. Therefore carrying out clinical studies on cerebral angiography with TRA approach are absolutely necessary. Clinical trial is an important approach in promoting the development of TRA cerebral angiography technology. High-quality clinical trials of TRA cerebral angiography can not only accelerate the development of TRA technology and materials, but also contribute to the industrialization of relevant technologies and product patents, and it also plays a key role in promoting the development of social economy.

In summary, compared with TFA, TRA has the advantages of less complications, faster postoperative recovery, shorter flat time, no need of exposure patient’s privacy part, and shorter hospital stay time and lower cost. However, the operations of radial artery puncture and superselection of angiographic catheter are more complicated and difficult, requiring higher technical skills and resulting in a steeper learning curve, thus leading to relatively longer procedure time and fluoroscopy time. However, with the development of neurointerventional techniques and materials, the technical difficulty of TRA is gradually reduced, and the procedure time will be further shortened. In conclusion, the TRA approach has a very broad application potential in cerebral angiography and interventional treatment. We plan to collaborate with 12 sites in China with rich experience in TRA arteriography to conduct a multicenter, randomized controlled study comparing the effectiveness and safety of TRA and TRF for cerebral angiography and neuro-interventional treatment, to provide high-level evidence-based medical data on the application of TRA in the field of neuro-interventional treatment, thus promoting the application of TRA approach in cerebral angiography and

interventional treatment, so as to improve treatment, reduce medical costs, and accelerate the development of neuro-intervention.

2. Study Objectives and Endpoints

2.1 Study Objectives:

(1) Primary objectives:

To demonstrate that the success rate of diagnostic cerebral angiography via TRA is not inferior to that via TFA

(2) Secondary objectives:

To analyze factors affecting cerebral angiography via TRA approach.

2.2 Study Endpoints:

(1) Effectiveness endpoints in this study include:

- **Primary Effectiveness Endpoints**

Success rate of diagnostic cerebral angiography

Successful diagnostic angiography: the aortic arch vessel is successfully selected without changing the puncture site, with angiography results meeting the diagnostic requirements.

For intraoperative management, please refers to *Chinese Expert Consensus on Good Practice for Cerebral Angiography (2018)*.

In case of angiography failure, record in which of the following stages it occurs and why it occurs:

Stage 1: Unable to insert the arterial sheath (small artery diameter, no blood return after arterial puncture, unable to advance the guidewire due to vasospasm, and atherosclerotic plaque, etc.);

Stage 2: Puncture and sheath placement is successful, but the catheter or guidewire cannot be advanced (arterial tortuosity, vasospasm, and loop, etc.);

Stage 3: The guidewire and catheter has reached the aortic arch, but the primary branch of the aortic arch cannot be selected;

Stage 4: The guidewire and catheter has reached the primary branch of the aortic arch, but the procedure still cannot be completed successfully;

- **Secondary Effectiveness Endpoints:**
  - Success rate of accurate diagnosis: successful accurate diagnosis is defined as the responsible vessel for the disease is successfully superselected without changing the puncture site, with angiography results meeting the diagnostic requirements.
  - Duration of angiography: Time from puncture to the end of the angiography.
  - Duration of fluoroscopy: Data from the machine will be used.
  - Bedridden time: Time (hours) from the end of the procedure to ambulation.
  - Visual Analogue Scale (VAS) score: The VAS will be completed within 24 hours after the end of the angiography. The VAS is provided in Appendix 3.

(2) Safety Endpoints:

- Incidences of angiographic complications during and within 24 hours after the procedure
- Incidences of major angiographic complications during and within 24 hours after the procedure
  - ☐ Access route associated complications (catheter kink or fracture, artery dissection, artery perforation, artery occlusion, compartment syndrome, arteriovenous fistula, retroperitoneal hematoma, hemorrhage, severe limb ischemia, embolism in any new territory, pseudoaneurysm, subcutaneous hematoma, and arterial spasm, etc.). Any complication which results in permanent sequelae, requires hospitalization, prolongation of existing hospitalization, any surgery or other medical intervention, or leads to death will be considered as a **major** complication, while all other complications will be considered as **minor** complications.
  - ☐ Neurological complications (cerebral infarction, intracranial hemorrhage, cortical blindness, nerve injury, nervous system infection, contrast encephalopathy, and vasovagal reactions including decreased

blood pressure, decreased heart rate, cold sweat, pale, clammy limbs, etc.). Any complication which results in permanent sequelae, requires hospitalization, prolongation of existing hospitalization, any surgery or other medical intervention, or leads to death will be considered as a **major** complication, while all other complications will be considered as **minor** complications.

3. Study Design

3.1 Overall Study Design:

This is a prospective, multicenter, randomized, controlled, non-inferiority study. 858 subjects are planned to be enrolled in this study, and the ratio between the TRA group and the TFA group is expected to be 1:1.

3.2 Study Contents:

1) To evaluate the safety and effectiveness of TRA for cerebral angiography

To evaluate of the safety and effectiveness of cerebral angiography via TRA approach, patients who are scheduled to undergo diagnostic cerebral angiography will be considered as subjects in this study. Patients will be continuously screened based on the inclusion and exclusion criteria, and eligible subjects will be randomly assigned to the TRA group and the TFA group at the ratio of 1:1. To analyze the safety and effectiveness of cerebral angiography via TRA, and compare the complication rate of TRA and TFA cerebral angiography, rate of successful diagnostic cerebral angiography, rate of successful accurate diagnosis, duration of angiography, duration of fluoroscopy, flat time and VAS score, so as to provide high-quality evidence-based medical data for the use of TRA cerebral angiography.

2) To analyze factors affecting cerebral angiography through TRA approach.

However, due to anatomical differences, vessel superselection in angiography and interventions through the TRA approach are more difficult to perform compared with TFA. Previous studies have shown that the difficulty of selective intubation increases gradually from right to left, with the right subclavian artery having the highest success rate and the left vertebral artery having the lowest success rate. However, it

is also found that in clinical practice, for some patients with abnormal aortic arch, vascular tortuosity or sclerosis, it is often difficult to complete routine cerebrovascular angiography diagnosis via TFA approach, while TRA can significantly improve the success rate of angiography diagnosis. Therefore, in this study, clinical and imaging data (age, diabetes, hypertension, hyperlipidemia, smoking history, Type III aortic arch configuration, looping technique, diameter and development status of the radial artery, development status of the aortic arch and the vessels above the arch and degree of tortuosity, previous operation history especially of the radial artery) of all patients undergoing TRA cerebral angiography will be investigated, to analyze the factors affecting TRA cerebral angiography.

3.3 Study Flow Diagram:

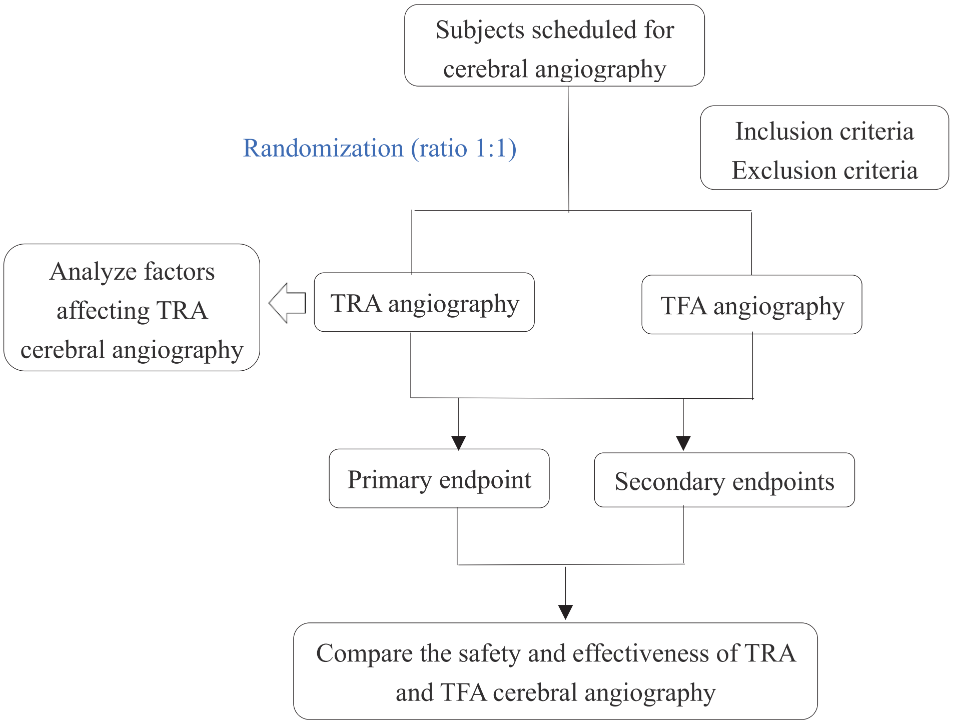

4. Study Population:

4.1 Inclusion Criteria (only patients who meet all of the following criteria can be considered to enroll into this study)

1) Age between 18 and 80 years old;
2) Patients who are scheduled to receive diagnostic cerebral angiography;

3) Patients who are suitable for cerebral angiography through transradial arterial access and transfemoral arterial access as indicated by objective evidence, with radial artery diameter ≥2mm based on ultrasonography;
4) Modified Rankin Scale (mRS) score ≤2
5) Patients who have provided written informed consent form.

Notes: If a patient is scheduled to undergo diagnostic angiography but further interventional or surgical treatment is necessary, treatment should be scheduled 24 hours after the diagnostic angiography, and this is not considered a protocol deviation.

4.2 Exclusion Criteria (Patients who meet any of the following criteria are not allowed to participate in this study)

1) Severe stenosis of radial artery, brachial artery, subclavian artery, brachiocephalic artery, iliac artery or common femoral artery as found in imaging examination, or any serious vascular disease (such as aortic aneurysm, etc.) along the path of the angiographic catheter, which may hinder the passage of a guidewire;
2) Arteriovenous fistula for hemodialysis present in the right upper limb;
3) Any planned interventional surgery within 24 hours of the first study angiography;
4) Require emergency cerebral angiography;
5) Patients who will have their procedure under general anesthesia;
6) Any contraindication to cerebral angiography, such as allergy or intolerance to the contrast media, uncorrected severe coagulation disorders, arterial dissection in the target vessel, puncture site infection, and renal insufficiency (Creatinine [Gr] > 3 times of upper limit of normal [ULN]), etc.;
7) Women who are pregnant or plan to become pregnant within 1 year;
8) Patients who are participating in any other clinical trial;
9) Any other condition which is considered by the investigator as unsuitable for participating in this study.

4.3 Criteria and Procedures for Subject Withdrawal

- Withdrawal required by the investigator:
  1) Subjects experiencing any adverse event (AE) or serious adverse event (SAE) who are required to withdraw from this study by the investigator.
  2) Subjects experiencing any other complication/clinical symptom or

special physiological change during the study, which makes the subject unsuitable to continue the study;

3) Poor compliance;

4) Any other condition that, in the opinion of the investigator, are not suitable to continue the study.

- Subjects voluntarily withdrew from the study

1) Subjects unwilling or unable to continue to participate in this clinical trial, and expressing of the willingness to withdraw from the trial for any reason;

2) Subjects who no longer accept to be followed up or lost to follow up although he/she does not explicitly expression of withdrawal from the study.

5. Study Interventions

5.1 Standard Operating Procedures for TRA and TRF Cerebral Angiography

Transfemoral cerebral angiography:

Aortic arch angiography: With the progress of angiography technique and the need of higher safety, it is currently believed that aortic arch angiography can help to preliminarily evaluate the overall intracranial and external vascular conditions, so as to find vascular orifices above the arch and select appropriate catheters, facilitating the operating for digital subtraction angiography (DSA). Aortic arch angiography is usually performed using a hydrophilic guidewire with a diameter of 0.035 inch (1 inch = 2.54 cm) and a pigtail catheter with side holes. The end of the catheter will be directly connected to the pressure extension tube of the DSA high pressure syringe by automatic injection.

Selective angiography: Standard cerebrovascular angiography is a quadruple angiography including bilateral internal carotid/common carotid artery + bilateral vertebral artery angiography. Sometimes, a bilateral external carotid artery angiography is also needed to confirm compensation of external cranial artery or to exclude dural arteriovenous fistula. However, in order to reduce the risk of plaque detachment caused by guidewire contact with the arterial plaque, in most cases, quadruple angiography (i.e. bilateral common carotid artery + bilateral subclavian artery angiography) is considered

to be sufficient to clearly observe the intracranial and extracranial vessels. Quadruple angiography is usually completed by using a hydrophilic guidewire of 0.035 inch and a single curved angiographic catheter (such as a Vertebral catheter). Manipulation steps: (1) Connection: Connect the end of the single curve catheter in conjunction with a guidewire to a Y shaped valve, and then connect it to pressure drip and high-pressure syringe via a three-way pipe to drain the air in the pipeline. (2) Catheterization: Advance the catheter to the ascending aorta through the aortic arch under the guidance of an angiographic guidewire, and withdraw the guidewire. Slowly withdraw the catheter while rotating at the same time until the tip of the catheter is cataputed into the orifice of the primary vessel of the arch. At this time, advance the guidewire to ensure the supporting force of the guidewire is sufficient to support the catheter and keep the tip of the guidewire within the safe area. Fix the guidewire and advance the catheter slowly along the guidewire. During carotid angiography, the catheter tip should be placed at 2 to 3 cm below the carotid bifurcation. During subclavian artery and vertebral artery angiography, the catheter tip should be placed in the subclavian artery at 1 to 2 cm from the orifice of the vertebral artery. (3) Angiography. Angiography with the catheter tip located in the primary branch of the aortic arch is usually called selective angiography. Angiography with the catheter tip located in any secondary or even tertiary branch, such as the internal carotid and vertebral arteries, is called super-selective angiography. The diameters of these branches are small, so it is recommended to accurately deliver the guidewire into the target blood vessel under the guidance of the road map of selective angiography, and then keep the angiography catheter coaxial with the target blood vessel and advance it to a proper position suitable for angiography. Precautions during the procedure: (1) Before super-selective angiography, the diameter and tortuosity of the target vessel should be carefully evaluated, and make judgement by considering the necessity of super-selective angiography comprehensively. Super-selective angiography should be performed with caution if there is plaque or stenosis in the vessel orifice. (2) The target vessels of super-selective angiography are more likely to be injured, and the guidewire should be pushed gently with appropriate rotation, to avoid vascular dissection. (3) If there is severe stenosis or aneurysm in the target

vessel and the effects of imaging from several projection locations is unsatisfactory, 3D imaging can be tried to obtain a more comprehensive image.

Angiography in complex vessels: Tortuous artery is commonly seen in cerebral angiography, which increases the difficulty of manipulation. Selective angiography can be performed when: (1) The tortuosity of iliac artery or abdominal aorta seriously affects catheter manipulation. A long vascular sheath can be used to straighten tortuous vessels to help manipulation. (2) The orifice of the target blood vessel is distorted and steeply angled, making it difficult for the guidewire to enter. The angle of the guidewire tip can be increased by reforming the guidewire. (3) If the distal end of the target vessel is tortuous, in which the guidewire can pass but the catheter is difficult to be advanced, please try to deliver the guidewire to a relatively safe area at the distal end of the vessel, for example, to the external carotid artery or axillary artery, and slightly rotate the catheter while advancing it, and ask the patient to turn the head to the opposite side to reduce tension. (4) For bovine arch, if the catheter can be placed in the brachiocephalic trunk, but the guidewire is difficult to advance in the left common carotid artery, ask the patient to turn the head to the right or cough gently while advancing the guidewire. (5) For Type II aortic arch, if the catheter is difficult to be placed in the brachiocephalic trunk and cannot provide sufficient support for guidewire delivery, A Hunterhead catheter with a larger curved tip can be considered. (6) For Type III aortic arch or Type II aortic arch combined with bovine arch, selective angiography may be considered by using a Simmons complex curve catheter and reform the catheter for the iliac artery, left subclavian artery, or aortic valve. To avoid knots, do not over-rotate the catheter. (7) If the vessel is too tortuous, please avoid repeatedly attempting with one method; If the guidewire or the catheter still cannot be delivered to the target place after changing method and interventional materials, please stop the procedure in time to avoid the risks of increasing complications.

TRA cerebral angiography:

Catheter shaping: Catheter shaping is a process of changing the direction of the catheter tip through the aortic arch and the arch vessels using a complex cephalic angiographic catheter, to enable the catheter enter the arch artery in a retrograde

manner. It is a fundamental skill for TRA cerebral angiography. Considering the experience in Type III aortic arch via TFA and the opinion of popular surgeons, Simmons angiography catheter is quite suitable for TRA angiography. There are various techniques to shape the Simmons catheter, including shaping in the descending aorta, in the ascending aorta, in the aortic valve, and in the common carotid arteries. Each type of shaping has its own advantages and disadvantages, with application values for different vessel conditions. Simmons angiography catheters can be divided into four types based on the shape and length of the tip. Among them, Type I to III is more commonly used for TRA angiography, and each of them has its own advantages in clinical practice based on different cephalic tip lengths. In general, the shorter the tip is, the easier to form, but more difficult to enter the vessels of the aortic arch. The longer the tip is, the more difficult to form in the arch, but easier to superselect the artery. Therefore, appropriate type should be selected based on the vascular conditions of the individuals. Simmons Type II catheter is suitable for catheter shaping and superselection in most vascular conditions, and shaping in the descending aorta is the most commonly used procedure. If the tip of the Simmons Type II catheter cannot enter the descending aorta, please try to place the guidewire into the descending aorta using a pigtail catheter, and then change to Simmons Type II catheter to complete catheter shaping. Some radiologists tried to use other types of peripheral catheters in clinical practice to overcome the operating difficulties caused by vascular anatomy. Specific catheters designed to fit the characteristics of TRA neuro-intervention has also been gradually used in clinical practice, but no relevant report is available, and its advantages still need to be evaluated.

Aortic arch angiography: the steps are the same as those for TFA arteriography.

Selective angiography: After completing catheter shaping, the tip of the catheter can be advanced into the great vessels of the arch (bilateral common carotid artery, and bilateral subclavian artery) by rotation, and complete the angiography. However, superselective angiography should be performed if there is intracranial lesion or if the cervical vessel (internal carotid artery, external carotid artery, and vertebral artery) needs to be evaluated separately. Usually, the guidewire is superselected to the base of

the skull to provide sufficient support in superselecting the catheter into the target vessel, under the common carotid artery or subclavian artery road map. Changing to a stiff guidewire or a softer catheter can make the procedure easier.

The investigator's qualifications and procedures for angiography will be detailed in an SOP, which is developed separately.

5.2 Restrictions

This study does not impose any restrictions on prior treatments or concomitant medications. Perioperative medications and medications related adverse events will be recorded in the electronic case report form.

6. Study Procedures

6.1 Study Procedures

Subjects will receive relevant clinical examinations (such as blood routine test, coagulation function test, kidney function test, vascular ultrasound or computerized tomographic angiography [CTA] of limbs, and urine pregnancy test for women of childbearing potential) and mRS score assessment after signing the informed consent form. Eligible subjects who meet the eligibility criteria will be randomly assigned to one of the two groups and receive cerebral angiography via different access approaches based on randomization results, with intraoperative information recorded. Effectiveness and safety endpoints will be evaluated within 24 hours after the procedure. Subjects will be followed up by telephone at 30 days ±7 days after the procedure to see if there is any complication. Adverse events and concomitant medications/treatments during the study period will be recorded. The study flow chart is shown in Appendix 1. The laboratory test and preoperative and postoperative ultrasonic examinations in the screening period of this study are parts of the routine diagnosis and treatment in clinical practice, which will not cause additional burden to the patients.

6.2 Informed Consent and Subject Enrollment

After receiving information about the study, subjects will be asked to provide written informed consent (IC) for participating in this study. The investigator will explain appropriately based on the request of the subjects. A subject can only be screened for eligibility after both he/she (or his/her legal representative) and the investigator have signed and dated the ICF approved by the Institutional Review Board/Independent Ethics Committee (IRB/IEC), and a copy of the signed ICF will be provided to the

subject. Subjects who have provided written informed consent will be screened for evaluation of eligibility. While patients who do not provide written informed consent will receive further treatment based on the physician's recommendation and/or patient's willingness.

After obtaining consent of the subjects, the investigator will screen the subject by checking the inclusion and exclusion criteria, and confirm that a subject does not meet any item of the exclusion criteria by evaluating all the results at screening. Subjects will only be assigned to a group based on randomization if they meet all of the inclusion criteria and do not meet any of the exclusion criteria.

6.3 Randomization

After being screened and eligibility confirmed, subjects will be randomized to the test group (cerebral angiography via TRA approach) and the control group (cerebral angiography via TFA approach) at the ratio of 1:1 through a computerized central randomization system.

6.4 Clinical Investigations and Assessments

(1) Investigations before the procedure generally include:

Blood routine test (red blood cells, white blood cells, platelet count, and hemoglobin);

Renal function test (Creatinine [Cr]);

Coagulation function test (activated partial thromboplastin time [APTT] and international normalized ratio [INR]);

Ultrasound or other objective examinations of blood vessels in the extremities (e.g. CTA, etc.);

Urine human chorionic gonadotropin [HCG] (only for women of child-bearing potential).

(2) From the start of the procedure to 24 hours after the procedure:

Effectiveness endpoints: Success rate of diagnostic angiography, success rate of accurate diagnosis, duration of angiography, duration of fluoroscopy, bedridden time and VAS score (within 24 hours after the procedure);

Safety endpoints;

Vascular ultrasound examination of the puncture site will be completed within 24 hours after the end of the angiography.

(3) From discharge to 30 (±7) days after the procedure (telephone follow-up):
Adverse events, concomitant medications/treatments;

7. Statistical Analysis

7.1 General Principles

Descriptive statistics will be provided for subject distribution, demographics and baseline characteristics, major protocol deviations, disease history, and concomitant treatments, etc; Statistics including mean ± standard deviation, number of cases, median, minimum, and maximum will be presented for continuous variables, while frequency and percentage will be provided for categorical variables. All statistical analyses will be performed using SAS® 9.4 or above. For further details on the statistical analysis, please refer to the Statistical Analysis Plan.

7.2 Hypothesis Testing

Null hypothesis (H0): The success rate of cerebral angiography via TRA approach is inferior to that via TFA approach (i.e. the difference in success rates ≤-5%);

Alternative hypothesis (H1): The success rate of cerebral angiography via TRA is not inferior to that via TFA (i.e. the difference in success rates >-5%).

Chi-square test will be used for comparison between groups at one-sided significance level of 0.025. If $p<0.025$, the null hypothesis H0 is rejected, supporting the conclusion that the success rate of cerebral angiography via TRA approach is not inferior to that via TFA approach.

7.3 Sample Size Estimation

This study will test the hypothesis that the success rate of diagnostic cerebral angiography by TRA approach is not inferior to that by TFA approach.

We assume the success rates of diagnostic cerebral angiography by TRA and TFA are 97% and 98% respectively by considering the data in the references [11-13], with a margin for non-inferiority of -5%, a power of 90% at one-side significance level of 0.025, and a drop-out rate of 10%, 429 cases are needed for each group according to PASS 15 software, and thus a total of 858 subjects need to be enrolled in this study.

7.4 Datasets for Analysis

The following datasets will be established for analysis in this study:

Full Analysis Set (FAS): The FAS will include subjects randomized to receive TRA or TFA cerebral angiography, with recorded evaluation results of the diagnosis. The effectiveness will be analyzed mainly based on FAS by group of randomization.

Safety Set (SS): The SS will include all subjects randomized to receive cerebral angiography via TRA approach or TFA approach. This analysis will be performed by group of subjects based on their actually received diagnostic procedures.

Per Protocol Set (PPS): PPS is a subset of the FAS, including subjects from the FAS with no major protocol deviations. The criteria for major protocol deviations should be finalized before the lock of the database, and the lists of subjects to be included and excluded from the PPS should be reviewed and discussed by the principal investigator, the statistician, and the sponsor at the data review meeting before the lock of the database.

7.5 Handling of Missing Data

Missing data will not be imputed in this study.

7.6 Safety Analysis

Safety will be analyzed based on the SS.

7.6.1 Adverse Events (AEs)

For adverse events (AE), only treatment emergent adverse events (TEAE) will be summarized in this study, which is defined as: any adverse event that occurs during or within 30 days after the angiography. AEs will be coded using MedDRA 24.1 or higher version.

Frequency tables will be used to summarize TEAEs based on incidences by system organ class (SOC) and preferred term (PT) for each group in descending order, unless otherwise specified. If a subject experienced more than one adverse events in the same SOC or PT, only the one with the highest severity will be counted.

TEAEs of the following categories will be summarized:

- All TEAEs, and all angiography related TEAEs
- Moderate and severe TEAEs, and angiography related moderate and severe TEAEs
- Treatment emergent serious adverse events (SAE), and angiography related SAEs

Angiography related TEAEs including those with a definite, possible, indeterminate, or missing correlation to the angiography.

The incidences of angiographic complications during and within 24 hours after the procedure will be separately summarized by type (pathway-associated complications/neurological complications) and by severity (severe/mild).

7.6.2 Death

The rate of death within 30 days after the procedure and rate of angiography related death within 30 days after the procedure will be summarized, and a list of deaths of the subjects will be provided.

7.7 Effectiveness Analysis

The effectiveness will be analyzed based on FAS and PPS, and FAS will be used as the main dataset for effectiveness analysis.

7.7.1 Primary Effectiveness Analysis

The primary effectiveness endpoint of this study is the success rate of diagnostic cerebral angiography, which is defined as the aortic arch vessel is successfully selected without changing the puncture site, with angiography results meeting the diagnostic requirements. Any change of puncture site before or during the procedure after a subject has been randomized would be treated as a diagnosis failure.

The success rate and failure rate of diagnosis will be described, and the causes of failure will be summarized (see Section 2.2 Study Endpoints for details). The Clopper-Pearson method will be used to calculate the 95% confidence intervals (CI) for rate of successful diagnosis in both groups, as well as the difference in rates between the two groups and its 95% CI (Miettinen-Nurminen method). Chi-square tests will be used for non-inferiority tests for rates of successful diagnosis in both groups, and Logistic regression will be used to calculate odds ratio (OR) between the two groups and its 95% CI.

7.7.2 Analysis on Secondary Effectiveness Endpoints

A statistical description of rate of successful accurate diagnosis, duration of angiography, duration of fluoroscopy, flat time and VAS score will be provided, and the linear mixed model will be used to calculate the differences between groups and their 95% CIs using group as covariate and study center as random effect.

7.7.3 Exploratory Analysis

If data permit, Logistic regression will be used to conduct an exploratory analysis of factors affecting cerebral angiography through TRA approach. Influencing factors may include age, diabetes, hypertension, hyperlipidemia, smoking history, Type III aortic arch configuration, looping technique, diameter and development status of the radial artery, development status of the aortic arch and arch vessels and degree of tortuosity, previous history of surgery on the radial artery.

8. Data Management

All subject information, medical records, and laboratory data must be kept confidential.

The information and data will be discussed, analyzed and reported only for the purpose of this clinical study. Subject number will be used in the electronic case report form and any other report to identify the subject, and subject's identity will be kept confidential.

8.1 Completion of Case Report Form

The Case report Form (CRF) will ensure that all data required in the protocol and meeting the requirements for statistical analysis will be collected. The Electronic Case Report Form (eCRF) is an electronic form of the CRF, to record the study relevant data of each subject. The electronic case report form is required to be completed for all enrolled subjects.

Electronic Data Capture System (EDC) will be used for data acquisition and management in this study. The investigator or Clinical Research Coordinator (CRC) should fill in the eCRF accurately, timely, completely as required according to the CRF filling guidelines.

8.2 Data Verification and Query

Both automatic edit check and manual edit check will be conducted to ensure the integrity, consistency and accuracy of the study data. The EDC system will run real-time automatic logical verification for all filled data, for example, verification on the range of data values, and logical relations, etc. The data manager will send manual queries in the EDC.

8.3 Lock of the Database

The data manager will lock the database after all data entered, all queries resolved, and the principal investigator, statistical analysis personnel, clinical data monitoring representatives and the data management person have jointly completed the final definition and judgment of the analysis population and given written permission on lock of database.

Generally, the database, after being locked, cannot be unlocked again. If unlock of the database is needed, the conditions and process for unlocking must comply with the corresponding SOP, and the unlocking process must be carefully controlled and recorded.

9. Ethical Protection and Informed Consent

9.1 Ethical Considerations, Whether Involving Human Genetic Resources and Use of Highly Pathogenic Microorganisms

This is a non-inferiority study to evaluate the safety and effectiveness of TRA and TFA angiography. This study involves no blood or tissue sample collection, and no additional follow-up costs will be incurred by the patients. This study will be conducted in compliance with *Measures for the Ethical Review of Biomedical Research Involving Humans* of China and international ethical guidelines such as the *Declaration of Helsinki*. This study does not involve the use of human genetic resources or highly pathogenic microorganisms.

9.2 Study Protocol Approval

Before the clinical trial, the investigator should submit the study protocol, ICF and other relevant documents to the medical ethics committee of the hospital of the corresponding clinical trial institution. This study can only be initiated after being approved by the Ethics Committee. Any amendment to the protocol must be approved by the Ethics Committee before implementation.

9.3 Informed Consent Process and Informed Consent Form

The investigator must explain the details of the clinical study to the subject before the enrollment of that subject:

The subjects' participation of this study should be voluntary and they have the right to withdraw at any stage of the clinical study;

Personal data of the subjects will be kept confidential. Representatives from the ethics committee, and drug and device administration authorities as well as the study team members may have access to the data of the subjects, but shall not make any of the data public;

During the clinical study, the clinical study institution is obligated to provide the subjects with the information related to the clinical study;

Subjects should be aware of the contents, purpose, possible adverse events and other relevant information of the study, and can only be enrolled after confirming that they fully understand the study and signed the informed consent form.

The informed consent form shall be signed by the investigator and the subject in duplicates, with one copy to be kept by each of them.

10. Stipulation on Adverse Events

10.1 Adverse Events

10.1.1 Definition of Adverse Events

An AE is defined as any untoward medical occurrence during the course of the clinical trial. Only TEAEs will be summarized in this study, which is defined as any adverse event that occurs during or within 30 days after the angiography. AEs will be coded using MedDRA 24.1 or higher version.

TEAEs of the following categories will be summarized:

- All TEAEs, and all angiography related TEAEs
- Moderate or severe TEAEs, and angiography related moderate or severe TEAEs
- Treatment emergent serious adverse events (SAE), and angiography related SAEs

Angiography related TEAEs including all TEAEs with a definite, possible, indeterminate, or missing causal relationship to the angiography.

10.1.2 Severity of Adverse Events

Mild: A mild AE is defined as any AE that can be tolerated by the subject, which does not affect the study treatment, requires no special treatment, and has no impact on the health of the subject.

Moderate: A moderate AE is defined as any AE that cannot be tolerated by the subject, and the device needs to be withdrawn or special treatment is needed, which has a direct impact on the health of the subject.

Severe: A severe AE is defined as any AE that endangers the life of the subject, results in death or disability, and requires immediate withdrawal of the device or emergency treatment.

10.2 Serious Adverse Events

A serious adverse event (SAE) is any untoward medical occurrence in the clinical trial which:

1) Result in death;
2) Results in life-threatening disease or injury;
3) Results in persistent disability/incapacity;
4) Requires inpatient hospitalization or prolongation of hospital stay;
5) Requires medical or surgical intervention to prevent persistent disability/incapacity;
6) Is a fetal distress, fetal death, or congenital abnormality or birth defect, etc.

10.3 Possible Adverse Events

AEs that may be related with angiographic procedure include: catheter kink or fracture, artery dissection, artery perforation, artery occlusion, compartment syndrome, arteriovenous fistula, retroperitoneal hematoma, hemorrhage, severe limb ischemia, embolism in any new territory, pseudoaneurysm, subcutaneous hematoma, arterial spasm, brain infarction, intracranial hemorrhage, cortical blindness, nerve injury, nervous system infection, contrast encephalopathy, and vasovagal reactions etc.

According to *Chinese Expert Consensus on Transradial Access for Nerve Interventional Treatment*, compared with TFA, TRA is more complicated in techniques which requires higher technical skills, and has a steeper learning curve. Therefore, it is required that all investigators participating in this trial should have practical experiences on angiography via TRA and TFA approach with at least 50 angiograms performed, and a detailed SOP should be developed for the study operation, and training should be planned for the operators before the initiation of this study.

For complications at the puncture site: In the process of compression, observe regularly whether the dressing is dry, whether there is bleeding or swelling in the wound, as well as the pulsation of the dorsal foot artery, so as to detect bleeding and other complications as early as possible and manage in time. Subjects who are to receive TFA should be in supine position and immobilized on the puncture side for 24 hours. For subjects who are to receive TFA, hemostasis can be done by hand pressure or compression of the puncture site after the sheath is removed.

Radial artery spasm is a common cause of TRA failure and the conversion to other approaches. In most cases, intrathecal administration of antispasmodics can help to relieve radial artery spasm and to complete the procedure. A regional nerve block or general anesthesia may be needed for radial artery spasm that does not resolve after conventional medical intervention, sedation and analgesia, and forearm warming. Prophylactic administration of spasmolytics may reduce the risk of radial artery spasm. However, attention should be paid to the risk of hypotension caused by spasmolytics when applied in neuro-interventional procedures, especially in general anesthesia, during which blood pressure monitoring should be strengthened.

Transient ischemic attack and brain infarction: Events such as intraoperative vascular wall plaque detachment, catheter thrombosis, and gas embolism can cause ischemic stroke. Prevention methods include: Give systemic heparinization to the subjects after

successful puncture to prevent thrombosis in the catheter wall. Superselective angiography should be performed in the order of the aortic arch, the great vessels of the arch and their branches, and do not allow the catheter or guidewire to exceed the vascular wall plaque, so as to prevent plaque rupture or mural thrombosis detachment. Carefully check and drain the catheter to ensure there is no air in it, so as prevent the occurrence of air embolism; Thrombolysis or mechanical embolectomy should be done based on the condition when a distal vessel embolism is confirmed. Hyperbaric oxygen therapy can be given when air embolism occurs.

Cortical blindness: Cortical blindness is characterized by loss of binocular vision, but with normal pupil reflex to light. Patients may be other symptoms such as amnesia, hemiplegia, and headache, etc., which is most commonly seen after vertebral arteriography, and other cerebrovascular or coronary arteriography. The pathogenesis is associated with cerebral vasospasm and destruction of blood-brain barrier, which may be a similar type of disease with reversible posterior leukoencephalopathy syndrome. There is no treatment with special effect for cortical blindness after cerebrovascular angiography, so it is necessary to improve the head imaging examination to exclude the posterior circulating cerebral embolism. Appropriate fluid replenishing can be used to promote the excretion of contrast agent, and antispasmolytic drugs can be given at the same time. Cortical blindness usually has a good prognosis, with complete recovery within several hours or days.

Artery dissection: Dissection occurring in the femoral artery or iliac artery is usually not detected in time as the puncture needles, catheter and guidewire enters under the intima. The dissection is retrograde dissection which is not easy to continue to expand as the intima rupture is located in the distal segment of the vascular dissection while the vascular dissection is located in the proximal segment. Generally, it can heal by itself after a few hours or days. Blood supply to the contralateral great vessels may be affected if the vascular dissection extends too deeply, for which local angiography should be performed in time, and assistance from the surgery department should be requested if necessary. Arterial dissections occurring in the superior arch vessel are anterograde dissections, for which the interventional procedure should be suspended

immediately and angiography should be performed a few minutes later. No special treatment is needed if there is no significant lumen stenosis or significant contrast agent retention in the vessel wall. If the luminal flow is significantly affected, stent placement can be considered.

Vasovagal reflex: When the peripheral blood vessels are stimulated during the removal of the vascular sheath, hand pression, or pression by using bandage, patients may experience vagus reflex, which is mainly manifested as decreased blood pressure and heart rate. Vagus reflex symptoms such as cold sweat, pallor, and clammy limbs may also occur. For the elderly or cardiac insufficiency patients, vagal reflex can be life-threatening. Methods for treatment include relief of vascular irritation, intravenous injection of atropine, supplement of blood volume appropriately, and use of vasoactive drugs such as dopamine to increase blood pressure when necessary.

Hematoma formation: Inguinal hematoma is the most common complication of the puncture site. Causes include abnormal coagulation function or use of any anticoagulant drug; repeated puncture of the femoral artery during the procedure, or bilateral puncture of the femoral artery with its branches involved at the same time; inappropriate method or insufficient time of compression hemostasis at the puncture site of the femoral artery after the procedure, and symptoms that increasing abdominal pressure, such as severe cough, and constipation; and early weight-bearing activities of the lower limb of the puncture side. Prevention methods include: Confirm that the patient has no coagulation dysfunction before the procedure, and control the amount of heparin appropriately based on the time of the procedure; Minimize the number of femoral artery punctures; ensure the site of postoperative pressing is appropriate, and the time of pressing should be no less than 15 minutes; Ask the patient to avoid severe cough and keep flat in bed for at least 24 hours. Minor bleeding can be managed by mechanical compression. Most cases of hematomas are self-limiting and can be absorbed by the patients.

Pseudoaneurysm: After femoral artery puncture, blood can enter the perivascular tissue through the damaged wall and form a space, resulting in pseudoaneurysm. Systole causes arterial blood flowing through the neck to the lumen while diastole causes blood returning to the artery. Causes of pseudoaneurysm include: too many puncture times;

low puncture site and thin femoral artery results in relatively large puncture wound; more perivascular soft tissue, which makes compression hemostasis difficult; Large size of arterial sheath. Most pseudoaneurysms can be locally wrapped with pressure on the neck of the pseudoaneurysms under ultrasonic localization, and the cavity can be closed 3-5 days thereafter. For pseudoaneurysms for which compression occlusion is difficult, thrombin can be injected under ultrasound. In rare cases, the pseudoaneurysm can be occluded with drug coated stents, or surgically removed or repaired.

The incidence of neurological complications caused by cerebral angiography is low, and there is currently no direct evidence on the risk of neurological complications after TRA angiography. Multiple studies have shown that no serious neurological complications occur in the TRA or TFA group.

10.4 Determination of Relationship Between Adverse Events and the Angiography

Relationship with the angiography (1) There is a reasonable temporal relationship between the two; (2) The event is a known risk of angiography, or can be explained by the mechanism of angiography; (3) The event is relieved or disappeared after stopping the angiography; (4) The event reappears after reuse of angiography; (5) The event could not be explained by other contributing factors. Any event that meets all of the five items above will be considered as "definitely related" to the angiography; while any event meeting two of the above items will be considered as "possibly related" to the angiography.

Unrelated to the angiography: (1) There is no reasonable temporal relationship between the two; (2) This type of the adverse event is not possible to be caused by the angiography; (3) The adverse event can be explained by the concomitant use of any device/drug, subject's disease progression, or other treatment effects. Any event meeting three of the above items will be considered as "definitely unrelated" to the angiography; while any event meeting one of the above items will be considered as "unlikely related" to the angiography.

10.5 Reporting and Treatment of SAEs

Any AE or SAE observed in this clinical trial should be clearly documented in the Adverse Events section of the case report form. **For individual adverse event:** Any adverse event resulting in death should be reported within 7 days; Any adverse event that results in or may results in serious injury or death shall be reported through the

China Medical Device Adverse Event Monitoring System within 20 days; For any medical device adverse event that occurs in a group of subjects: The event should be reported to the drug regulatory administration and health administration of the province, autonomous region or municipality directly under the Central Government within 12 hours by telephone or fax, and it may be reported to the authority of the higher level if necessary. At the same time, the basic information of the adverse event shall be reported through the National Medical Device Adverse Event Monitoring System. Each incident should also be reported within 24 hours on a case-by-case basis.

11. Administration Considerations

11.1 Confidentiality of the Subjects

All subject information, medical records, and laboratory data must be kept confidential. The information and data will be discussed, analyzed and reported only for the purpose of this clinical study. Subject numbers will be used in the electronic case report form and any other report to identify the subjects, and subject's identity will be kept confidential.

11.2 Study Monitoring

This study is an investigator-initiated research project that allows appointed monitors to visit the study site regularly to assess the quality of the data and the integrity of the study. The monitor will review the study records at the site and verify them directly with the source documents, discuss the implementation of the study with the investigator, and validate the conduct of the study. In addition, this study will be evaluated by auditors appointed by Huashan Hospital as well as inspectors from the regulatory authority, who must be given access to the eCRFs, source documents and other study related materials. Audit reports will be kept confidential.

The investigator or designee of the investigator must be present during the monitoring visit, to review the data, answer any queries, and allow direct access to subject records (such as medical records, office files, hospital files, and other study-related files) for source data verification. The electronic case report form must be completed before each visit so that it can be checked for accuracy and completeness.

The work of each site will be periodically reviewed by quality control personnel, with quality control report prepared and any issue found being followed up until it is solved. The monitors shall cooperate during the quality control of the clinical study site.

11.3 Case Report Form and Study Records

All records should be kept confidential. Subject's personal information, including name, shall not be disclosed at any time. The subject's clinical/study data shall not be disclosed to any party other than the Sponsor or its designee and the relevant regulatory authorities. In all cases, care must be taken to maintain subject's confidentiality. On electronic case report forms and other study-related documents, subject identification will be represented by subject numbers.

The site study staff will collect study data electronically. Data will initially be collected on source files and then transcribed into electronic case report forms. The electronic case report form will not be used as the primary collection medium for any data. Data in the electronic case report form is from the source documents and must be consistent with the source documents. Any deviation in the electronic case report form from the source document must be justified and recorded. All necessary information must be recorded in the blank space on the electronic case report form. If any data is unavailable or any column requiring fulfillment is not applicable, the corresponding column must be filled as not available or not applicable instead of being left blank.

A completed electronic case report form must be prepared for each subject enrolled in this study, and each and every page of the form must be reviewed and approved by the principal investigator. The principal investigator should electronically sign the electronic data collection page to confirm that he/she has reviewed the recorded data. The principal investigator may delegate the review and validation work to a qualified physician, who will be appointed as a sub-investigator. The investigator must keep copies of the electronic case report form, including records of changes and corrections.

11.4 Financial Disclosure

The financial affairs of the study must be documented in the agreement between the sponsor and the investigator.

11.5 Data and Safety Monitoring Board

An independent data and safety monitoring board (DSMB) will be established to regularly review and analyze all safety data. The efficacy data generated from the study will be evaluated by the DSMB for potential risks and benefits. The DSMB will consist of at least 2 independent medical professionals who are not responsible for the recruitment of subjects for this study and one finance biostatistician whose identity will not be disclosed. DSMB will review the serious adverse events continuously. The DSMB will review data such as frequency and severity of all adverse events and

demographics. Specific meetings may be held during the study for any safety concerns. The DSMB will give recommendations on whether the study should be continued, adjusted, or terminated based on the results of the data review if any study termination criteria is met. Other responsibilities and details of the DSMB will be included in the DSMB Constitution.

11.6 Clinical Event Committee

An independent clinical event committee (CEC) will be established to independently and impartially evaluate the clinical safety events. The CEC will be composed of at least three independent physicians, including a CEC Chairman. Details of the responsibilities of the members of the CEC and of clinical events to be reviewed by the CEC will be included in the CEC Constitution.

11.7 Independent Imaging Assessment Committee

An independent imaging assessment committee will be established to conduct independent imaging assessment, and the primary efficacy endpoint (rate of successful angiographic diagnosis) and the secondary efficacy endpoint (rate of successful accurate diagnosis) will be independently assessed by a third party.

12. Confidentiality Principles

The investigator must keep the study results and study materials such as the study protocol confidential, and shall not disclose the information unless authorized by the sponsor in writing. Any reference of the above must be authorized in writing by the sponsor in advance.

The data of the subjects participating in the study shall be kept confidential, but the Ethics Committee, drug regulatory authority, health and family planning commission and the sponsor may check the data of subjects participating in the study in accordance with the prescribed procedures when necessary.

13. Agreement on Publication of Study Results

The final clinical study report must be approved by the sponsor and the investigator.

The study results can be published as scientific literature or submitted to the authorities. The investigator shall not use it for any other purpose without written permission of the sponsor.

Prior to the publication or presentation of the study results, the sponsor should be asked to review the manuscript and give comments within 30 days, to confirm that the

confidential information has not been disclosed and to add relevant information. In accordance with the generally accepted principles of research collaboration, the investigator should discuss the manuscript with the sponsor's relevant personnel and reach an agreement prior to publication.

14. References

[1] M. Zhou, H. Wang, X. Zeng, P. Yin, J. Zhu, W. Chen, X. Li, L. Wang, L. Wang, Y. Liu, J. Liu, M. Zhang, J. Qi, S. Yu, A. Afshin, E. Gakidou, S. Glenn, V.S. Krish, M.K. Miller-Petrie, W.C. Mountjoy-Venning, E.C. Mullany, S.B. Redford, H. Liu, M. Naghavi, S.I. Hay, L. Wang, C.J.L. Murray, X. Liang, Mortality, morbidity, and risk factors in China and its provinces, 1930-2017: a systematic analysis for the Global Burden of Disease Study 2017, Lancet, 394 (2019) 1145-1158.
[2] L. Campeau, Percutaneous radial artery approach for coronary angiography, Cathet Cardiovasc Diagn, 16 (1989) 3-7.
[3] S.S. Jolly, S. Yusuf, J. Cairns, K. Niemela, D. Xavier, P. Widimsky, A. Budaj, M. Niemela, V. Valentin, B.S. Lewis, A. Avezum, P.G. Steg, S.V. Rao, P. Gao, R. Afzal, C.D. Joyner, S. Chrolavicius, S.R. Mehta, R.t. group, Radial versus femoral access for coronary angiography and intervention in patients with acute coronary syndromes (RIVAL): a randomised, parallel group, multicentre trial, Lancet, 377 (2011) 1409-1420.
[4] Z. Wang, J. Xia, W. Wang, G. Xu, J. Gu, Y. Wang, T. Li, Transradial versus transfemoral approach for cerebral angiography: A prospective comparison, J Interv Med, 2 (2019) 31-34.
[5] J.G. Stone, B.M. Zussman, D.A. Tonetti, M. Brown, S.M. Desai, B.A. Gross, A. Jadhav, T.G. Jovin, B. Jankowitz, Transradial versus transfemoral approaches for diagnostic cerebral angiography: a prospective, single-center, non-inferiority comparative effectiveness study, J Neurointerv Surg, 12 (2020) 993-998.
[6] 郑. 陈星宇, 吉训明, 经桡动脉穿刺全脑血管造影的可行性和安全性, 中华老年心脑血管病杂志, 16: (2014) 54-58.
[7] A.E. Kenawy, W. Tekle, A.E. Hassan, Improved Fluoroscopy and Time Efficiency with Radial Access for Diagnostic Cerebral Angiography, J Neuroimaging, 31 (2021) 67-70.
[8] D.A. Wilkinson, N. Majmundar, J.S. Catapano, V.L. Fredrickson, D.D. Cavalcanti, J.F. Baranoski, C. Rutledge, A.F. Ducruet, F.C. Albuquerque, Transradial cerebral angiography becomes more efficient than transfemoral angiography: lessons from 500 consecutive angiograms, J Neurointerv Surg, 14 (2022) 397-402.
[9] B.M. Snelling, S. Sur, S.S. Shah, S. Chen, S.A. Menaker, D.J. McCarthy, D.R. Yavagal, E.C. Peterson, R.M. Starke, Unfavorable Vascular Anatomy Is Associated with Increased Revascularization Time and Worse Outcome in Anterior Circulation Thrombectomy, World Neurosurg, 120 (2018) e976-e983.
[10] A. Maud, R. Khatri, M.R.A. Chaudhry, A. Vellipuram, S. Cruz-Flores, G.J. Rodriguez, Transradial Access Results in Faster Skin Puncture to Reperfusion Time than Transfemoral Access in Posterior Circulation Mechanical Thrombectomy, J Vasc Interv Neurol, 10 (2019) 53-57.
[11] Wang, Z., Xia, J., Wang, W., Xu, G., Gu, J., Wang, Y., & Li, T. (2019). Transradial versus transfemoral approach for cerebral angiography: A prospective comparison. Journal of interventional medicine, 2(1), 31–34.
[12] Stone, J. G., Zussman, B. M., Tonetti, D. A., Brown, M., Desai, S. M., Gross, B. A., Jadhav, A., Jovin, T. G., & Jankowitz, B. (2020). Transradial versus transfemoral approaches for diagnostic cerebral angiography: a prospective, single-center, non-inferiority comparative effectiveness study. Journal of neurointerventional surgery, 12(10), 993–998.
[13] Bhatia, K., Guest, W., Lee, H., Klostranec, J., Kortman, H., Orru, E., Qureshi, A., Kostynskyy, A., Agid, R., Farb, R., Radovanovic, I., Nicholson, P., Krings, T., & Pereira, V. M. (2021). Radial

vs. Femoral Artery Access for Procedural Success in Diagnostic Cerebral Angiography : A Randomized Clinical Trial. Clinical neuroradiology, 31(4), 1083–1091.

Appendix 1 Study Flow Chart

Study activities	Screening period [a]	Baseline	Follow-up period	
Visit time [b]	V0	V1	V2	V3
	Day -14 to Day 0	Day 0	24 hours after the procedure	30 days ±7 days after the procedure
Informed consent	X			
Demographics	X			
Inclusion/exclusion criteria	X			
Medical history, etc.	X			
Vital signs [c]	X			
mRS score	X			
Laboratory test [d]	X			
Vascular ultrasound or CTA [e]	X		X	
Randomization and study intervention		X		
Effectiveness endpoints [f]		X		
VAS score			X	
Angiographic complications (Angiography associated TEAEs)		X		
Other TEAEs		X		
Concomitant medications/treatments		X		

[a] The screening visit and baseline visit may be on the same day;

[b] The time window for V3 is ±7 days, for which telephone follow-up is acceptable.

[c] Measurements for vital signs include blood pressure and heart rate;

[d] Blood routine test (red blood cells, white blood cells, platelet count, and hemoglobin), renal function test (Cr), coagulation function test (APTT and INR), and urine HCG test (only for women of child bearing potential);

[e] Examination in the screening period will be vascular ultrasound or CTA of the four extremities, to evaluate the vascular conditions of the subjects, for which results within 90 days prior to informed consent are acceptable; while the corresponding examination within 24 hours after the procedure will be Vascular ultrasound of the puncture site, to evaluate the conditions of arterial occlusion.

[f] Effectiveness endpoints include rate of successful diagnostic angiography, rate of successful accurate diagnosis, duration of angiography, duration of fluoroscopy, flat time and VAS score. VAS score will be completed within 24 hours after the procedure, and other items should be done as soon as possible after the procedure.

[g] The laboratory test and preoperative and postoperative ultrasonography during the screening period of this study are parts of the routine diagnosis and treatment in clinical practice, which will not cause additional burden to the patients.

Appendix 2 Modified Rankin Scale (mRS)

Symptoms of the patients	Score
No symptoms at all	0
No significant disability despite symptoms; able to carry out all usual duties and activities	1
Slight disability: unable to carry out all previous activities, but able to look after own affairs without assistance	2
Moderate disability; requiring some help, but able to walk without assistance	3
Moderately severe disability: unable to walk without assistance, and unable to attend to own bodily needs without assistance	4
Severe disability; bedridden, incontinent and requiring constant nursing care and attention	5

Appendix 3 Visual Analogue Scale (VAS)

0: None Agonizing: 10

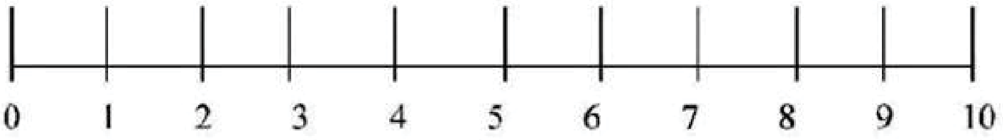

Huashan Hospital, Fudan University

STATISTICAL ANALYSIS PLAN

**Protocol Title**	:	TransRadial versus transfemoral Arterial access for CErebral angiography (TRACE)<br>(A prospective, multicenter, randomized, controlled clinical study)
**Protocol Number**	:	TRACE
**Version**	:	1.0
**Effective Date**	:	2025-01-24

Description:

- The purpose of this Statistical Analysis Plan (SAP) is to describe the planned efficacy and safety analyses and output to be included in the Clinical Study Report (CSR) for Protocol TRACE.
- Any post-hoc, or unplanned analyses performed to provide results for inclusion in the CSR but not specified in this SAP will be clearly specified in the CSR.

Author's Name, Institution, and Job Title:

Author: Tang, Hongyu
dMed Biopharmaceutical Co., Ltd. Biotatistician

Approval and Signature Page

Approved by:

Name, Institution, and Job Title	Signature	Date
Gu, Yuxiang<br>Huashan Hospital, Fudan University<br>Professor		

Reviewers

The following reviews of the SAP were conducted:

Name and Title	Role	Version Last Reviewed	Company/ Organization
Zhou, Liang, Senior Biostatistician	Review Biostatistician	1.0	Caidya
Chen, Dan, Biostatistician	Support Biostatistician	1.0	Caidya
Gu, Yuxiang, Professor	Clinical Scientist	1.0	Huashan Hospital, Fudan University
Ni, Wei, Professor	Clinical Scientist	1.0	Huashan Hospital, Fudan University
Yang, Heng, Professor	Clinical Scientist	1.0	Huashan Hospital, Fudan University

Version History

This Statistical Analysis Plan (SAP) for study TRACE is based on the protocol 1.0 dated 27Feb2023

SAP Version	Effective Date	Author	Summary of Changes
1.0	2025-01-24	Tang, Hongyu	Original version

Table of Contents

1. Summary of Key Protocol Information 7
1.1. Changes to the Protocol Defined Statistical Analysis Plan 7
1.2. Study Objectives and Endpoints 8
1.3. Study Design 9
2. Statistical Hypotheses 10
3. Sample Size Considerations 10
4. Planned Analyses 11
4.1. Interim Analyses 11
4.2. Final Analyses 11
5. Analysis Sets 11
5.1. Protocol Deviations 13
6. Considerations for Data Analyses and Data Handling Conventions 13
7. Study Population Analyses 14
7.1. Overview of Planned Study Population Analyses 14
7.2. Specifications of Study Population Analyses 17
7.2.1. Subject Disposition 17
7.2.2. Demographic and Baseline Characteristics 17
7.2.3. Prior Medical History 17
7.2.4. Current Admission Diagnosis 17
7.2.5. Pre-Opreation Imaging Examination 18
7.2.6. Concomitant Medications 18
8. Efficacy Analyses 18
8.1. Primary Efficacy Endpoint(s) Analyses 18
8.1.1. Overview of Planned Primary Efficacy Endpoint(s) Analyses 18
8.1.2. Specifications of Primary Efficacy Endpoint(s) Analyses 19
8.2. Secondary Efficacy Endpoints Analyses 22
8.2.1. Overview of Planned Secondary Efficacy Endpoints Analyses 22
8.2.2. Specifications of Secondary Efficacy Endpoints Analyses 24
8.3. Subgroup Analysis 26
9. Safety Analyses 26
9.1. Overview of Planned Safety Endpoints Analyses 26
9.2. Specifications of Safety Endpoints Analyses 27
9.2.1. Adverse Events 27
9.2.2. Death 28
10. Exploratory Analyses 28
10.1. Overview of Planned Exploratory Endpoints Analyses 28
10.2. Specifications of Exploratory Endpoints Analyses 29
10.2.1. Definitions and Derivations 29

10.2.2. Analysis Methods....30
10.2.3. Model Specifications 30

11. Multiplicity 31

12. References....32

13. Appendices....33

13.1. Appendix1: Visit and Analysis Windows....33
13.2. Appendix2: Data Display Standards & Handling Conventions....33
13.3. Appendix3: Premature Withdrawals & Handling of Missing Data....36
13.4. Appendix4: Listing of Abbreviations 37

1. Summary of Key Protocol Information

1.1. Changes to the Protocol Defined Statistical Analysis Plan

Any changes from the originally planned statistical analysis specified in the protocol 1.0 dated 27 Feb 2023 are outlined in Table 1.

Table 1 Changes to Protocol Defined Analysis Plan

Protocol	Statistical Analysis Plan	
**Statistical Analysis Plan**	**Statistical Analysis Plan**	**Rationale for Changes**
• The success rate and failure rate of diagnosis will be described, and the causes of failure will be summarized (see Section 2.2 Study Endpoints for details). The Clopper-Pearson method will be used to calculate the 95% confidence intervals (CI) for rate of successful diagnosis in both groups, as well as the difference in rates between the two groups and its 95% CI (Miettinen-Nurminen method). Chi-square tests will be used for non-inferiority tests for rates of successful diagnosis in both groups, and Logistic regression will be used to calculate odds ratio (OR) between the two groups and its 95% CI.	• The Wald method will be used to calculate the 95% confidence intervals (CI) for rate of successful diagnosis in TRA amd TFA groups<br>• The difference in rates between TRA amd TFA groups will be presented with its 95% CI according to Wald method.<br>• A Wald Z test will be used for the non-inferiority hypothesis of the primary efficacy endpoint with a non-inferiority margin of -5%.<br>• A generalized linear regression with log link function will also be used to calculate risk ratio (RR) between the two groups and its 95% CI.	• To keep the consistency between 95% confidence interval and non-inferiority test, the method for calculating confidence intervals for success rates and rate differences was changed to Wald method. And the statistical test was updated from Chi-square test to Wald Z test<br>• Since this is a prospective randomized controlled study, we used risk ratio(RR) instead of odds ratio(OR) for between-group comparison

1.2. Study Objectives and Endpoints

Objectives	Endpoints
**Primary Objectives**	**Primary Endpoints**
To demonstrate that the success rate of diagnostic cerebral angiography via TRA (Transradial Access) is not inferior to that via TFA (Transfemoral Access).	The rate of successful diagnostic cerebral angiography.<br>Successful diagnostic angiography: the aortic arch vessel is successfully superselected without changing the puncture site, with angiography results meeting the diagnostic requirements.
**Secondary Objectives**	**Secondary Endpoints**
To evaluate the efficacy of cerebral angiography via TRA approach.	• Rate of successful accurate diagnosis<br>• Duration of angiography<br>• Duration of fluoroscopy<br>• Flat time<br>• Visual Analogue Scale (VAS) score (within 24h)
**Safety Objectives**	**Safety Endpoints**
To evaluate the safety of diagnostic cerebral angiography via TRA.	• Incidences of angiographic complications during and within 24 hours after the procedure<br>• Incidences of major angiographic complications during and within 24 hours after the procedure
**Exploratory Objectives**	**Exploratory Endpoints**
To analyze factors affecting cerebral angiography via TRA approach.	• Clinical and imaging data (age, diabetes, hypertension, hyperlipidemia, smoking history, Type III aortic arch configuration, looping technique, diameter and development status of the radial artery, development status of the aortic arch and the vessels above the arch and degree of tortuosity, previous operation history especially of the radial artery) of all patients undergoing TRA cerebral angiography will be investigated, to analyze the factors affecting TRA cerebral angiography.

1.3. Study Design

Overview of Study Design and Key Features

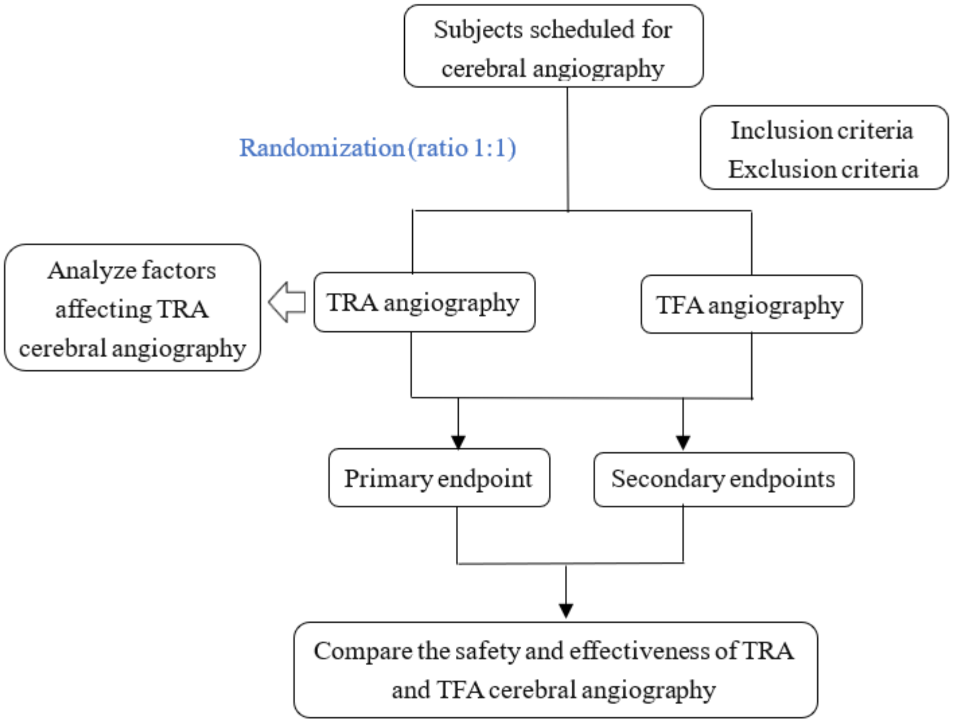

**Design Features**	• This is a prospective, multicenter, randomized, controlled, non-inferiority study. 858 subjects are planned to be enrolled in this study, and the ratio between the TRA group and the TFA group is ≈ 1:1.
**Study Interventions**	• Test group: cerebral angiography via TRA approach<br>• Control group: cerebral angiography via TFA approach
**Assignment**	• Eligible subjects will be randomized to the TRA group and the TFA group at the ratio of 1:1 through a computerized central randomization system.
**Interim Analysis**	Interim analysis will be conducted by the Data Safety Monitoring Board (DSMB) after the 30-day follow-up of 430 participants. The Haybittle-Peto method will be used to control the overall Type I error rate: the p-value boundary for the one-sided test at the interim analysis will be set at 0.001, and the p-value boundary for the one-sided test at the final analysis will be set at 0.024.

Overview of Study Design and Key Features	
**Data and Safety Monitoring Board (DSMB)**	• An independent data and safety monitoring board (DSMB) will be established to regularly review and analyze all safety data.<br>• The efficacy data generated from the study will be evaluated by the DSMB for potential risks and benefits.<br>• DSMB will review the serious adverse events continuously. The DSMB will review data such as frequency and severity of all adverse events and demographics.<br>• Specific meetings may be held during the study for any safety concerns.<br>• The DSMB will give recommendations on whether the study should be continued, adjusted, or terminated based on the results of the data review if any study termination criteria is met. Other responsibilities and details of the DSMB will be included in the DSMB Charter.

2. Statistical Hypotheses

Null hypothesis (H0): The success rate of cerebral angiography via TRA approach is inferior to that via TFA approach (i.e. the difference in success rates ≤-5%);

Alternative hypothesis (H1): The success rate of cerebral angiography via TRA is not inferior to that via TFA (i.e. the difference in success rates >-5%).

The Wald Z test will be used for comparison between groups at one-sided significance level of 0.025. If p<0.025, the null hypothesis H0 is rejected, supporting the conclusion that the success rate of cerebral angiography via TRA approach is not inferior to that via TFA approach.

3. Sample Size Considerations

This study will test the hypothesis that the success rate of diagnostic cerebral angiography by TRA approach is not inferior to that by TFA approach.

The success rates of diagnostic cerebral angiography by TRA and TFA are assumed to be 97% and 98% respectively. With a margin for non-inferiority of -5% and a drop-out rate of 10%, 429 cases are needed for each group to achieve a power of 90% at one-sided significance level of

0.025, according to PASS 15 software, and thus a total of 858 subjects need to be enrolled in this study.

4. Planned Analyses

4.1. Interim Analyses

An independent data and safety monitoring board (DSMB) will be established to regularly review and analyze all safety data and/or efficacy data for the evaluation of potential risks and benefits. Other responsibilities and details of the DSMB will be included in the DSMB Charter.

An interim analysis will be conducted by the DSMB after the 30-day follow-up of 430 participants. The Haybittle-Peto method will be used to control the overall Type I error rate: the p-value boundary for the one-sided test at the interim analysis will be set at 0.001, and the p-value boundary for the one-sided test at the final analysis will be set at 0.024.

4.2. Final Analyses

The final planned primary analyses will be performed after the completion of the following sequential steps:

1. All subjects have completed or discontinued the study as defined in the protocol.
2. All required database cleaning activities have been completed and database freeze has been declared by Data Management.
3. Final database release.
4. Randomisation codes have been distributed according to SOP.

5. Analysis Sets

Population	Definition / Criteria	Analyses Evaluated
Full Analysis Set (FAS)	• The FAS will include subjects randomized to receive TRA or TFA cerebral angiography, with recorded evaluation results of the diagnosis.	• Population analyses<br>• Efficacy

Population	Definition / Criteria	Analyses Evaluated
	• Statistical analysis will be performed according to the intervention group randomly assigned (regardless of the actual intervention received).<br>• The FAS is the main set for the efficacy analysis of this study.	
Safety Set (SS)	• The SS will include all subjects randomized and received cerebral angiography via TRA approach or TFA approach.<br>• This analysis will be performed according to the actual intervenrion received.	• Safety
Per Protocol Set (PPS)	• PPS is a subset of the FAS, including subjects from the FAS with no major protocol deviations.<br>• The criteria for major protocol deviations should be finalized before the lock of the database, and the lists of subjects to be included and excluded from the PPS should be reviewed and discussed by the principal investigator, the statistician, and the sponsor at the data review meeting before the lock of the database.<br>• PPS-based analyses will serve as supportive analyses to complement FAS-based analyses.	• Efficacy

5.1. Protocol Deviations

- Major protocol deviations will be summarised and all protocol deviations will be listed by subject.
- Major deviations which result in exclusion from the PPS will also be summarised and listed.
- Protocol deviations will be tracked by the study team throughout the conduct of the study in accordance with the Medical Monitoring Plan.
  - Data will be reviewed prior to freezing the database to ensure all major deviations and deviations which may lead to exclusion from the analysis are captured and categorized on the protocol deviations dataset.

6. Considerations for Data Analyses and Data Handling Conventions

All statistical analyses will be performed by SAS ® system 9.4 (or higher version). Tables, figures and listings will be generated as RTF files.

Study population analyses, efficacy analyses and safety analyses will be performed by intervention groups, which will be represented by ‘Transradial Access’ and ‘Transfemoral Access’.

Unless otherwise specified, categorical variables will be summarized by number (n) and percentages (%) of subjects under each category, as well as number and percentages of subjects with missing categories. Continuous variables will be summarized by number of non-missing subjects (n), arithmetic mean, standard deviation (SD), median, minimum, and maximum.

Table 2 provides an overview of appendices within the SAP for outlining general considerations for data analyses and data handling conventions.

Table 2 Overview of Appendices

**Component**
Appendix 1: Visit and Analysis Windows
Appendix 2: Data Display Standards & Handling Conventions
Appendix 3: Premature Withdrawals & Handling of Missing Data
Appendix 4: Listing of Abbreviations

7. Study Population Analyses

7.1. Overview of Planned Study Population Analyses

The study population analyses will be based on the FAS population, unless otherwise specified.

Table 3 provides an overview of the planned study population analyses.

Table 3 Overview of Planned Study Population Analyses

	**Data Displays Generated**		
	Table	Figure	Listing
**Subject Disposition**			
Number of subjects screened			
Subjects discontinued prior to randomization<br>− Screen failure and reasons<br>− Withdrawal by subject<br>− Other			
Randomized subjects			
Subjects who received cerebral angiography			
Subjects who did not receive cerebral angiography and reasons			
Subjects who completed the study			
Subjects who discontinued from the study prematurely and primary reasons			
Full Analysis Set			
Safety Set			
Per Protocol Set			
**Demographics and Baseline Characteristics**			
Sex	Y		Y

	Data Displays Generated		
	Table	Figure	Listing
Age (years)			
Race			
Baseline body weight (kg)			
Body height (cm)			
Baseline BMI (kg/m^2)			
Modified Rankin Scale (mRS) score			
Smoking history			
Alcohol history			
**Prior Medical History**			
Diabetes (Yes/ No/ Unknown)	Y		Y
Type of Diabetes (Type I/ Type II/ Other)			
Hypertension (Yes/ No/ Unknown)			
Hyperlipidemia (Yes/ No/ Unknown)			
Coronary Heart Disease (Yes/ No/ Unknown)			
Type of Coronary Heart Disease (Acute Myocardial Infarction /Old Myocardial Infarction /Unstable Angina/ Stable Angina/ Other)			
Cerebrovascular Disease (Yes/ No/ Unknown)			
Type of Cerebrovascular Disease (Ischemic/ Hemorrhagic/ Mixed/ Other)			
Puncture History (Yes/ No/ Unknown)			

	Data Displays Generated		
	Table	Figure	Listing
Puncture Site (Left Radial Artery/ Right Radial Artery/ Left Femoral Artery/ Right Femoral Artery/ Other)			
Purpose of Puncture (Cerebrovascular Intervention/ Cardiovascular Intervention/ Other)			
Aortic Surgery History (Yes/ No/ Unknown)			
Puncture sites of aortic surgery (left radial artery/ right radial artery/ left femoral artery/ right femoral artery/ other)			
Allergy History (Yes/ No/ Unknown)			
**Current Admission Diagnosis**			
Current Admission Diagnosis: SOC and PT	Y		Y
**Pre-Opreation Imaging Examination**			
Left Radial Artery Diameter (mm)	Y		Y
Right Radial Artery Diameter (mm)			
Left Femoral Artery Diameter (mm)			
Right Femoral Artery Diameter (mm)			
Left Radial Artery Growth Status (Normal/Abnormal)			
Left Radial Artery Abnormal Category			
Right Radial Artery Growth Status (Normal/Abnormal)			
Right Radial Artery Abnormal Category			
**Concomitant Medications**			

	Data Displays Generated		
	Table	Figure	Listing
Concomitant Medications by ATC[level 3] and PN	Y		Y

7.2. Specifications of Study Population Analyses

7.2.1. Subject Disposition

Based on all screened subjects, the number and percentage of randomized subjects and those who discontinued prior to randomization along with the reasons will be summarized.

Based on all enrolled subjects, the number and percentage of subjects will be summarized:

- Subjects who received cerebral angiography
- Subjects who did not receive cerebral angiography and reasons
- Subjects who completed the study
- Subjects who discontinued from the study prematurely and primary reasons
- Subjects included in each analysis set.

7.2.2. Demographic and Baseline Characteristics

Demographics and baseline characteristics will be summarized for the FAS.

- Age (years), sex, race, baseline body weight and height will be collected from the "Demography" Page of Case Report Form (CRF).
- mRS score will be collected from the "Modified Rankin Scale (mRS)" Page of CRF.
- Smoking history and alcohol history will be collected from the "Smoking and Alcohol History" Page of CRF.
- BMI (kg/m2) calculated as [weight(kg)/(height (cm)/100)^2] and rounded to keep one decimal place.

7.2.3. Prior Medical History

- Prior medical history will be collected from the "Prior Medical History" Page of CRF.
- Prior medical history will be summarized for the FAS as per **Table 3**.
- All prior medical history data will be listed by subject.

7.2.4. Current Admission Diagnosis

- Current admission diagnosis will be collected from the "Cocurrent Medical History" Page of CRF.
- Current admission diagnosis will be coded using the latest version of Medical Dictionary for Regulatory Activities (MedDRA). All diagnoses will be listed, and the number and

percentage of subjects with each diagnosis will be summarized alphabetically by system organ class (SOC) and decreasing frequency by preferred term (PT).

7.2.5. Pre-Opreation Imaging Examination

- Artery diameter measurements will be collected from the "Pre-Operation Imaging Examination" Page of CRF
- Radial artery growth status will be collected from the "Radial Artery Growth Evaluation" Page of CRF

7.2.6. Concomitant Medications

- Concomitant medications will be collected from the "Concomitant Medication" Page of CRF.
- All medications will be coded and summarized using the latest version of WHO-DD, presenting the number and percentage of subjects by the Anatomical Therapeutic Chemical (ATC) classification system and the preferred name (PN); each subject could have medications under multiple ATC Level 3 groups and/or multiple preferred drug names, but each subject will be counted only once within each ATC Level 3 group and preferred name class. In case that any drug has not been assigned a Level 3 ATC group, the group with the most specific level will be used.

8. Efficacy Analyses

8.1. Primary Efficacy Endpoint(s) Analyses

8.1.1. Overview of Planned Primary Efficacy Endpoint(s) Analyses

The primary endpoint(s) analyses will be based on the FAS population, unless otherwise specified.

Table 4 provides an overview of the planned primary endpoint analyses.

Table 4 Overview of Planned Primary Endpoint(s) Analyses

	**Data Displays Generated**		
	Table	Figure	Listing
**Rate of Successful Diagnostic Cerebral Angiography**			
Descriptive statistics:<br>Number of subjects with:<br>– Successful diagnostic cerebral angiography<br>– Failed diagnostic cerebral angiography<br>○ With crossover<br>○ Without crossover	Y		Y

	Data Displays Generated		
	Table	Figure	Listing
Number of subjects with failed diagnostic cerebral angiography per stage and causes of failure			
Success rate and failure rate of diagnosis with 95% confidence intervals (CI)			
Difference in rates of successful diagnosis between TRA and TFA groups and its 95% CI			
P value for non-inferiority test			
Risk Ratio (RR) between TRA and TFA groups and its 95% CI			

8.1.2. Specifications of Primary Efficacy Endpoint(s) Analyses

8.1.2.1. Definitions and Derivations

The primary effectiveness endpoint of this study is the rate of successful diagnostic cerebral angiography, which is defined as the aortic arch vessel is successfully superselected without changing the puncture site, with angiography results meeting the diagnostic requirements. Any change of puncture site before or during the procedure after a subject has been randomized would be treated as a diagnosis failure.

8.1.2.2. Analysis Methods

8.1.2.2.1. Overview of Analysis Methods

**Analysis**	**Analysis Set**	**Statistical Analysis Methods**	**Handling of Missing Data**
**Primary Endpoint**			
Primary analysis	FAS	Normal approximation method for calculating 95% CI of rates;<br>Normal approximation method for calculating 95% CI of difference in rates;<br>Wald Z test;<br>Generalized linear regression.	Missing primary outcome will not be included in FAS.
Supplementary analysis	PPS	Same as primary analysis.	Missing primary outcome will not be included in PPS.

8.1.2.2.2. Analysis Methods of Primary Analysis

- The success rate and failure rate of diagnosis will be described, and the causes of failure will be summarized.
- The Wald method will be used to calculate the 95% confidence intervals (CI) for rate of successful diagnosis in TRA amd TFA groups
- The difference in rates between TRA amd TFA groups will be presented with its 95% CI according to Wald method.
- A Wald Z test will be used for the non-inferiority hypothesis of the primary efficacy endpoint with a non-inferiority margin of -5%.
- A generalized linear regression with log link function will also be used to calculate risk ratio (RR) between the two groups and its 95% CI. The covariates only include study group. Association between baseline factors and the success rate of diagnostic cerebral angiography will be explored in exploratory analysis section.

8.1.2.2.3. Analysis Methods of Supplementary Analysis

The analysis will be repeated in PPS using the same methods as primary analysis.

8.1.2.3. Model Specifications

8.1.2.3.1. Model Specifications of Primary Analysis

**Statistical Analysis of Binary Variables**
**Endpoints and Parameters**
• Endpoints: success rate<br>• Parameters:<br>– Rate estimate and 95% CI<br>– Rate difference and 95% CI<br>– P value<br>– Risk Ratio
**Input Variables**

- AVAL: Response variable
- TRTPN: Planned arm
- COUNT: The number of subjects in each level each group

Sample SAS code

Rate estimate and 95% CI calculation sample code:

```
PROC FREQ DATA= adeff;
     TABLE AVAL / BINOMIAL (CL=WALD) ALPHA=0.05;
     WEIGHT COUNT/zeros;
     BY TRTPN;
     ODS OUTPUT BINOMIALCLS=BinomialCL;
RUN;
```

Rate difference and 95%CI calculation sample code:

```
PROC FREQ DATA=adeff;
     TABLE TRTPN*AVAL / RISKDIFF(CL=WALD) ALPHA=0.05;
     WEIGHT COUNT/zeros;
     ODS OUTPUT PDIFFCLS = diff;
RUN;
```

Wald test for non-inferiority sample code:

```
PROC FREQ DATA=adeff;
     TABLE TRTPN*AVAL / RISKDIFF(NOINF MARGIN=.05) ALPHA=0.025;
     WEIGHT COUNT/zeros;
     ODS OUTPUT PdiffNoninf=noninf;
RUN;
```

Generalized linear regression sample code:

```
PROC GENMOD DATA=adeff DESCENDING;
```

```
   CLASS TRTPN AVAL;
   MODEL AVAL = TRTPN / DIST=BIN LINK=LOG;
         where paramcd='DCA';
   ESTIMATE 'Risk Ratio for TRTPN' TRTPN 1 -1 / EXP;
   ODS OUTPUT Estimates=RRCL;
RUN;
```

Results Presentation

- Rate estimate and 95% CI can be retained from BinomialCL.
- Rate difference and 95% CI can be retained from diff.
- P value of non-inferiority test can be retained from noninf.
- Risk Ratio between TRA and TFA groups and its 95% CI can be retained from RRCL.

8.1.2.3.2. Model Specifications of Sensitivity Analysis

Same as primary analysis.

8.2. Secondary Efficacy Endpoints Analyses

8.2.1. Overview of Planned Secondary Efficacy Endpoints Analyses

The secondary endpoints analyses for efficacy will be based on the FAS population, unless otherwise specified.

Table 5 provides an overview of the planned secondary endpoints analyses for efficacy.

Table 5 Overview of Planned Secondary Endpoints Analyses for Efficacy

	**Data Displays Generated**		
	Table	Figure	Listing
**Rate of Successful Accurate Diagnosis**			
Descriptive statistics:	Y		Y

	Data Displays Generated		
	Table	Figure	Listing
− Number of subjects with successful accurate diagnosis<br>− Success rate of accurate diagnosis with 95% CI			
Difference in rates of successful diagnosis between TRA and TFA groups and its 95% CI			
Risk Ratio (RR) between TRA and TFA groups and its 95% CI			
**Duration of Angiography (min)**			
Descriptive statistics:<br>− Number of non-missing subjects (n)<br>− Mean (SD)<br>− Median (Q1, Q3)<br>− Min, Max	Y		Y
Difference in duration of angiography between TRA and TFA groups and its 95% CI			
**Duration of Fluoroscopy (min)**			
Descriptive statistics:<br>− Number of non-missing subjects (n)<br>− Mean (SD)<br>− Median (Q1, Q3)<br>− Min, Max	Y		Y
Difference in duration of fluoroscopy between TRA and TFA groups and its 95% CI			
**Flat Time (Hours)**			
Descriptive statistics:<br>− Number of non-missing subjects (n)<br>− Mean (SD)<br>− Median (Q1, Q3)<br>− Min, Max	Y		Y
Difference in flat time between TRA and TFA groups and its 95% CI			

<table>
<tr><th rowspan="2"></th><th colspan="3">Data Displays Generated</th></tr>
<tr><th>Table</th><th>Figure</th><th>Listing</th></tr>
<tr><td colspan="4">Visual Analogue Scale (VAS) Score (within 24h)</td></tr>
<tr><td>Descriptive statistics:
– Number of non-missing subjects (n)
– Mean (standard deviation)
– Median (Q1, Q3)
– Min, Max</td><td rowspan="2">Y</td><td></td><td rowspan="2">Y</td></tr>
<tr><td>Difference in VAS score between TRA and TFA groups and its 95% CI</td><td></td></tr>
</table>

8.2.2. Specifications of Secondary Efficacy Endpoints Analyses

8.2.2.1. Definitions and Derivations

- Success rate of accurate diagnosis: successful accurate diagnosis is defined as the aortic arch vessel is successfully superselected without changing the puncture site, with angiography results meeting the diagnostic requirements.
- Duration of angiography (min): Time from puncture to the end of the angiography.
- Duration of fluoroscopy (min): data from the machine will be used.
- Flat time: Time (hours) from the end of the procedure to ambulation. If the minute of the flat time is unknown, it will be imputed as 30 minutes. If both hour and minute are unknown, it will be treated as missing data.
- Visual Analogue Scale (VAS) score: The VAS will be completed within 24 hours after the end of the angiography.

8.2.2.2. Analysis Methods

- For rate of successful accurate diagnosis, the analysis will be similar with the primary analysis.
- For continuous secondary endpoints, the linear regression model will be used to calculate the differences between groups and their 95% CIs using study group as covariate.

8.2.2.3. Model Specifications

Statistical Analysis of Continuous Variables

**Endpoints and Parameters**
• Endpoints:<br>– Duration of angiography<br>– Duration of fluoroscopy<br>– Flat time<br>– VAS score<br>• Parameters:<br>– Mean and 95% CI<br>– Difference in means and 95% CI
**Input Variables**
• AVAL: Response variable<br>• TRTPN: Planned arm
**Sample SAS code**
**Linear Regression Model method sample code:**<br>**PROC GLM** DATA = adeff;<br>CLASS TRTPN;<br>MODEL AVAL = TRTPN /SOLUTION DDFM = KR;<br>LSMEANS TRTPN /DIFF CL ALPHA = 0.05 e;<br>ODS OUTPUT LSMEANS = lsmeans DIFFS = Diffs;<br>**RUN;**
**Results Presentation**
• Estimation of mean and 95% CI for both groups:<br>○ Point estimate: Estimate variable in lsmeans dataset

- Corresponding 95% CI: Lower and Upper variables in lsmeans dataset
- Estimate and 95% CI for difference in means between groups:
  - Point estimate: Estimate variable in Diffs dataset
  - Corresponding 95% CI: Lower and Upper Variables in Diffs Dataset

8.3. Subgroup Analysis

If applicable, primary efficacy outcome and secondary efficacy outcomes will be performed in the following subgroups:

- Gender
- Age group
- Height
- BMI
- Diabetes
- Hypertension
- Hyperlipidemia
- History of coronary heart disease
- Smoking History
- History of alcohol use
- Baseline mRS score
- History of Puncture
- Classification of Aortic Arch
- Aortic Arch VariationDegree of Tortuosity of the Aortic Arch and Arch Vessels

9. Safety Analyses

9.1. Overview of Planned Safety Endpoints Analyses

The safety endpoints analyses will be based on the SS population, unless otherwise specified.

Table 6 provides an overview of the planned safety endpoints analyses.

Table 6 Overview of Planned Safety Endpoints Analyses

<table>
<tr><th rowspan="2"></th><th colspan="3">Data Displays Generated</th></tr>
<tr><th>Table</th><th>Figure</th><th>Listing</th></tr>
<tr><td colspan="4">Adverse Events (AEs)</td></tr>
<tr><td>All Treatment Emergent Adverse Events (TEAEs)</td><td rowspan="8">Y</td><td></td><td rowspan="8">Y</td></tr>
<tr><td>Angiography Related TEAEs</td><td></td></tr>
<tr><td>Moderate and Severe TEAEs</td><td></td></tr>
<tr><td>Angiography Related Moderate and Severe TEAEs</td><td></td></tr>
<tr><td>Treatment Emergent Serious Adverse Events (TESAEs)</td><td></td></tr>
<tr><td>Angiography Related TESAEs</td><td></td></tr>
<tr><td>Angiographic Complications During and Within 24 Hours After the Procedure by Type (Access Route Associated Complications/Neurological Complications)</td><td></td></tr>
<tr><td>Angiographic Complications During and Within 24 Hours After the Procedure by Severity</td><td></td></tr>
<tr><td colspan="4">Death</td></tr>
<tr><td>All Death</td><td rowspan="2">Y</td><td></td><td rowspan="2">Y</td></tr>
<tr><td>Angiography Related Death</td><td></td></tr>
</table>

9.2. Specifications of Safety Endpoints Analyses

9.2.1. Adverse Events

- For adverse events (AE), only treatment emergent adverse events (TEAE) will be summarized in this study, which is defined as: any adverse event that occurs during or within 30 days after the angiography.
- AEs will be coded using MedDRA 24.1 or higher version.
- Frequency tables will be used to summarize TEAEs based on incidences by system organ class (SOC) and preferred term (PT) for each group in descending order in total group, unless otherwise specified. If a subject experienced more than one adverse events in the same SOC or PT, only the one with the highest severity will be counted.

- The investigator will grade the severity of the AE (mild, moderate, severe). AEs with missing severity will not be imputed.
- Angiography related TEAEs including those with a definite, possible, indeterminate, or missing correlation to the angiography.
- The incidences of angiographic complications during and within 24 hours after the procedure will be separately summarized by type (pathway-associated complications/neurological complications) and by severity (major/minor).
- Angiographic complications:
  - Access route associated complications (catheter kink or fracture, artery dissection, artery perforation, artery occlusion, compartment syndrome, arteriovenous fistula, retroperitoneal hematoma, hemorrhage, severe limb ischemia, embolism in any new territory, pseudoaneurysm, subcutaneous hematoma, and arterial spasm, etc.). Any complication which results in permanent sequelae, requires hospitalization, prolongation of existing hospitalization, any surgery or other medical intervention, or leads to death will be considered as a major complication, while all other complications will be considered as minor complications.
  - Neurological complications (cerebral infarction, intracranial hemorrhage, cortical blindness, nerve injury, nervous system infection, contrast encephalopathy, and vasovagal reactions including decreased blood pressure, decreased heart rate, cold sweat, pale, clammy limbs, etc.). Any complication which results in permanent sequelae, requires hospitalization, prolongation of existing hospitalization, any surgery or other medical intervention, or leads to death will be considered as a major complication, while all other complications will be considered as minor complications.

9.2.2. Death

The rate of death within 30 days after the procedure and rate of angiography related death within 30 days after the procedure will be summarized, and a list of deaths of the subjects will be provided.

10. Exploratory Analyses

10.1. Overview of Planned Exploratory Endpoints Analyses

The exploratory endpoints analyses will be based on the TRA group population in the FAS, unless otherwise specified.

Table 7 provides an overview of the planned exploratory endpoints analyses.

Table 7 Overview of Planned Exploratory Endpoints Analyses

	Data Displays Generated		
	Table	Figure	Listing
**Association Analysis for Factors Affecting Cerebral Angiography Through TRA approach**			
Descrptive statistics:<br>– the same as subgroup factors described in section 8.3	Y		Y
Genearlized linear regression: risk ratio (RR) between successful and failed diagnostic cerebral angiography groups using TRA approach and its 95% CI<br>– Univariable results<br>– Multivariable results with stepwise selection	Y		Y

10.2. Specifications of Exploratory Endpoints Analyses

10.2.1. Definitions and Derivations

- Age (years) will be collected from the "Demography" Page of Case Report Form (CRF).
- Smoking history will be collected from the "Smoking and Alcohol History" Page of CRF.

- History of diabetes, hypertension, hyperlipidemia, and previous history of surgery on the radial artery will be collected from the "Prior Medical History" Page of CRF.
- Type III aortic arch configuration, Development status and degree of tortuosity of the aortic arch and arch vessels will be collected from the "Details of the Angiographic Procedure" Page of CRF.
- Diameter of the radial artery will be collected from the "Details of Preoperative Imaging Examination" Page of CRF.
- Development status of the radial artery will be collected from the "Radial Artery Development Assessment" Page of CRF.

10.2.2. Analysis Methods

- If data permit, a generalized linear regression with stepwise selection of covariates will be used to conduct an exploratory analysis of factors affecting cerebral angiography through TRA approach.

10.2.3. Model Specifications

**Statistical Analysis of Exploratory Variables**
**Endpoints and Parameters**
• Endpoints: successful/ failed cerebral angiography through TRA approach<br>• Parameters:<br>– RR and 95% CI
**Input Variables**
• AVAL: Response variable<br>• COVARIATES: Influencing factors
**Sample SAS code**
**Generalized linear regression sample code:**<br>**PROC GENMOD DATA=adeff;**<br>**CLASS TRTPN;**<br>**MODEL AVAL = COVARIATES / DIST=BIN LINK=LOG SELECTION=STEPWISE;**<br>**RR COVARIATES / CL;**

```
  ODS OUTPUT ParameterEstimates=RRResults;
RUN;
```

Results Presentation
• The RR and 95% Wald CI for the ratio can be obtained from RRResults.

11. Multiplicity

Not applicable.

12. References

[1]. TransRadial versus transfemoral Arterial access for CErebral angiography (TRACE) (A prospective, multicenter, randomized, controlled clinical study) Version 1.0 dated Feb 27, 2023.
[2]. Case Report Form: TRACE_eCRF_V1.1_20240409
[3]. ICH E9: Statistical Principles for Clinical Trials

13. Appendices

13.1. Appendix1: Visit and Analysis Windows

Visit and Analysis Windows

Study Day

Day 1 is defined as the day of the angiography.

Study days after Day 1 are calculated as the number of days from the angiography date:

- Ref Date = Missing → Study Day = Missing
- Ref Date < the Angiography Date→ Study Day = Ref Date –the Angiography Date
- Ref Data ≥ the Angiography Date→ Study Day = Ref Date –the Angiography Date + 1

Time Points and Assessment Windows

Please see Appendix 1 Study Flow Chart in study protocol.

Safety Reporting Period defined as time within 30 days after the angiography.

**Parameters**	**Target Date/Time**	**Assessment Window**	
		**Start Timepoint**	**End Timepoint**
TEAE	Start Date	The angiography date	30 calendar days after the angiography
Concomitant medications/treatments	End Date	The angiography date	-

13.2. Appendix2: Data Display Standards & Handling Conventions

Data Display Standards & Handling Conventions

Precision

Data Display Standards & Handling Conventions
• Numeric data will be reported at the precision collected on the eCRF.<br>• The reported precision from non eCRF sources will follow the same principles as described below but may be adjusted to a clinically interpretable number of decimal places (dp.).<br>• Statistics on derived data will be 1 more dp than original data. For example, if the original data has 1 dp, then the derived data will have in 2 dp; mean, minimum and maximum, and SD of this derived data will have 3 dp, 2 dp, and 4 dp, respectively.<br>• Reasonable number of dps will be adjusted for proper presentation and interpretation. The maximum number of decimal places reported shall be 4 for any summary statistic, unless otherwise stated.<br>• Maximal digits should be available in ADaM datasets.

Data Display Standards & Handling Conventions

Precision

Name	Description	Decimal Place (dp)
N	Number of Subjects in the Intervention Group	Always 0 dp.
n	Number of Subjects with Non-Missing Values	Always 0 dp.
%	Percentage	1 dp. for categorical data
Mean	Arithmetical Mean	1 more dp. than original data
SD	Standard Deviation	2 more dp. than original data
Median	Median	1 more dp. than original data
Min.	Minimum	Same as original data
Max.	Maximum	Same as original data
Q1	First Quartile	1 more dp. than original data
Q3	Third Quartile	1 more dp. than original data
SE	Standard Error	1 more dp. than statistical parameter
95% CI	95% Confidence Interval	1 more dp. than statistical parameter
P value	P value	3 dp., or <0.001

Baseline Definition & Derivations

In general, the last-non-missing measurement/assessment prior to the angiography date will serve as the baseline measurement for population analyses.

Data Handling Conventions

Data Display Standards & Handling Conventions
**Planned and Actual Time**<br>• Unless otherwise specified, unscheduled visits and visits outside the protocol defined time-windows (i.e. recorded as protocol deviations) will only be included in listings.<br>**Calculation of Proportions**<br>• Percentages will not be presented for zero counts.<br>• Unless otherwise specified, the calculation of proportions will be based on the number of participants of the analysis population of interest. Therefore, counts of missing observations will be included in the denominator.<br>**Transformation of Time Period**<br>• 1 Year = 365.25 Days<br>• 1 Month = 30.4375 Days<br>• 1 Week = 7 Days

13.3. Appendix3: Premature Withdrawals & Handling of Missing Data

Premature Withdrawals & Handling of Missing Data
**Subjects Withdrawn Early**
Subjects will not be replaced regardless of withdrawal from the trial for any reason.
**Handling of Missing Data**
Missing data will not be imputed in this study.

13.4. Appendix4: Listing of Abbreviations

Abbreviation	Description
AE	Adverse Events
ATC	Anatomical Therapeutic Chemical
CI	Confidence Interval
CRF	Case Report Form
CSR	Clinical Study Report
DSMB	Data and Safety Monitoring Board
FAS	Full Analysis Set
MedDRA	Medical Dictionary for Regulatory Activities
mRS	Modified Rankin Scale
OR	Odds Ratio
PN	Preferred Name
PPS	Per Protocol Set
PT	Preferred Term
RR	Risk Ratio
SAP	Statistical Analysis Plan
SD	Standard Deviation
SOC	System Organ Class
SS	Safety Set
TEAEs	Treatment Emergent Adverse Events
TESAEs	Treatment Emergent Serious Adverse Events
TFA	Transfemoral Access

Abbreviation	Description
TRA	Transradial Access
TRACE	TransRadial versus transfemoral Arterial access for CErebral angiography
VAS	Visual Analogue Scale score
