# Supplementary material for: Transradial vs Transfemoral Access for Cerebral Angiography: A Randomized Noninferiority Clinical Trial
Source: JAMA Netw Open. 2026 Mar 19;9(3):e261929. doi: 10.1001/jamanetworkopen.2026.1929 (PMC13003373; doi:10.1001/jamanetworkopen.2026.1929)
Supplement: Supplement 2. — eMethods. Follow-up Schedule and Inclusion and Exclusion Criteria eFigure 1. Overview of the TRACE Trial eFigure 2. Number of Patients Recruited by Each Center eFigure 3. Subgroup Analysis of Primary Outcome in Per-Protocol Analysis eFigure 4. Subgroup Analysis of Secondary Outcome (Success Rate of Accurate Diagnosis) eFigure 5. Subgroup Analysis of Secondary Outcome (Duration of Angiography) eFigure 6. Subgroup Analysis of Secondary Outcome (Duration of Fluoroscopy) eFigure 7. Subgroup Analysis of Secondary Outcome (Bedridden Time) eFigure 8. Subgroup Analysis of Secondary Outcome (VAS Score) eTable 1. Additional Baseline and Procedural Characteristics eTable 2. Primary and Secondary Outcomes in Per-Protocol Analysis eTable 3. Primary and Secondary Outcomes in As-Treated Analysis eTable 4. Safety Outcomes Assessed by the CEC-ITT eTable 5. Subgroup Analysis of Primary Outcome in Intention-to-Treat Analysis eTable 6. Subgroup Analysis of Primary Outcome in Per-Protocol Analysis eReferences [file jamanetwopen-e261929-s002.pdf]

Supplementary Online Content

Ni W, Yang H, Su J, et al; TRACE Investigators. Transradial vs transfemoral access for cerebral angiography: a randomized noninferiority clinical trial. *JAMA Netw Open.* 2026;9(3):e261929.
doi:10.1001/jamanetworkopen.2026.1929

eMethods. Follow-up Schedule and Inclusion and Exclusion Criteria
eFigure 1. Overview of the TRACE Trial
eFigure 2. Number of patients Recruited by Each Center
eFigure 3. Subgroup Analysis of Primary Outcome in Per-Protocol Analysis
eFigure 4. Subgroup Analysis of Secondary Outcome (Success Rate of Accurate Diagnosis)
eFigure 5. Subgroup Analysis of Secondary Outcome (Duration of Angiography)
(Duration of Fluoroscopy)
eFigure 7. Subgroup Analysis of Secondary Outcome (Bedridden Time)
eFigure 8. Subgroup Analysis of Secondary Outcome (VAS Score)
eTable 1. Additional Baseline and Procedural Characteristics
eTable 2. Primary and Secondary Outcomes in Per-Protocol Analysis
eTable 3. Primary and Secondary Outcomes in As-Treated Analysis
eTable 4. Safety Outcomes Assessed by the CEC-ITT
eTable 5. Subgroup Analysis of Primary Outcome in Intention-to-Treat Analysis
eTable 6. Subgroup Analysis of Primary Outcome in Per-Protocol Analysis
eReferences

This supplementary material has been provided by the authors to give readers additional information about their work.

© 2026 Ni W et al. *JAMA Network Open*.

1. **eMethods.** Follow-up Schedule and Inclusion and Exclusion Criteria

1.1. Follow-up Schedule

Study activities	Screening period [*]	Baseline	Follow-up period	
Visit time [†]	V0	V1	V2	V3
	Day -14 to Day 0	Day 0	24 hours after the procedure	30 days ±7 days after the procedure
Informed consent	X			
Demographics	X			
Inclusion/exclusion criteria	X			
Medical history, etc.	X			
Vital signs [‡]	X			
mRS score	X			
Laboratory test [§]	X			
Vascular ultrasound or CTA [¶]	X		X	
Randomization and study intervention		X		
Effectiveness endpoints [‖]		X		
VAS score			X	
Angiographic complications (Angiography associated TEAEs)		X		
Other TEAEs		X		
Concomitant medications/treatments		X		

© 2026 Ni W et al. *JAMA Network Open*.

[*] The screening visit and baseline visit may be on the same day;

[†] The time window for V3 is ±7 days, for which telephone follow-up is acceptable.

[‡] Measurements for vital signs include blood pressure and heart rate;

[§] Blood routine test (red blood cells, white blood cells, platelet count, and hemoglobin), renal function test (Cr), coagulation function test (APTT and INR), and urine HCG test (only for women of child bearing potential);

[¶] Examination in the screening period will be vascular ultrasound or CTA of the four extremities, to evaluate the vascular conditions of the subjects, for which results within 90 days prior to informed consent are acceptable; while the corresponding examination within 24 hours after the procedure will be Vascular ultrasound of the puncture site, to evaluate the conditions of arterial occlusion.

[‖] Effectiveness endpoints include rate of successful diagnostic angiography, rate of successful accurate diagnosis, duration of angiography, duration of fluoroscopy, flat time and VAS score. VAS score will be completed within 24 hours after the procedure, and other items should be done as soon as possible after the procedure.

© 2026 Ni W et al. *JAMA Network Open*.

1.2. **Inclusion and Exclusion Criteria**

Inclusion criteria:

1) Age between 18 and 80 years old;
2) Patients who are scheduled to receive diagnostic cerebral angiography;
3) Patients who are suitable for cerebral angiography through transradial arterial access and transfemoral arterial access as indicated by objective evidence, with radial artery diameter ≥2mm based on ultrasonography;
4) Modified Rankin Scale (mRS) score ≤2;
5) Patients who have provided written informed consent form;

Exclusion criteria:

1) Severe stenosis of radial artery, brachial artery, subclavian artery, brachiocephalic artery, iliac artery or common femoral artery as found in imaging examination, or any serious vascular disease (such as aortic aneurysm, etc.) along the path of the angiographic catheter, which may hinder the passage of a guidewire;
2) Arteriovenous fistula for hemodialysis present in the right upper limb;
3) Any planned interventional surgery within 24 hours of the first study angiography;
4) Require emergency cerebral angiography;
5) General anesthesia is used;
6) Any contraindication to cerebral angiography, such as allergy or intolerance to the contrast media, uncorrected severe coagulation disorders, arterial dissection in the target vessel, puncture site infection, and renal insufficiency (Creatinine [Gr] > 3 times of upper limit of normal [ULN]), etc.;
7) Women who are pregnant or plan to become pregnant within 1 year;
8) Patients who are participating in any other clinical trial;
9) Any other condition which is considered by the investigator as unsuitable for participating in this study;

© 2026 Ni W et al. *JAMA Network Open*.

2. eFigures

eFigure 1. Overview of the TRACE Trial

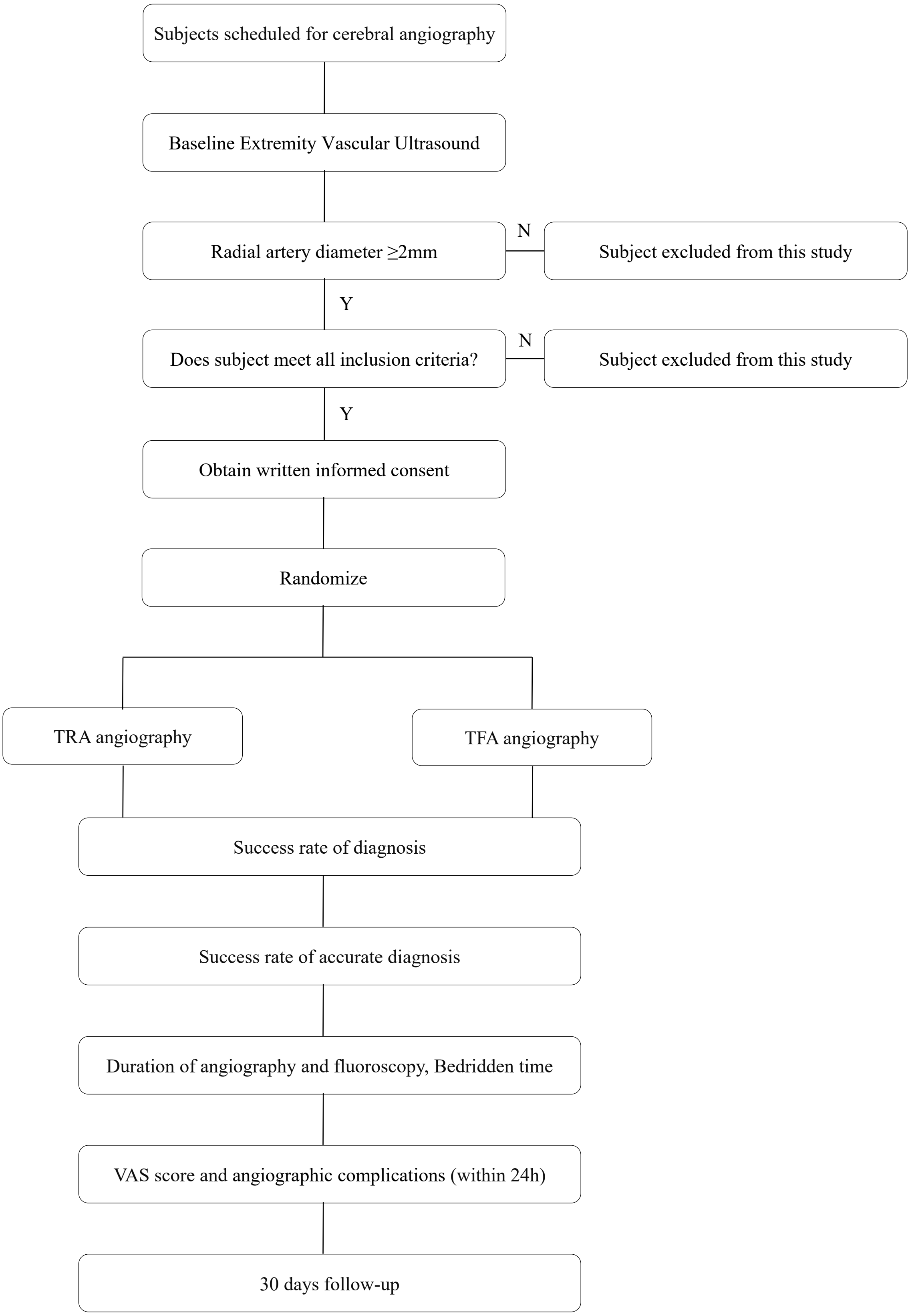

© 2026 Ni W et al. *JAMA Network Open*.

eFigure 2. Number of Patients Recruited by Each Center

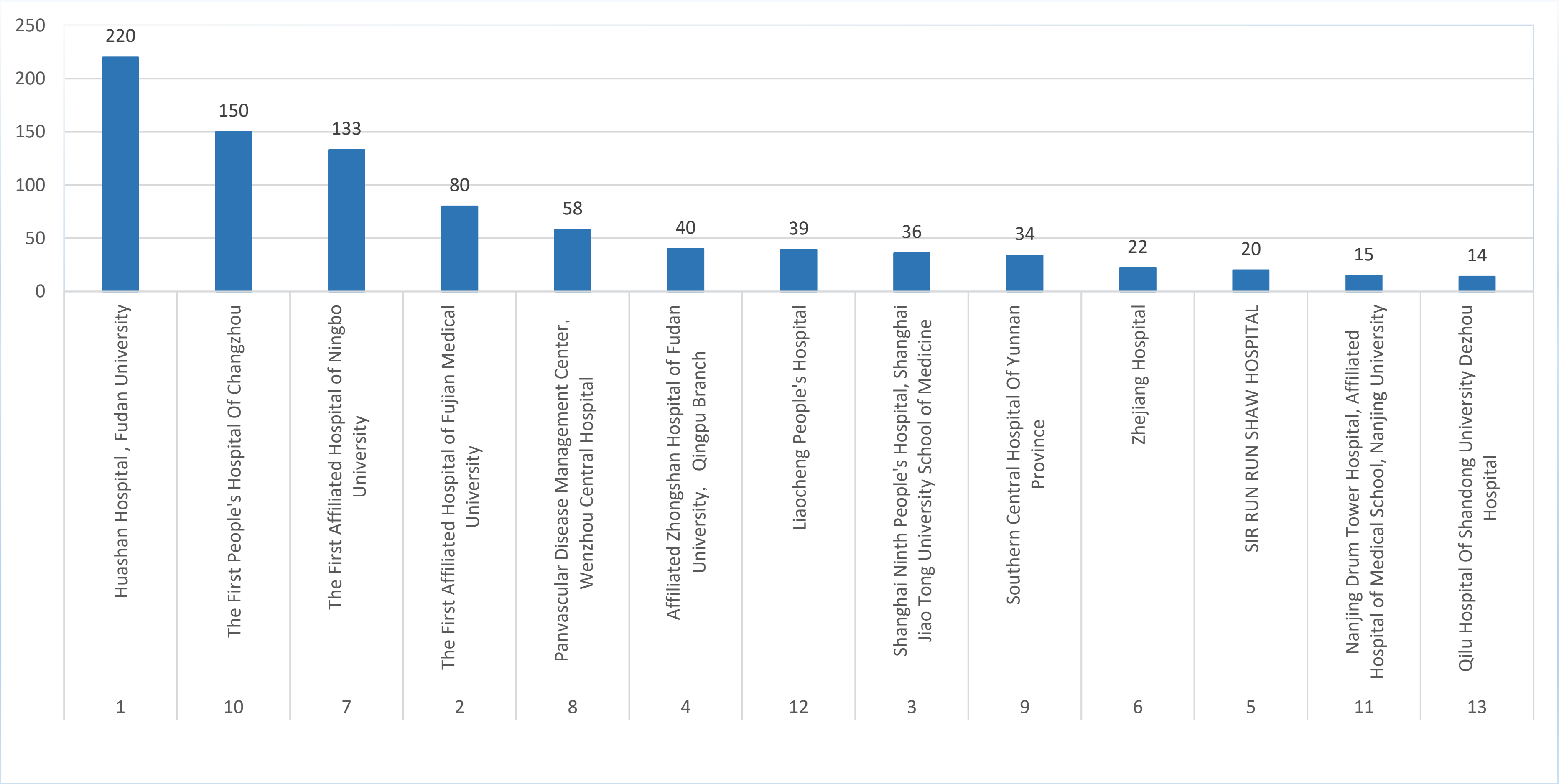

© 2026 Ni W et al. *JAMA Network Open*.

eFigure 3. Subgroup Analysis of Primary Outcome in Per-Protocol Analysis

Subgroup	TRA Successful Cases/Number of Subjects	TFA Successful Cases/Number of Subjects	Rate Difference (95% CI)
All subjects	392/431	409/427	-4.8 (-8.1, -1.5)
Age			
< 60	193/210	226/235	-4.3 (-8.7, 0.2)
>=60	199/221	183/192	-5.3 (-10.2, -0.3)
Sex			
Male	224/244	220/235	-1.8 (-6.5, 2.8)
Female	168/187	189/192	-8.6 (-13.3, -3.9)
Height			
<=175cm	363/398	371/387	-4.7 (-8.1, -1.2)
>175cm	29/33	38/40	-7.1 (-20.1, 5.9)
BMI			
<=25	241/264	229/237	-5.3 (-9.4, -1.2)
>25	151/167	180/190	-4.3 (-9.8, 1.2)
Smoking History			
Yes	48/51	47/51	2.0 (-7.8, 11.8)
No	344/380	362/376	-5.8 (-9.3, -2.2)
Alcohol Consumption History			
Yes	36/38	41/43	-0.6 (-10.1, 8.9)
No	356/393	368/384	-5.2 (-8.8, -1.7)
mRS			
Score 0	306/337	330/341	-6.0 (-9.6, -2.4)
Score 1	70/76	64/70	0.7 (-8.3, 9.6)
Score 2	15/17	14/15	-5.1 (-24.9, 14.7)
Score 3	1/1	1/1	- (-, -)
Hypertension			
Yes	227/255	226/236	-6.7 (-11.4, -2.1)
No	165/176	183/191	-2.1 (-6.6, 2.5)
Diabetes			
Yes	59/66	53/56	-5.2 (-14.7, 4.2)
No	333/365	356/371	-4.7 (-8.3, -1.2)
Hyperlipidemia			
Yes	56/63	51/54	-5.6 (-15.4, 4.3)
No	336/368	358/373	-4.7 (-8.2, -1.2)
Coronary Artery Disease			
Yes	11/15	15/16	-20.4 (-45.7, 4.9)
No	381/416	394/411	-4.3 (-7.6, -1.0)
Previous Puncture History			
Yes	128/145	134/139	-8.1 (-14.2, -2.0)
No	264/286	275/288	-3.2 (-7.1, 0.7)
Aortic Arch Classification			
Type I+II	284/306	295/305	-3.9 (-7.4, -0.4)
Type III	77/87	80/84	-6.7 (-14.8, 1.4)
Aortic Arch Variation			
Normal	325/349	339/352	-3.2 (-6.5, 0.1)
Abnormal	36/44	36/37	-15.5 (-28.0, -2.9)
Supra-aortic Artery Tortuosity			
Normal	81/83	79/80	-1.2 (-5.3, 2.9)
Tortuous	275/304	292/305	-5.3 (-9.3, -1.3)
Kinking and Looping	6/7	5/5	-14.3 (-40.2, 11.6)

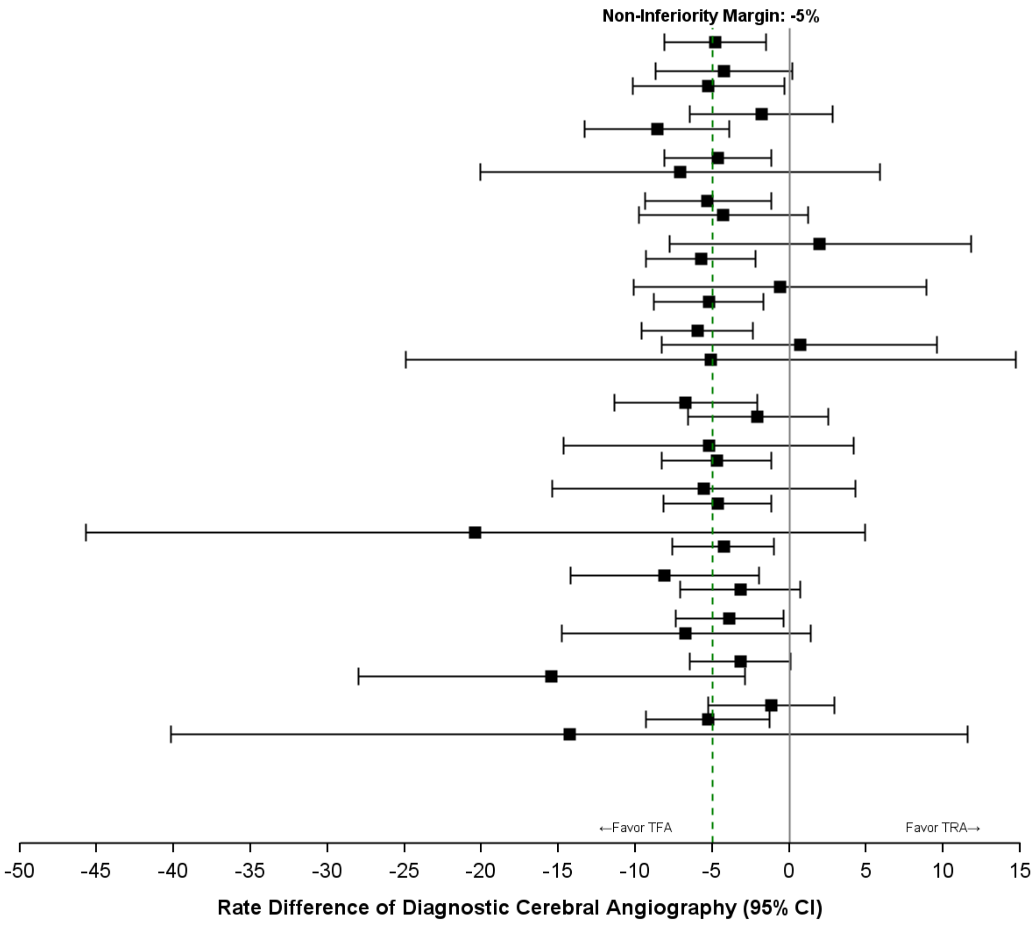

© 2026 Ni W et al. *JAMA Network Open*.

eFigure 4. Subgroup Analysis of Secondary Outcome (Success Rate of Accurate Diagnosis)

Subgroup	TRA Successful Cases/Number of Subjects	TFA Successful Cases/Number of Subjects	Rate Difference (95% CI)
All subjects	340/431	389/427	-12.2 (-16.9, -7.5)
Age			
< 60	163/210	212/235	-12.6 (-19.4, -5.8)
>=60	177/221	177/192	-12.1 (-18.6, -5.6)
Sex			
Male	194/244	209/235	-9.4 (-15.9, -3.0)
Female	146/187	180/192	-15.7 (-22.5, -8.8)
Height			
<=175cm	314/398	353/387	-12.3 (-17.2, -7.4)
>175cm	26/33	36/40	-11.2 (-28.0, 5.6)
BMI			
<=25	214/264	217/237	-10.5 (-16.4, -4.6)
>25	126/167	172/190	-15.1 (-22.8, -7.3)
Smoking History			
Yes	43/51	47/51	-7.8 (-20.3, 4.6)
No	297/380	342/376	-12.8 (-17.9, -7.7)
Alcohol Consumption History			
Yes	31/38	41/43	-13.8 (-27.6, 0.1)
No	309/393	348/384	-12.0 (-17.0, -7.0)
mRS			
Score 0	266/337	314/341	-13.2 (-18.4, -7.9)
Score 1	60/76	60/70	-6.8 (-19.1, 5.5)
Score 2	13/17	14/15	-16.9 (-40.7, 6.9)
Score 3	1/1	1/1	- (-, -)
Hypertension			
Yes	191/255	215/236	-16.2 (-22.6, -9.8)
No	149/176	174/191	-6.4 (-13.1, 0.2)
Diabetes			
Yes	52/66	49/56	-8.7 (-21.8, 4.4)
No	288/365	340/371	-12.7 (-17.8, -7.7)
Hyperlipidemia			
Yes	46/63	47/54	-14.0 (-28.2, 0.1)
No	294/368	342/373	-11.8 (-16.8, -6.8)
Coronary Artery Disease			
Yes	10/15	14/16	-20.8 (-49.7, 8.0)
No	330/416	375/411	-11.9 (-16.7, -7.2)
Previous Puncture History			
Yes	111/145	126/139	-14.1 (-22.5, -5.7)
No	229/286	263/288	-11.2 (-16.9, -5.6)
Aortic Arch Classification			
Type I+II	248/306	284/305	-12.1 (-17.3, -6.8)
Type III	68/87	75/84	-11.1 (-22.0, -0.2)
Aortic Arch Variation			
Normal	289/349	323/352	-9.0 (-13.8, -4.1)
Abnormal	27/44	36/37	-35.9 (-51.2, -20.6)
Supra-aortic Artery Tortuosity			
Normal	68/83	74/80	-10.6 (-20.7, -0.5)
Tortuous	242/304	281/305	-12.5 (-18.0, -7.1)
Kinking and Looping	6/7	5/5	-14.3 (-40.2, 11.6)

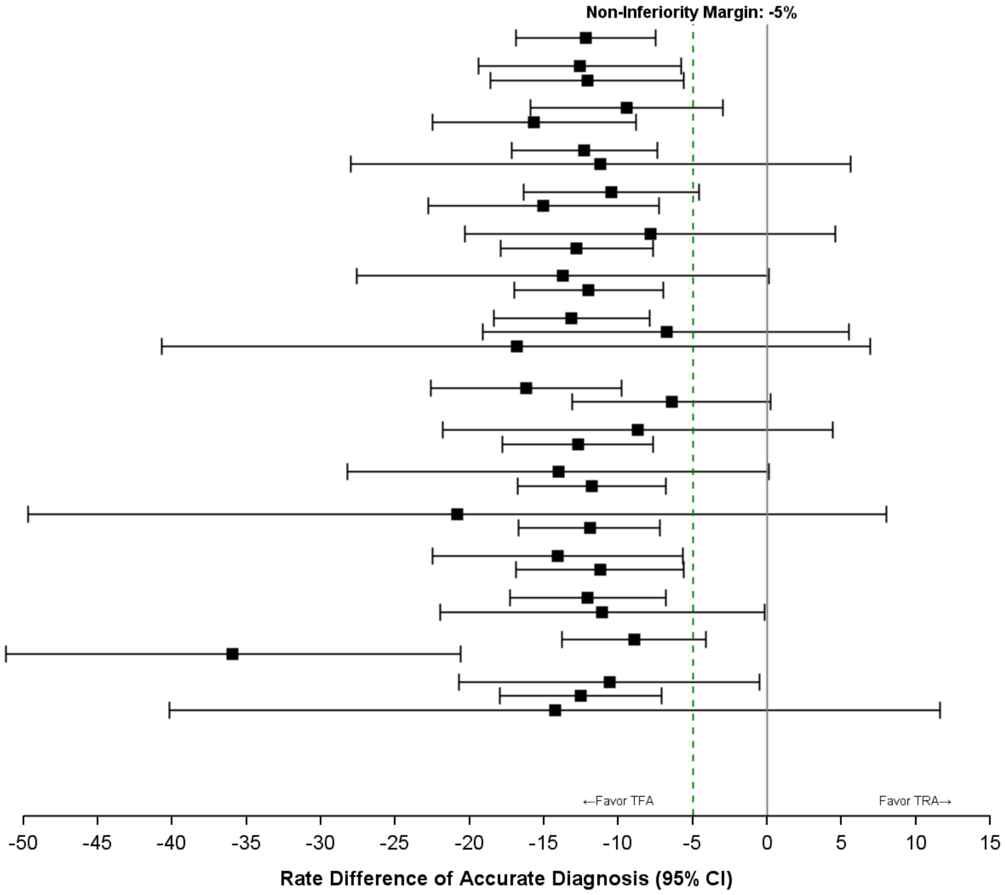

© 2026 Ni W et al. *JAMA Network Open*.

eFigure 5. Subgroup Analysis of Secondary Outcome (Duration of Angiography)

Subgroup	TRA Mean(SD)/n	TFA Mean(SD)/n	Mean Difference (95% CI)
All subjects	38.65(18.367)/431	33.73(15.139)/427	4.92 (2.65, 7.18)
Age			
< 60	38.79(17.924)/210	31.91(13.978)/235	6.88 (3.87, 9.88)
>=60	38.52(18.817)/221	35.96(16.209)/192	2.56 (-0.86, 5.98)
Sex			
Male	37.93(17.551)/244	33.37(16.326)/235	4.56 (1.51, 7.61)
Female	39.59(19.386)/187	34.18(13.573)/192	5.41 (2.02, 8.80)
Height			
<=175cm	38.82(18.225)/398	33.70(14.168)/387	5.13 (2.83, 7.43)
>175cm	36.52(20.179)/33	34.08(22.728)/40	2.44 (-7.58, 12.46)
BMI			
<=25	38.90(18.871)/264	32.68(13.542)/237	6.22 (3.29, 9.16)
>25	38.25(17.589)/167	35.05(16.866)/190	3.20 (-0.38, 6.79)
Smoking History			
Yes	38.67(14.702)/51	35.35(15.402)/51	3.31 (-2.57, 9.20)
No	38.64(18.820)/380	33.51(15.110)/376	5.13 (2.68, 7.58)
Alcohol Consumption History			
Yes	36.45(14.005)/38	35.16(15.038)/43	1.28 (-5.10, 7.67)
No	38.86(18.735)/393	33.57(15.161)/384	5.29 (2.87, 7.70)
mRS			
Score 0	38.35(18.502)/337	33.15(15.118)/341	5.20 (2.64, 7.76)
Score 1	40.22(17.931)/76	35.51(14.090)/70	4.71 (-0.59, 10.01)
Score 2	38.53(18.399)/17	39.40(19.401)/15	-0.87 (-14.13, 12.39)
Score 3	22.00(-)/1	22.00(-)/1	0.00 (-, -)
Hypertension			
Yes	40.18(18.782)/255	34.93(15.268)/236	5.25 (2.19, 8.31)
No	36.43(17.564)/176	32.25(14.885)/191	4.17 (0.84, 7.51)
Diabetes			
Yes	41.11(19.210)/66	36.80(18.411)/56	4.30 (-2.45, 11.06)
No	38.20(18.202)/365	33.27(14.555)/371	4.94 (2.54, 7.33)
Hyperlipidemia			
Yes	38.10(16.239)/63	39.94(20.475)/54	-1.85 (-8.53, 4.83)
No	38.74(18.725)/368	32.83(14.011)/373	5.91 (3.51, 8.31)
Coronary Artery Disease			
Yes	49.67(32.053)/15	36.06(16.332)/16	13.60 (-4.70, 31.90)
No	38.25(17.616)/416	33.64(15.104)/411	4.61 (2.36, 6.86)
Previous Puncture History			
Yes	42.50(19.426)/145	33.91(13.797)/139	8.59 (4.59, 12.59)
No	36.70(17.519)/286	33.65(15.768)/288	3.05 (0.32, 5.78)
Aortic Arch Classification			
Type I+II	38.09(18.796)/306	32.51(14.932)/305	5.58 (2.87, 8.29)
Type III	39.55(17.077)/87	38.12(16.007)/84	1.43 (-3.55, 6.41)
Aortic Arch Variation			
Normal	37.33(17.469)/349	33.54(15.403)/352	3.79 (1.34, 6.24)
Abnormal	47.00(23.192)/44	35.43(14.653)/37	11.57 (2.70, 20.43)
Supra-aortic Artery Tortuosity			
Normal	34.88(15.597)/83	30.08(18.137)/80	4.80 (-0.42, 10.03)
Tortuous	39.20(18.593)/304	34.30(13.930)/305	4.89 (2.27, 7.52)
Kinking and Looping	45.00(33.744)/7	55.40(25.755)/5	-10.40 (-47.30, 26.50)

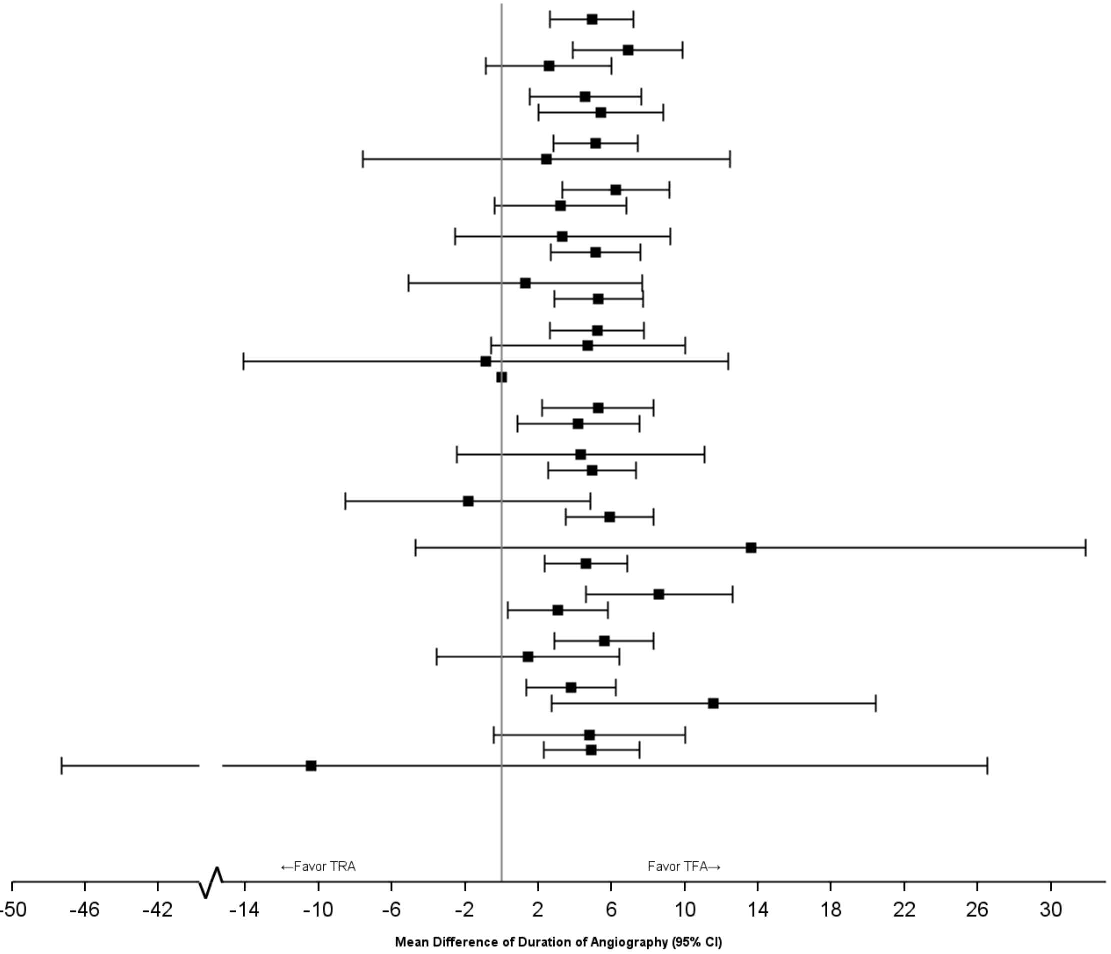

© 2026 Ni W et al. *JAMA Network Open*.

eFigure 6. Subgroup Analysis of Secondary Outcome (Duration of Fluoroscopy)

Subgroup	TRA Mean(SD)/Subjects	TFA Mean(SD)/Subjects	Mean Difference (95% CI)
All subjects	11.83(8.125)/431	10.57(7.742)/427	1.26 (0.19, 2.32)
Age			
< 60	11.35(7.184)/210	9.12(5.645)/235	2.24 (1.03, 3.44)
>=60	12.28(8.921)/221	12.35(9.426)/192	-0.07 (-1.85, 1.70)
Sex			
Male	11.80(7.620)/244	10.24(8.025)/235	1.56 (0.15, 2.96)
Female	11.87(8.762)/187	10.98(7.382)/192	0.89 (-0.74, 2.53)
Height			
<=175cm	11.86(8.194)/398	10.76(7.944)/387	1.10 (-0.03, 2.23)
>175cm	11.47(7.342)/33	8.77(5.148)/40	2.70 (-0.23, 5.62)
BMI			
<=25	11.98(8.509)/264	10.36(8.160)/237	1.62 (0.15, 3.08)
>25	11.60(7.497)/167	10.84(7.200)/190	0.76 (-0.77, 2.29)
Smoking History			
Yes	10.65(6.044)/51	9.57(6.570)/51	1.08 (-1.38, 3.54)
No	11.99(8.359)/380	10.71(7.886)/376	1.28 (0.12, 2.44)
Alcohol Consumption History			
Yes	10.43(6.378)/38	10.07(6.995)/43	0.36 (-2.58, 3.30)
No	11.96(8.269)/393	10.63(7.828)/384	1.34 (0.20, 2.47)
mRS			
Score 0	12.16(8.358)/337	10.57(7.922)/341	1.59 (0.36, 2.82)
Score 1	11.18(7.639)/76	10.70(7.454)/70	0.48 (-1.98, 2.94)
Score 2	8.30(4.004)/17	10.36(4.965)/15	-2.06 (-5.25, 1.13)
Score 3	10.00(-)/1	6.70(-)/1	3.30 (-38.63, 45.23)
Hypertension			
Yes	12.65(8.276)/255	11.96(8.702)/236	0.69 (-0.81, 2.20)
No	10.64(7.772)/176	8.86(5.951)/191	1.78 (0.36, 3.20)
Diabetes			
Yes	12.66(8.459)/66	12.44(11.191)/56	0.21 (-3.29, 3.71)
No	11.68(8.066)/365	10.29(7.056)/371	1.39 (0.29, 2.49)
Hyperlipidemia			
Yes	12.08(7.003)/63	15.10(12.355)/54	-3.03 (-6.63, 0.58)
No	11.79(8.310)/368	9.92(6.594)/373	1.87 (0.79, 2.95)
Coronary Artery Disease			
Yes	11.43(5.718)/15	12.96(7.824)/16	-1.52 (-6.45, 3.40)
No	11.84(8.203)/416	10.48(7.734)/411	1.36 (0.27, 2.45)
Previous Puncture History			
Yes	12.38(8.243)/145	9.69(7.270)/139	2.69 (0.86, 4.51)
No	11.55(8.065)/286	11.00(7.938)/288	0.55 (-0.76, 1.86)
Aortic Arch Classification			
Type I+II	11.64(7.633)/306	9.57(6.829)/305	2.06 (0.91, 3.22)
Type III	13.05(10.144)/87	14.04(9.601)/84	-0.99 (-3.96, 1.98)
Aortic Arch Variation			
Normal	11.53(8.006)/349	10.49(7.310)/352	1.03 (-0.10, 2.17)
Abnormal	15.28(9.523)/44	10.96(11.029)/37	4.32 (-0.22, 8.87)
Supra-aortic Artery Tortuosity			
Normal	10.21(5.592)/83	8.08(5.692)/80	2.13 (0.38, 3.88)
Tortuous	12.41(8.775)/304	10.91(7.374)/305	1.50 (0.21, 2.79)
Kinking and Looping	12.67(9.543)/7	27.05(23.280)/5	-14.38 (-35.01, 6.26)

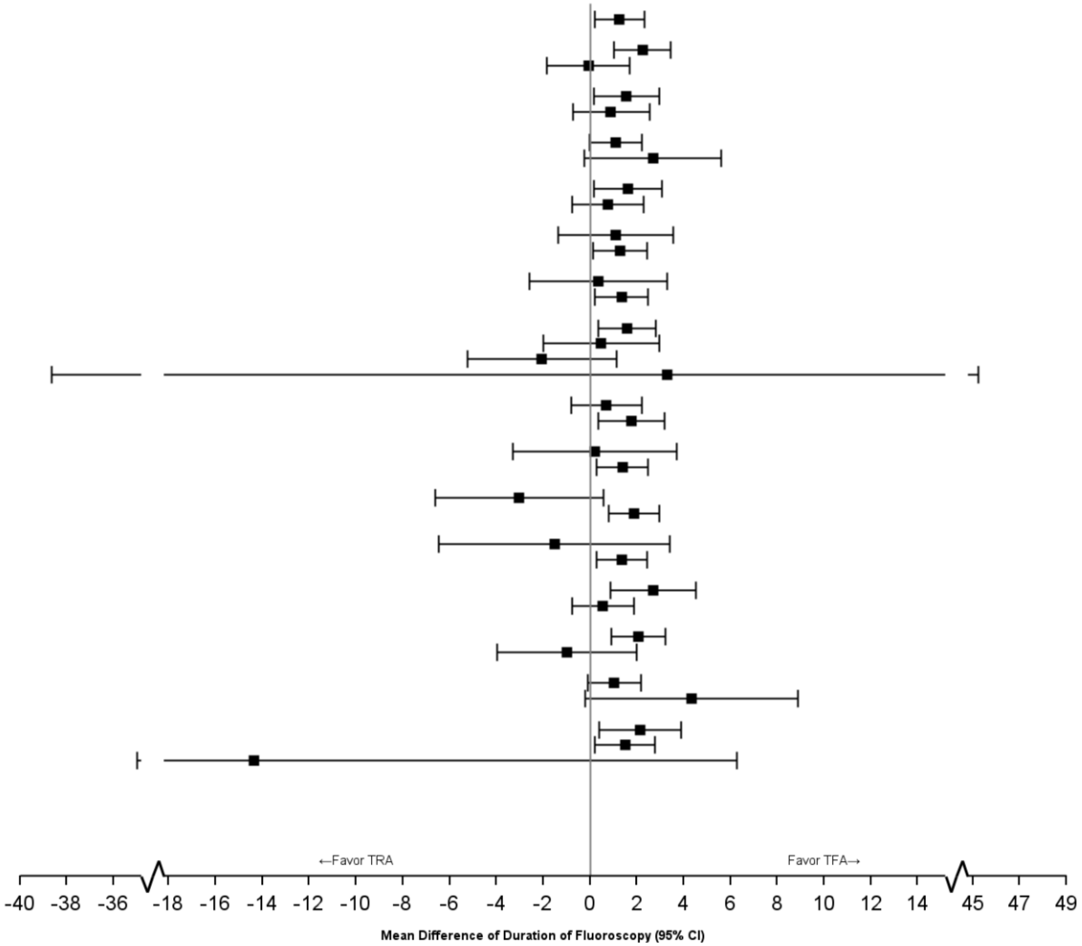

© 2026 Ni W et al. *JAMA Network Open*.

eFigure 7. Subgroup Analysis of Secondary Outcome (Bedridden Time)

Subgroup	TRA Mean(SD)/n	TFA Mean(SD)/n	Mean Difference (95% CI)
All subjects	188.40(852.686)/431	1079.04(371.776)/427	-890.64 (-988.42, -792.86)
Age			
< 60	229.06(1188.123)/210	1061.14(398.761)/235	-832.08 (-1002.46, -661.71)
>=60	149.77(277.430)/221	1100.94(335.545)/192	-951.17 (-1039.14, -863.21)
Sex			
Male	206.48(1104.042)/244	1102.49(380.580)/235	-896.01 (-1055.63, -736.39)
Female	164.82(295.695)/187	1050.34(359.614)/192	-885.52 (-977.26, -793.78)
Height			
<=175cm	197.42(885.599)/398	1079.06(377.950)/387	-881.64 (-986.90, -776.37)
>175cm	79.67(163.700)/33	1078.88(309.757)/40	-999.21 (-1202.14, -796.28)
BMI			
<=25	224.30(1073.142)/264	1077.81(388.073)/237	-853.52 (-1007.46, -699.57)
>25	131.66(231.345)/167	1080.57(351.400)/190	-948.90 (-1042.78, -855.03)
Smoking History			
Yes	164.39(332.740)/51	1141.53(396.681)/51	-977.14 (-1174.13, -780.15)
No	191.63(900.121)/380	1070.56(368.010)/376	-878.94 (-986.76, -771.11)
Alcohol Consumption History			
Yes	100.42(131.702)/38	1131.93(391.748)/43	-1031.51 (-1239.69, -823.33)
No	196.91(891.681)/393	1073.12(369.536)/384	-876.21 (-982.05, -770.37)
mRS			
Score 0	151.30(283.947)/337	1086.66(370.978)/341	-935.35 (-1005.84, -864.87)
Score 1	119.51(182.875)/76	1046.84(396.644)/70	-927.33 (-1073.48, -781.18)
Score 2	249.00(431.495)/17	1063.93(283.246)/15	-814.93 (-1148.13, -481.74)
Score 3	16897.00(-)/1	962.00(-)/1	15935.00 (-, -)
Hypertension			
Yes	237.89(1093.190)/255	1101.28(383.295)/236	-863.39 (-1020.34, -706.44)
No	116.70(207.932)/176	1051.56(356.110)/191	-934.86 (-1025.70, -844.01)
Diabetes			
Yes	206.47(361.101)/66	1079.18(334.698)/56	-872.71 (-1039.51, -705.91)
No	185.14(914.085)/365	1079.02(377.472)/371	-893.88 (-1004.58, -783.19)
Hyperlipidemia			
Yes	415.03(2121.575)/63	1179.20(367.146)/54	-764.17 (-1347.83, -180.52)
No	149.61(284.882)/368	1064.54(370.688)/373	-914.93 (-981.64, -848.22)
Coronary Artery Disease			
Yes	186.13(245.576)/15	1143.31(265.789)/16	-957.18 (-1266.30, -648.06)
No	188.49(866.786)/416	1076.54(375.312)/411	-888.05 (-988.89, -787.22)
Previous Puncture History			
Yes	154.94(295.567)/145	1037.01(372.615)/139	-882.07 (-988.94, -775.20)
No	205.37(1025.666)/286	1099.33(370.311)/288	-893.96 (-1030.26, -757.65)
Aortic Arch Classification			
Type I+II	194.30(993.460)/306	1061.91(361.544)/305	-867.61 (-995.92, -739.29)
Type III	156.45(313.536)/87	1119.11(416.061)/84	-962.66 (-1113.54, -811.77)
Aortic Arch Variation			
Normal	175.54(930.797)/349	1071.56(380.125)/352	-896.02 (-1011.20, -780.85)
Abnormal	268.32(424.196)/44	1099.97(315.155)/37	-831.65 (-1042.81, -620.50)
Supra-aortic Artery Tortuosity			
Normal	111.41(178.996)/83	1070.30(367.588)/80	-958.89 (-1096.16, -821.62)
Tortuous	206.18(1004.825)/304	1075.19(377.128)/305	-869.01 (-999.10, -738.92)
Kinking and Looping	163.29(277.360)/7	1081.80(319.106)/5	-918.51 (-1462.99, -374.04)

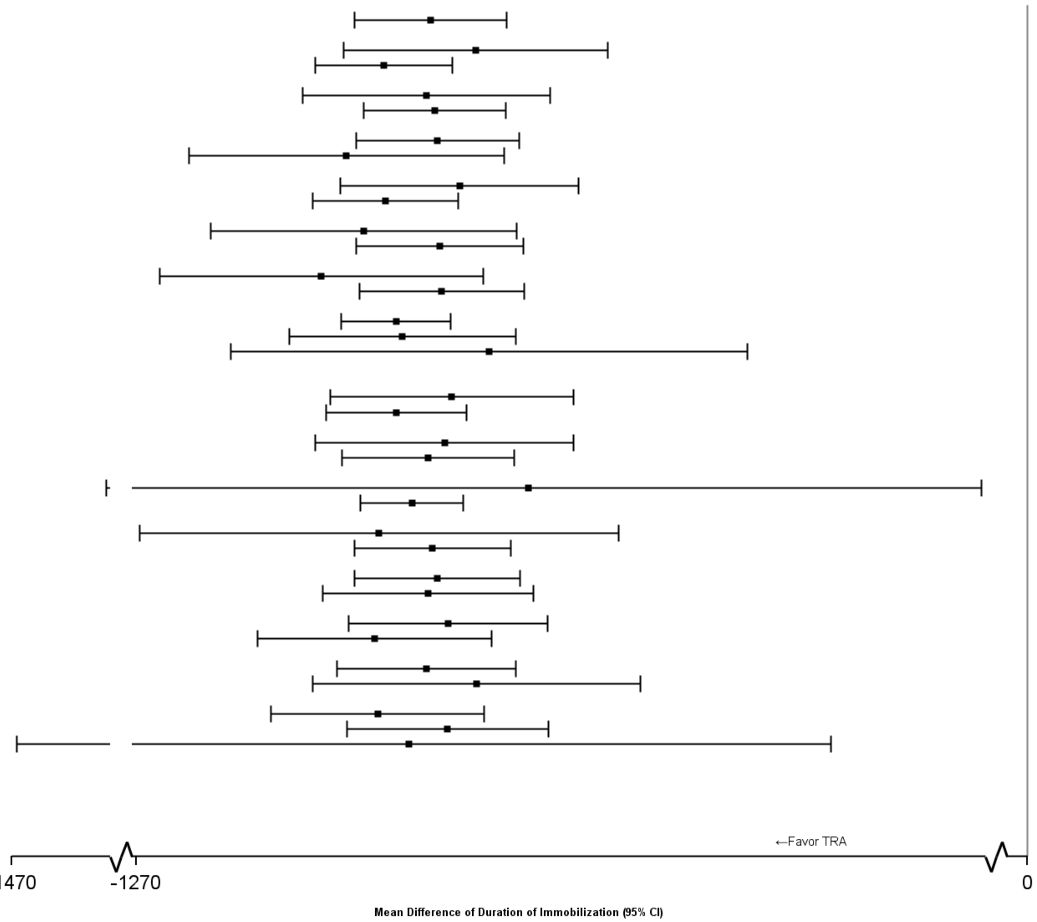

© 2026 Ni W et al. *JAMA Network Open*.

eFigure 8. Subgroup Analysis of Secondary Outcome (VAS Score)

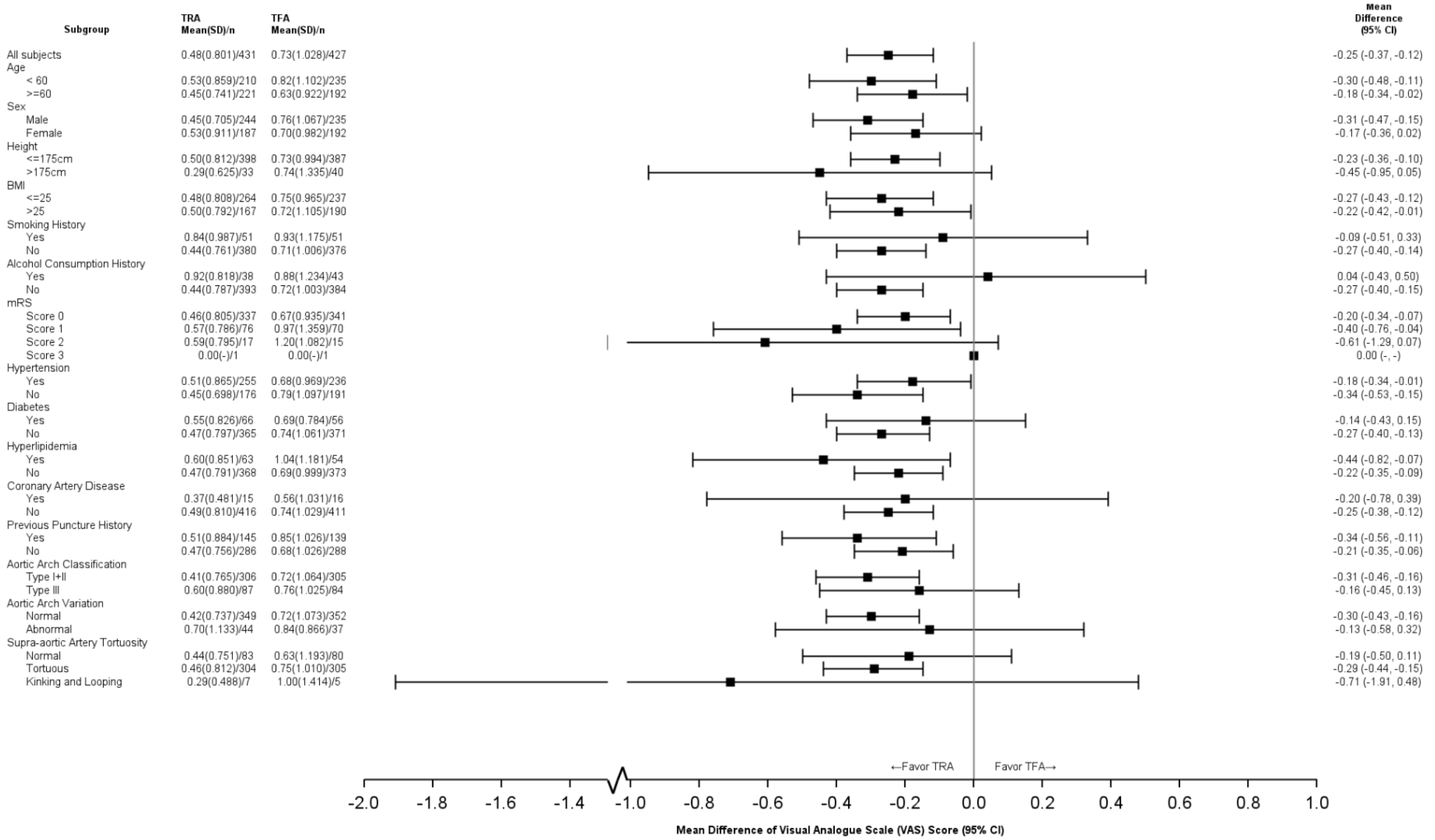

© 2026 Ni W et al. *JAMA Network Open*.

3. eTables

eTable 1. Additional Baseline and Procedural Characteristics

Characteristic	No. (%)	
	TRA group(n=431)	TFA group (n=427)
Prior Vascular Puncture History		
Left Radial artery	1 (0.7)	2 (1.4)
Right Radial artery	17 (11.7)	22 (15.8)
Left Femoral artery	6 (4.1)	9 (6.5)
Right Femoral artery	117 (80.7)	106 (76.3)
Other sites	7 (4.8)	6 (4.3)
History of Contrast Media Allergy		
yes	19(4.4)	29(6.8)
no	411(95.4)	398(93.2)
unknown	1(0.2)	0

© 2026 Ni W et al. *JAMA Network Open*.

eTable 2. Primary and Secondary Outcomes in Per-Protocol Analysis

Outcome	No. (%) TRA group (n=429)	TFA group (n=427)	Difference, % (95% CI) [*]	Relative risk (95% CI)[†]	P value
**Primary outcome**					
Success of diagnosis	392 (91.4)	409 (95.8)	-4.4 (-7.7, -1.1)	0.95 (0.92, 0.99)	0.36[‡]
**Failure Stage of primary outcome**[§]					
Stage 1	10(2.3)	0 (0)			
Stage 2	4 (0.9)	0 (0)			
Stage 3	3 (0.7)	0 (0)			
Stage 4	20 (4.7)	18 (4.2)			
**Secondary outcomes**					
Success of accurate diagnosis	340 (78.9)	389 (91.1)	-12.2 (-16.9, -7.5)	0.87 (0.82, 0.92)	0.998[‡]
Duration of angiography, min	38.65 (26.0 to 47.0)	33.73 (23.0 to 40.0)	4.92 (2.65, 7.18)		<0.0001[¶]
Duration of fluoroscopy, min	11.83(6.17 to 15.0)	10.57 (5.60 to 12.90)	1.26 (0.19, 2.32)		0.02 [¶]
bedridden time, min	188.40 (3.0 to 180.0)	1079.04 (842.0 to 1366.0)	-890.64(-998.42, -792.86)		<0.0001 [¶]
VAS score (within 24h post-procedure)[‖]	0.48 (0.00 to 1.00)	0.73 (0.00 to 1.00)	-0.25 (-0.37, -0.12)		<0.0001 [¶]

Data are n (%) or median (IQR)

Abbreviations: TRA, transarterial access; TFA, transfemoral access; VAS, visual analogue scale; NA, not applicable.

[*]Absolute incidence difference without adjustment.

[†]Generalized linear regression models with log link function.

[‡]The P value was calculated with the Wald test, non-inferiority test with a non-inferiority margin of -5%.

© 2026 Ni W et al. *JAMA Network Open*.

[§]Stage 1: Unable to insert the arterial sheath (small artery diameter, no blood return after arterial puncture, unable to advance the guidewire due to vasospasm, and atherosclerotic plaque, etc.); Stage 2: Puncture and sheath placement is successful, but the catheter or guidewire cannot be advanced (arterial tortuosity, vasospasm, and loop, etc.); Stage 3: The guidewire and catheter has reached the aortic arch, but the primary branch of the aortic arch cannot be selected; Stage 4: The guidewire and catheter has reached the primary branch of the aortic arch, but the procedure still cannot be completed successfully.

[¶]P value was calculated with the Linear regression model, with group as covariate.

[‖]Scores on the visual analogue scale of pain intensity range from 0 (no pain) to 10 (worst possible pain).

© 2026 Ni W et al. *JAMA Network Open*.

eTable 3. Primary and Secondary Outcomes in As-Treated Analysis

Outcome	No. (%) TRA group (n=413)	TFA group (n=445)	Difference, % (95% CI) [*]	Relative risk (95% CI)[†]	P value
**Primary outcome**					
The success rate of diagnosis	393 (95.2)	423 (95.1)	0.1 (-2.8, 3.0)	1.0 (0.97, 1.03)	0.0003[‡]
**Failure Stage of primary outcome** [§]					
Stage 1	0(0.0)	0 (0.0)			
Stage 2	0 (0.0)	0 (0.0)			
Stage 3	0(0.0)	0 (0.0)			
Stage 4	20 (4.8)	22 (4.9)			
**Secondary outcomes**					
The success rate of accurate diagnosis	341 (82.6)	400 (89.9)	-7.3 (-11.9, -2.7)	0.92 (0.87, 0.97)	0.84[‡]
Duration of angiography, min	37.48 (26.0 to 45.0)	35.01(23.0 to 41.0)	2.48 (0.20, 4.75)		0.03[¶]
Duration of fluoroscopy, min	11.78(6.25 to 14.90)	10.67(5.60 to 13.00)	1.12 (0.05 to 2.80)		0.04[¶]
bedridden time, min	172.44(3.0 to 160.0)	1099.00 (808.0 to 1361.0)	-885.39(-993.35 to -787.42)		<0.0001[¶]
VAS score (within 24h post-procedure) [‖]	0.46 (0.00 to 1.00)	0.75 (0.00 to 1.00)	-0.29(-0.41 to -0.17)		<0.0001[¶]

Data are n (%) or median (IQR)

Abbreviations: TRA, transarterial access; TFA, transfemoral access; VAS, visual analogue scale; NA, not applicable.

[*]Absolute incidence difference without adjustment.

[†]Generalized linear regression models with log link function.

[‡]The P value was calculated with the Wald test, non-inferiority test with a non-inferiority margin of -5%.

[§]Stage 1: Unable to insert the arterial sheath (small artery diameter, no blood return after arterial puncture, unable to advance the guidewire due to vasospasm, and atherosclerotic plaque, etc.); Stage 2: Puncture and sheath placement is successful, but the catheter or guidewire cannot be advanced (arterial tortuosity, vasospasm, and loop, etc.); Stage 3:

© 2026 Ni W et al. *JAMA Network Open*.

The guidewire and catheter has reached the aortic arch, but the primary branch of the aortic arch cannot be selected; Stage 4: The guidewire and catheter has reached the primary branch of the aortic arch, but the procedure still cannot be completed successfully.

¶The P value was calculated with the Linear regression model, with group as covariate.

‖Scores on the visual analogue scale of pain intensity range from 0 (no pain) to 10 (worst possible pain)[1].

© 2026 Ni W et al. *JAMA Network Open*.

eTable 4. Safety Outcomes Assessed by the CEC-ITT

Safety outcomes assessed by the CEC-ITT

**Outcomes**	No. (%)*		P Value †
	TRA Group （n=413）	TFA Group （n=445）	
Total	25 （6.1）	19 （4.3）	0.28
Access-related complications	23 （5.6）	17 （3.8）	0.26
Artery occlusion	20 (4.8)	1 (0.2)	
Subcutaneous Hematoma	2 (0.5)	8 (1.8)	
Pseudoaneurysm	1 (0.2)	4 (0.9)	
Catheter Kinking or Fracture	0 (0)	1 (0.2)	
Other	0 (0)	3(0.7)	
Neurological Complications	2 (0.5)	3 (0.7)	1.00
Contrast-Induced Encephalopathy	2 (0.5)	1 (0.2)	
Cerebral Infarction	0	1 (0.2)	
Vasovagal Reflex	0	1 (0.2)	

* The normal approximation method was used to calculate the 95%CI.

† P values were calculated using the chi-square test or Fisher's exact probability method.

© 2026 Ni W et al. *JAMA Network Open*.

eTable 5. Subgroup Analysis of Primary Outcome in Intention-to-Treat Analysis

	No.(95% CI) *			
Subgroup	TRA Group（n=431）	TFA Group（n=427）	RR(95% CI) †	P value‡
Gender				0.0457
Male	91.8 (88.4, 95.2)	93.6 (90.5, 96.7)	0.98 (0.93, 1.03)	
Female	89.8 (85.5, 94.2)	98.4 (96.7, 100.0)	0.91 (0.87, 0.96)	
Age				0.7545
< 60	91.9 (88.2, 95.6)	96.2 (93.7, 98.6)	0.96 (0.91, 1.00)	
≥60	90.0 (86.1, 94.0)	95.3 (92.3, 98.3)	0.94 (0.90, 1.00)	
Height				0.7066
> 175cm	87.9 (76.7, 99.0)	95.0 (88.2, 100.0)	0.93 (0.80, 1.07)	
≤175cm	91.2 (88.4, 94.0)	95.9（93.9,97.8）	0.95 (0.92, 0.99)	
BMI				0.7891
> 25	90.4 (86.0, 94.9)	94.7 (91.6, 97.9)	0.95 (0.90, 1.01）	
≤25	91.3 (87.9, 94.7)	96.6 (94.3, 98.9)	0.94 (0.90, 0.99)	
Smoking				0.1605
yes	94.1 (87.7, 100.0)	92.2 (84.8, 99.5)	1.02 (0.92, 1.13)	
no	90.5 (87.6, 93.5)	96.3 (94.4, 98.2)	0.94 (0.91, 0.98)	
Alcohol consumption				0.3858
yes	94.7 (87.6, 100.0)	95.3 (89.1, 100.0)	0.99 (0.90, 1.10)	
no	90.6 (87.7, 93.5)	95.8 (93.8, 97.8)	0.95（0.91,0.98）	
Hypertension				0.1549

© 2026 Ni W et al. *JAMA Network Open*.

yes	89.0 (85.2, 92.9)	95.8 (93.2, 98.3)	0.93 (0.88, 0.98)	
no	93.8 (90.2, 97.3)	95.8 (93.0, 98.7)	0.98 (0.93, 1.03)	
Diabetes				0.9071
yes	89.4 (82.0, 96.8)	94.6 (88.7, 100.0)	0.94 (0.85, 1.05)	
no	91.2 (88.3, 94.1)	96.0 (94.0, 98.0)	0.95 (0.92, 0.99)	
hyperlipedimia				0.8549
yes	88.9 (81.1, 96.6)	94.4 (88.3, 100.0)	0.94 (0.84, 1.05)	
no	91.3 (88.4, 94.2)	96.0 (94.0, 98.0)	0.95 (0.92, 0.99)	
Coronary Heart Disease				0.1983
yes	73.3 (51.0, 95.7)	93.8 (81.9, 100.0)	0.78 (0.56, 1.09)	
no	91.6 (88.9, 94.3)	95.9 (93.9, 97.8)	0.96 (0.92, 0.99)	
Previous puncture history				0.1726
yes	88.3 (83.0, 93.5)	96.4 (93.3, 99.5)	0.92 (0.86, 0.98)	
no	92.3 (89.2, 95.4)	95.5 (93.1, 97.9)	0.97 (0.93, 1.01)	
mRS score[§]				0.4224
0	90.8 (87.7, 93.9)	96.8 (94.9, 98.6)	0.94 (0.90, 0.98)	
1	92.1 (86.0, 98.2)	91.4 (84.9, 98.0)	1.01 (0.91, 1.11)	
2	88.2 (72.9, 100.0)	93.3 (80.7, 100.0)	0.95 (0.76, 1.18)	
3	100.0 (100.0, 100.0)	100.0 (100.0, 100.0)	/	/

[*] The normal approximation method was used to calculate the 95%CI.

[†] Logistic regression was used to calculate RR and 95%CI between groups, and only treatment group was included as covariates.

[‡] The P-value for the subgroup-by-treatment interaction term in logistic regression was calculated using the likelihood ratio test.

[§] Scores on the mRS range from 0 (no functional limitations) to 6 (death), with higher scores indicating more severe functional disability. A score of 2 or less indicates functional independence[2].

© 2026 Ni W et al. *JAMA Network Open*.

eTable 6. Subgroup Analysis of Primary Outcome in Per-Protocol Analysis

Subgroup analysis of primary outcome（success rate of diagnostic cerebral angiography） in per-protocol analysis

Subgroup	No.(95% CI) *		RR(95% CI) †	P value of non-inferiority test ‡
	TRA Group（n=429）	TFA Group（n=427）		
Gender				0.0457
Male	91.8 (88.4, 95.2)	93.6 (90.5, 96.7)	0.98 (0.93, 1.03)	
Memale	90.8（86.6,95.0）	98.4 (96.7, 100.0)	0.92（0.88,0.97）	
Age				0.7545
< 60	91.9 (88.2, 95.6)	96.2 (93.7, 98.6)	0.96 (0.91, 1.00)	
≥60	90.9（87.1,94.7）	95.3 (92.3, 98.3)	0.95（0.90,1.00）	
Height				0.7066
> 175cm	87.9 (76.7, 99.0)	95.0 (88.2, 100.0)	0.93 (0.80, 1.07)	
≤175cm	91.7（88.9,94.4）	95.9（93.9,97.8）	0.96（0.92,0.99）	
BMI				0.7891
> 25	90.4 (86.0, 94.9)	94.7 (91.6, 97.9)	0.95 (0.90, 1.01）	
≤25	92.0（88.7,95.3）	96.6 (94.3, 98.9)	0.95（0.91,0.99）	
Smoking				0.1605
yes	94.1 (87.7, 100.0)	92.2 (84.8, 99.5)	1.02 (0.92, 1.13)	
no	91.0（88.1,93.9）	96.3 (94.4, 98.2)	0.95（0.91,0.98）	
Alcohol consumption				0.3858
yes	94.7 (87.6, 100.0)	95.3 (89.1, 100.0)	0.99 (0.90, 1.10)	
no	91.0（88.2,93.9）	95.8 (93.8, 97.8)	0.95 (0.92, 0.99)	
Hypertension				0.1549
yes	89.7（86.0,93.5）	95.8 (93.2, 98.3)	0.94（0.89,0.98）	
no	93.8 (90.2, 97.3)	95.8 (93.0, 98.7)	0.98 (0.93, 1.03)	

© 2026 Ni W et al. *JAMA Network Open*.

Diabetes				0.9071
yes	90.8（83.7,97.8）	94.6 (88.7, 100.0)	0.96（0.87,1.06）	
no	91.5（88.6,94.4）	96.0 (94.0, 98.0)	0.95 (0.92, 0.99)	
Hyperlipedimia				0.8549
yes	88.9 (81.1, 96.6)	94.4 (88.3, 100.0)	0.94 (0.84, 1.05)	
no	91.8（89.0,94.6）	96.0 (94.0, 98.0)	0.96（0.92,0.99）	
Coronary Heart Disease				0.1983
yes	73.3 (51.0, 95.7)	93.8 (81.9, 100.0)	0.78 (0.56, 1.09)	
no	92.0（89.4,94.6）	95.9 (93.9, 97.8)	0.96 (0.93, 0.99)	
Previous puncture history				0.1726
yes	88.3 (83.0, 93.5)	96.4 (93.3, 99.5)	0.92 (0.86, 0.98)	
no	93.0（90.0,95.9）	95.5 (93.1, 97.9)	0.97 (0.93, 1.01)	
mRS score				0.4224
0	91.3（88.3,94.4）	96.8 (94.9, 98.6)	0.94 (0.91, 0.98)	
1	92.1 (86.0, 98.2)	91.4 (84.9, 98.0)	1.01 (0.91, 1.11)	
2	88.2 (72.9, 100.0)	93.3 (80.7, 100.0)	0.95 (0.76, 1.18)	
3	100.0 (100.0, 100.0)	100.0 (100.0, 100.0)	/	/

* The normal approximation method was used to calculate the 95%CI.

†Logistic regression was used to calculate RR and 95%CI between groups, and only treatment group was included as covariates.

‡The P-value for the subgroup-by-treatment interaction term in logistic regression was calculated using the likelihood ratio test.

© 2026 Ni W et al. *JAMA Network Open*.

4. eReferences

1. Zusman M. The Absolute Visual Analogue Scale (AVAS) as a Measure of Pain Intensity. Aust J Physiother 1986;32:244-246.

2. van Swieten JC, Koudstaal PJ, Visser MC, Schouten HJ, van Gijn J. Interobserver agreement for the assessment of handicap in stroke patients. Stroke 1988;19:604-607.

© 2026 Ni W et al. *JAMA Network Open*.
