# Supplementary material for: Transradial vs Transfemoral Access for Cerebral Angiography: A Randomized Noninferiority Clinical Trial
Source: JAMA Netw Open. 2026 Mar 19;9(3):e261929. doi: 10.1001/jamanetworkopen.2026.1929 (PMC13003373; doi:10.1001/jamanetworkopen.2026.1929)
Supplement: Supplement 3. — Nonauthor Collaborators [file jamanetwopen-e261929-s003.pdf]

Supplemental Online Content: Nonauthor Collaborators
*First name, last name, and suffix (if applicable) are required and will appear in PubMed.

*Group Name(s): TRACE Investigators							
***First Name and Middle Initial(s)**	***Last Name**	***Suffix (eg, Jr, III)**	Academic Degrees	Institution	Location (city, state/province, country)	Role or Contribution, eg, chair, principal investigator	Group (if more than 1 Group listed in the byline) and/or Subgroup (eg, Steering Committee)
Zhiwen	Jiang		M.D.	Department of Neurosurgery, Huashan Hospital, Shanghai Medical College, Fudan University	Shanghai,China	Data collection,Site coordination	Clinical Site Coordinators
Ruiyuan	Weng		M.D.	Department of Neurosurgery, Huashan Hospital, Shanghai Medical College, Fudan University	Shanghai,China	Data collection,Site coordination	Clinical Site Coordinators
Yuchao	Fei		M.D.	Department of Neurosurgery, Huashan Hospital, Shanghai Medical College, Fudan University	Shanghai,China	Data collection,Site coordination	Clinical Site Coordinators
Zeran	Yu		M.D.	Department of Neurosurgery, Huashan Hospital, Shanghai Medical College, Fudan University	Shanghai,China	Data collection,Site coordination	Clinical Site Coordinators
Zhou	Hu		M.D.	Department of Neurosurgery, Huashan Hospital, Shanghai Medical College, Fudan University	Shanghai,China	Data collection,Site coordination	Clinical Site Coordinators
Yingtao	Liu		M.D.	Department of Radiology, Huashan Hospital, Shanghai Medical College, Fudan University, Shanghai	Shanghai,China	Data collection,Site coordination	Clinical Site Coordinators
Jun	Ying		M.D.	Department of Neurosurgery, Huashan Hospital, Shanghai Medical College, Fudan University	Shanghai,China	Data collection,Site coordination	Clinical Site Coordinators

*First name, last name, and suffix (if applicable) are required and will appear in PubMed.

***First Name and Middle Initial(s)**	***Last Name**	***Suffix (eg, Jr, III)**	Academic Degrees	Institution	Location (city, state/province, country)	Role or Contribution, eg, chair, principal investigator	Group (if more than 1 Group listed in the byline) and/or Subgroup (eg, Steering Committee)
Huan	Yang		M.D.	Department of Nursing, Huashan Hospital, Shanghai Medical College, Fudan University	Shanghai,China	Data collection,Site coordination	Clinical Site Coordinators
Yuxin	Cheng		M.D.	Department of Nursing, Huashan Hospital, Shanghai Medical College, Fudan University	Shanghai,China	Data collection,Site coordination	Clinical Site Coordinators
Ronghua	Chen		M.D.	Department of Neurosurgery,The First People's Hospital Of Changzhou	Changzhou,Jiangsu,China	Data collection,Site coordination	Clinical Site Coordinators
Junjun	Zhang		M.D.	Department of Neurosurgery,The First Affiliated Hospital of Ningbo University	Ningbo，Zhejiang，China	Data collection,Site coordination	Clinical Site Coordinators
Linsun	Dai		M.D.	Department of Neurosurgery,The First Affiliated Hospital of Fujian Medical University	Fuzhou,Fujian,China	Data collection,Site coordination	Clinical Site Coordinators
Xiaoyu	Lin		M.D.	Department of Neurosurgery,Panvascular Disease Management Center，Wenzhou Central Hospital	Wenzhou,Zhejiang,China	Data collection,Site coordination	Clinical Site Coordinators
Quan	Gu		M.D.	Department of Neurosurgery,Affiliated Zhongshan Hospital of Fudan University，Qingpu Branch	Shanghai,China	Data collection,Site coordination	Clinical Site Coordinators
Lin	Zhou		M.D.	Department of Neurosurgery,Affiliated Zhongshan Hospital of Fudan University，Qingpu Branch	Shanghai,China	Data collection,Site coordination	Clinical Site Coordinators

*First name, last name, and suffix (if applicable) are required and will appear in PubMed.

***First Name and Middle Initial(s)**	***Last Name**	***Suffix (eg, Jr, III)**	Academic Degrees	Institution	Location (city, state/province, country)	Role or Contribution, eg, chair, principal investigator	Group (if more than 1 Group listed in the byline) and/or Subgroup (eg, Steering Committee)
Liyong	Zhang		M.D.	Department of Neurosurgery,Liaocheng People's Hospital	Liaocheng,Shandong,China	Data collection,Site coordination	Clinical Site Coordinators
Meng	Zhang		M.D.	Department of Neurosurgery,Liaocheng People's Hospital	Liaocheng,Shandong,China	Data collection,Site coordination	Clinical Site Coordinators
Zidong	Wang		M.D.	Department of Neurosurgery,Liaocheng People's Hospital	Liaocheng,Shandong,China	Data collection,Site coordination	Clinical Site Coordinators
Fukai	Ma		M.D.	Department of Neurosurgery,Ninth People Hospital Affiliated to Shanghai Jiao Tong University School of Medicine	Shanghai,China	Data collection,Site coordination	Clinical Site Coordinators
Yang	Wang		M.D.	Department of Neurosurgery,Ninth People Hospital Affiliated to Shanghai Jiao Tong University School of Medicine	Shanghai,China	Data collection,Site coordination	Clinical Site Coordinators
Xitao	Zong		M.D.	Department of Neurosurgery,Southern Central Hospital Of Yunnan Province	Gejiu,Yunnan,China	Data collection,Site coordination	Clinical Site Coordinators
Lin	Li		M.D.	Department of Neurosurgery,Southern Central Hospital Of Yunnan Province	Gejiu,Yunnan,China	Data collection,Site coordination	Clinical Site Coordinators
Ning	Wang		M.D.	Department of Neurointerventional,Zhejiang Hospital	Hangzhou,Zhejiang,China	Data collection,Site coordination	Clinical Site Coordinators
Yuhai	Gao		M.D.	Department of Neurointerventional,Zhejiang Hospital	Hangzhou,Zhejiang,China	Data collection,Site coordination	Clinical Site Coordinators

Supplemental Online Content: Nonauthor Collaborators
*First name, last name, and suffix (if applicable) are required and will appear in PubMed.

***First Name and Middle Initial(s)**	***Last Name**	***Suffix (eg, Jr, III)**	Academic Degrees	Institution	Location (city, state/province, country)	Role or Contribution, eg, chair, principal investigator	Group (if more than 1 Group listed in the byline) and/or Subgroup (eg, Steering Committee)
Jixi	Xie		M.D.	Department of Neurosurgery,SIR RUN RUN SHAW HOSPITAL	Hangzhou,Zhejiang,China	Data collection,Site coordination	Clinical Site Coordinators
Xin	Lou		M.D.	Department of Neurosurgery,SIR RUN RUN SHAW HOSPITAL	Hangzhou,Zhejiang,China	Data collection,Site coordination	Clinical Site Coordinators
Haiping	Ling		M.D.	Department of Neurosurgery,Nanjing Drum Tower Hospital, Affiliated Hospital of Medical School, Nanjing University	Nanjing,Jiangsu,China	Data collection,Site coordination	Clinical Site Coordinators
Shijie	Na		M.D.	Department of Neurosurgery,Nanjing Drum Tower Hospital, Affiliated Hospital of Medical School, Nanjing University	Nanjing,Jiangsu,China	Data collection,Site coordination	Clinical Site Coordinators
Bingbo	Lv		M.D.	Department of Neurosurgery, Qilu Hospital Of Shandong University Dezhou Hospital	Dezhou,Shandong,China	Data collection,Site coordination	Clinical Site Coordinators
Baoxin	Ren		M.D.	Department of Neurosurgery, Qilu Hospital Of Shandong University Dezhou Hospital	Dezhou,Shandong,China	Data collection,Site coordination	Clinical Site Coordinators
Liqun	Jiao		M.D.	Department of Neurosurgery and Interventional Neuroradiology, Xuanwu Hospital, China International Neuroscience Institute, Capital Medical University, China National Clinical Research Center for Neurological Diseases	Beijing,China	Member of Data and Safety Monitoring Board	Data and Safety Monitoring Board (DSMB)

Supplemental Online Content: Nonauthor Collaborators
*First name, last name, and suffix (if applicable) are required and will appear in PubMed.

*First Name and Middle Initial(s)	*Last Name	*Suffix (eg, Jr, III)	Academic Degrees	Institution	Location (city, state/province, country)	Role or Contribution, eg, chair, principal investigator	Group (if more than 1 Group listed in the byline) and/or Subgroup (eg, Steering Committee)
Zhangsheng	Yu		M.D.	Department of Bioinformatics and Biostatistics, School of Life Sciences and Biotechnology, Shanghai Jiao Tong University	Shanghai,China	Member of Data and Safety Monitoring Board	Data and Safety Monitoring Board (DSMB)
Lei	Huang		M.D.	Department of Radiology,Parkway Shanghai Hospital	Shanghai,China	Chair of Imaging Core Laboratory	Imaging Core-lab
Jun	Shi		B.A.	Clinical Consultancy Research Center	Beijing,China	Assistant of Imaging Core Laboratory	Imaging Core-lab
Qian	Sun		B.A.	Clinical Consultancy Research Center	Shanghai,China	Assistant of Imaging Core Laboratory	Imaging Core-lab
Gongjian	Guo			Clinical Consultancy Research Center	Shanghai,China	Contract Research Organization	Contract Research Organization(CRO)
Qianhui	Yuan			Clinical Consultancy Research Center	Shanghai,China	Contract Research Organization	Contract Research Organization(CRO)
Runzhi	Ma			Clinical Consultancy Research Center	Shanghai,China	Contract Research Organization	Contract Research Organization(CRO)
Xuehui	Cao			Clinical Consultancy Research Center	Shanghai,China	Contract Research Organization	Contract Research Organization(CRO)
Hongxin	Xu			Clinical Consultancy Research Center	Shanghai,China	Contract Research Organization	Contract Research Organization(CRO)
Qiming	Chen			Clinical Consultancy Research Center	Shanghai,China	Contract Research Organization	Contract Research Organization(CRO)
Ye	Wang			Clinical Consultancy Research Center	Shanghai,China	Contract Research Organization	Contract Research Organization(CRO)
Chaoqing	Sun			Clinical Consultancy Research Center	Shanghai,China	Contract Research Organization	Contract Research Organization(CRO)
Na	Li			Shanghai Luoke Medical Information Consulting Co., Ltd.	Shanghai,China	Site Management Organization	Site Management Organization

Supplemental Online Content: Nonauthor Collaborators
*First name, last name, and suffix (if applicable) are required and will appear in PubMed.

***First Name and Middle Initial(s)**	***Last Name**	***Suffix (eg, Jr, III)**	Academic Degrees	Institution	Location (city, state/province, country)	Role or Contribution, eg, chair, principal investigator	Group (if more than 1 Group listed in the byline) and/or Subgroup (eg, Steering Committee)
Yuanyuan	Liu			Shanghai Luoke Medical Information Consulting Co., Ltd.	Shanghai,China	Site Management Organization	Site Management Organization
Tingting	Xie			Shanghai Luoke Medical Information Consulting Co., Ltd.	Shanghai,China	Site Management Organization	Site Management Organization
Zhuo	Feng			Shanghai Luoke Medical Information Consulting Co., Ltd.	Shanghai,China	Site Management Organization	Site Management Organization
Lin	Pan			Shanghai Luoke Medical Information Consulting Co., Ltd.	Changzhou,Jiangsu,China	Site Management Organization	Site Management Organization
Wenyi	Wu			Shanghai Luoke Medical Information Consulting Co., Ltd.	Ningbo，Zhejiang，China	Site Management Organization	Site Management Organization
Siyu	Weng			Shanghai Luoke Medical Information Consulting Co., Ltd.	Fuzhou,Fujian,China	Site Management Organization	Site Management Organization
Yuanyuan	Wang			Shanghai Luoke Medical Information Consulting Co., Ltd.	Wenzhou,Zhejiang,China	Site Management Organization	Site Management Organization
Xiaoqian	Chu			Shanghai Luoke Medical Information Consulting Co., Ltd.	Shanghai,China	Site Management Organization	Site Management Organization
Xinyue	Chen			Shanghai Luoke Medical Information Consulting Co., Ltd.	Dezhou,Shandong,China	Site Management Organization	Site Management Organization
Tao	Shen			Shanghai Luoke Medical Information Consulting Co., Ltd.	Shanghai,China	Site Management Organization	Site Management Organization
Yun	Long			Shanghai Luoke Medical Information Consulting Co., Ltd.	Gejiu,Honghe,China	Site Management Organization	Site Management Organization
Tingting	Wang			Shanghai Luoke Medical Information Consulting Co., Ltd.	Hangzhou,Zhejiang,China	Site Management Organization	Site Management Organization
Miao	Fang			Shanghai Luoke Medical Information Consulting Co., Ltd.	Hangzhou,Zhejiang,China	Site Management Organization	Site Management Organization
Weiwen	Tang			Shanghai Luoke Medical Information Consulting Co., Ltd.	Nanjing,Jiangsu,China	Site Management Organization	Site Management Organization
Cengceng	Shang			Shanghai Luoke Medical Information Consulting Co., Ltd.	Dezhou,Shandong,China	Site Management Organization	Site Management Organization
