# Supplementary material for: Transradial vs Transfemoral Access for Cerebral Angiography: A Randomized Noninferiority Clinical Trial
Source: JAMA Netw Open. 2026 Mar 19;9(3):e261929. doi: 10.1001/jamanetworkopen.2026.1929 (PMC13003373; doi:10.1001/jamanetworkopen.2026.1929)
Supplement: Supplement 4. — Data Sharing Statement [file jamanetwopen-e261929-s004.pdf]

Data Sharing Statement

Ni. Transradial vs Transfemoral Access for Cerebral Angiography. *JAMA Netw Open*.
Published March 19, 2026. doi:10.1001/jamanetworkopen.2026.1929

Data
Additional Information: Transradial Versus Transfemoral Access for Cerebral Angiography; ClinicalTrials.gov Identifier: NCT05401669
Data available: No
